# BALANCING BIOLOGICAL AND BIOMECHANICAL PERFORMANCE IN INTERVERTEBRAL DISC REPAIR: A SYSTEMATIC REVIEW OF INJECTABLE CELL DELIVERY BIOMATERIALS

**DOI:** 10.22203/eCM.v040a15

**Published:** 2020-11-18

**Authors:** C.J. Panebianco, J.H. Meyers, J. Gansau, W.W. Hom, J.C. Iatridis

**Affiliations:** Leni and Peter W. May Department of Orthopaedics, Icahn School of Medicine at Mount Sinai, New York, NY, USA

**Keywords:** Intervertebral disc, annulus fibrosus, nucleus pulposus, biocompatible materials, biomaterials, hydrogels, tissue engineering, regenerative medicine, cell- and tissue-based therapy, cell delivery

## Abstract

Discogenic back pain is a common condition without approved intervertebral disc (IVD) repair therapies. Cell delivery using injectable biomaterial carriers offers promise to restore disc height and biomechanical function, while providing a functional niche for delivered cells to repair degenerated tissues. This systematic review advances the injectable IVD cell delivery biomaterials field by characterising its current state and identifying themes of promising strategies. Preferred Reporting Items for Systematic Reviews and Meta-Analyses (PRISMA) guidelines were used to screen the literature and 183 manuscripts met the inclusion criteria. Cellular and biomaterial inputs, and biological and biomechanical outcomes were extracted from each study. Most identified studies targeted nucleus pulposus (NP) repair. No consensus exists on cell type or biomaterial carrier, yet most common strategies used mesenchymal stem cell (MSC) delivery with interpenetrating network/co-polymeric (IPN/CoP) biomaterials composed of natural biomaterials. All studies reported biological outcomes with about half the studies reporting biomechanical outcomes. Since the IVD is a load-bearing tissue, studies reporting compressive and shear moduli were analysed and two major themes were found. First, a competitive balance, or ‘seesaw’ effect, between biomechanical and biological performance was observed. Formulations with higher moduli had inferior cellular performance, and vice versa. Second, several low-modulus biomaterials had favourable biological performance and matured throughout culture duration with enhanced extracellular matrix synthesis and biomechanical moduli. Findings identify an opportunity to develop next-generation biomaterials that provide high initial biomechanical competence to stabilise and repair damaged IVDs with a capacity to promote cell function for long-term healing.

## Introduction

Low back and neck pain are pressing public health concerns, estimated to affect up to 80 % of the adult population during their lifetimes (Hoy *et al*., 2014; Rubin, 2007). Between 1990 and 2015, the years lived with disability caused by low back pain increased by 54 % globally, making it the leading cause of disability (Vos *et al*., 2016). In 2016, low back and neck pain ranked highest, amongst 154 high-cost conditions, for healthcare spending in the United States. Costing $ 134.5 billion, more money was spent treating low back and neck pain than other musculoskeletal disorders, diabetes, cardiovascular disease or cancer (Dieleman *et al*., 2020). Chronic low back pain is a complex condition with multiple contributors, and treatment options have limited efficacy (Hartvigsen *et al*., 2018). Pathologies of the intervertebral disc (IVD) are strongly associated with back pain, and IVD involvement is estimated in 39 – 42 % of back pain patients, making the IVD a prime therapeutic target (Adams and Dolan, 2012; DePalma *et al*., 2011; Livshits *et al*., 2011; Ohtori *et al*., 2015).

The IVD is a fibrocartilaginous tissue that bears high-magnitude spine loads and permits three-dimensional motions of the spinal column (Shapiro and Risbud, 2014). These functions are made possible by the complex structural features of the IVD. The outer ring of the IVD is a fibre-reinforced, angle-ply laminate called the annulus fibrosus (AF), which consists of highly organised collagen lamellae. The AF gradually transitions from its outer region – which is primarily composed of type I collagen with elongated, fibroblast-like cells – to the inner region consisting of rounded fibrocartilage cells surrounded by a matrix of type I and II collagen (Eyre and Muir, 1976; Torre *et al*., 2019). The centrally located core of the IVD is the nucleus pulposus (NP); a gelatinous, proteoglycan-rich structure with rounded, chondrocyte-like cells in a randomly-oriented type II collagen network (Pattappa *et al*., 2012; Shapiro and Risbud, 2014). In a healthy state, the highly pressurised NP is contained by the fibrous AF so the IVD may resist large axial loads with high pressurisation and smaller matrix strains (Iatridis *et al*., 2013; Miele *et al*., 2012). With aging and degeneration, IVD hydration, proteoglycan content, and extracellular matrix (ECM) synthesis decrease, while collagen crosslinking and the presence of tears and defects increase (Adams and Roughley, 2006; Antoniou *et al*., 1996; Duance *et al*., 1998; Nerlich *et al*., 1997). Overloading and aging of the IVD can accelerate cell-mediated degeneration of the IVD, resulting in impaired biomechanical function, structural failure, and painful conditions (Adams and Roughley, 2006; Iatridis *et al*., 2013).

Identifying the aetiology of back pain, and distinguishing aging and degeneration, remain unmet clinical challenges. Due to these complexities, current treatment options for patients experiencing discogenic back pain, (*i.e.* back pain where IVD degeneration is the most prominent diagnosis) have limited efficacy. Current guidelines recommend self-management, physical and psychosocial therapies, and some forms of complementary medicine as the first-line treatment option for acute and persistent low back pain. Clinical guidelines for interventional surgery vary (National Guideline Centre (UK), 2016; Qaseem *et al*., 2017; Stochkendahl *et al*., 2018), but if a patient continues to experience persistent pain with radiographic evidence of herniation, spinal stenosis or IVD degeneration, they may be candidates for surgical interventions (Foster *et al*., 2018). However, surgeries are primarily palliative and often fail because they do not target root causes of IVD degeneration. For example, the neuropathy, disability and pain associated with IVD herniation can be treated with a discectomy surgery. Discectomy procedures remove herniated NP tissue from the IVD, but do not seal AF defects caused by the herniation, which can lead to accelerated degeneration, reherniation and recurrent pain (Carragee *et al*., 2003; Gray *et al*., 2006; Lurie *et al*., 2014; Parker *et al*., 2015; Schroeder *et al*., 2012). Spinal fusion and total disc replacement can be effective treatments for late-stage degeneration (*i.e.* Thompson Grade IV/V) when well indicated, but altered biomechanical loading and can lead to degeneration of IVDs adjacent to the spinal fusion site, called adjacent segment disease (Geisler *et al*., 2009; Ghiselli *et al*., 2004; Phillips *et al*., 2013; Siepe *et al*., 2014; Xia *et al*., 2013). The significant drawbacks in current treatments for discogenic back pain and poor intrinsic healing capacity of the IVD highlight a critical need to develop next-generation IVD therapies that are capable of slowing down the progression of early IVD degeneration and promoting repair.

Cell therapy is a promising treatment for progressive IVD degeneration since it has shown efficacy in numerous preclinical and clinical studies (Sakai, 2011; Schol and Sakai, 2019). Exogenous cells can promote ECM synthesis, and secrete paracrine signals that may stimulate endogenous IVD cells to synthesise ECM and release immunomodulatory signals to combat excessive inflammation (Clouet *et al*., 2019). One concern of IVD cell delivery is cell-leakage and off-targeting effects. Vadalà *et al*. demonstrated that injection of mesenchymal stem cells (MSCs) with saline into an *in vivo* rabbit model of IVD degeneration resulted in ectopic osteophyte formation (Vadalà *et al*., 2012). Aligning with the classical tissue engineering paradigm (Langer and Vacanti, 1993), injectable cell delivery biomaterials may enhance cell delivery therapies by retaining injected cells at the injury site and providing an instructive microenvironment that supports cell viability and cell-mediated healing (Burdick *et al*., 2016). Injectable biomaterials have potential for clinical translation because they are minimally invasive, gel *in situ*, and fill irregularly-shaped defects. Many of these biomaterials can also be tuned to mimic the biomechanical properties of their target tissue, which is considered particularly important for repair of the IVD and other musculoskeletal tissues that provide a predominantly mechanical function (Bowles and Setton, 2017; Guterl *et al*., 2013).

Barriers to the clinical adoption of injectable IVD cell delivery repair strategies include lack of a consensus on which cell source and delivery vehicle are optimal for this therapeutic strategy, and a need to prevent reherniation of any injected biomaterial (Benneker *et al*., 2014; Iatridis *et al*., 2013; Smith *et al*., 2018). To help overcome these obstacles, a systematic review of the literature on injectable biomaterials used for cell delivery in IVD repair was conducted. The first goal of this systematic review was to define the state of the field for cell delivery biomaterials designed for IVD repair. Second, this review identified the most commonly reported biological and biomechanical outcome measurements used to assess published strategies. Third, the effect of cell source and biomaterial carrier choice on the biological and biomechanical performance of published IVD repair strategies was determined to identify common themes for successful repair strategies. It is hoped that this summary and synthesis of IVD cell delivery biomaterial literature will help accelerate their clinical translation to reduce the burden of chronic discogenic back pain.

## Materials and Methods

### Identification of peer-reviewed manuscripts for analysis

Identification and retrieval of literature was conducted using the Preferred Reporting Items for Systematic Reviews and Meta-Analyses (PRISMA) guidelines (Moher *et al*., 2009). A comprehensive search, using controlled language terms and keywords, was conducted in MEDLINE® Ovid, Embase Ovid and Scopus from the date of database inception through April 9^th^, 2020. Search criteria, agreed upon before executing the search, were established to include controlled language terms and keywords related to “biocompatible materials” AND “intervertebral disc” AND “cell- and tissue-based therapy”. The full search query for MEDLINE Ovid and Embase Ovid is available in Appendix A. Initial results yielded a total of 5,128 articles across all databases. Search results were exported into Covidence software (Melbourne, Australia) for deduplication and screening. Deduplication in Covidence removed 1,371 duplicate articles. Titles and abstracts of the remaining 3,757 articles were screened by 2 independent reviewers using Covidence. If conflicts arose, the full-text manuscript was reviewed to arrive at a consensus. Screening excluded:

- duplicate studies not identified by Covidence;
- studies in languages other than English;
- non-full text original research articles;
- studies that were irrelevant to the topic of intervertebral disc (IVD) repair;
- studies that used acellular biomaterials not intended for cell delivery, carrier-free cell delivery systems and non-injectable tissue engineered constructs.

Based on these inclusion/exclusion criteria, a total of 183 full-length, peer-reviewed manuscripts were identified.

### Data extraction and analysis

Before reviewing the identified manuscripts, specific categories for analysis were defined related to the cellular and biomaterial inputs of the IVD repair strategy and the biological and biomechanical outcome measures. Cellular inputs included: target tissue (AF, NP or Both), cell type, cell species and mode of delivery. Biomaterial inputs included: biomaterial composition, method of additional crosslinking and additional modifications to the cell-biomaterial delivery strategy (*i.e.* biological functionalisation, growth factor delivery, gene delivery, co-culture/pre-conditioning, and ‘Other’). Biomaterial formulations were referred to using the same language as the original article, for consistency. Concentrations were reported as they were reported in the original article, because not all manuscripts provided sufficient information for unit conversions. Biological functionalisation was defined as the modification of a biomaterial carrier with molecules that promote cell adhesion, proliferation, ECM synthesis, *etc*. Biological outcomes extracted included: validation method (*i.e. in vitro, ex vivo* and/or *in vivo*), viability, proliferation, gene expression, ECM synthesis, and gross morphology (*i.e.* assessment of cell morphology *in vitro* or tissue morphology *ex vivo* or *in vivo*). Biomechanical outcomes extracted included: validation method, gelation kinetics, hydrogel degradation, swelling, compressive testing, viscoelastic testing, tensile testing, failure mechanical testing, and disc height changes.

Aside from the validation method, each category was assigned a “yes/no” binary. If a study was assigned a “yes,” details on the specific method of analysis performed and results related to that outcome were noted (*e.g.* assay used to assess viability and the outcome of the viability assessment). If an outcome was noted that did not fall into the categories mentioned, it was assigned to an “Other” category for biological or biomechanical outcomes, and its details were noted. Manuscripts were randomly assigned to a total of four independent reviewers, who read each paper in detail and noted responses for each category. When questions arose (*e.g.* a measure was not clearly defined), the manuscript was reviewed by an additional reader to arrive at a consensus. Data were tabulated in Microsoft Excel, and graphical representations were generated using GraphPad Prism 8 (San Diego, CA, USA).

## Results

### Articles identified

Search queries of MEDLINE® Ovid, Embase Ovid and Scopus generated 3,757 non-duplicate articles for consideration. 3,102 articles were excluded during the title and abstract screening, and an additional 472 articles were excluded during the full-text screening. Screening excluded:

- duplicate studies not identified by Covidence;
- studies in languages other than English;
- non-full text original research articles; studies that were irrelevant to the topic of IVD repair;
- studies that used acellular biomaterials not intended for cell delivery, carrier-free cell delivery systems and non-injectable tissue engineered constructs.

**Fig. 1.**
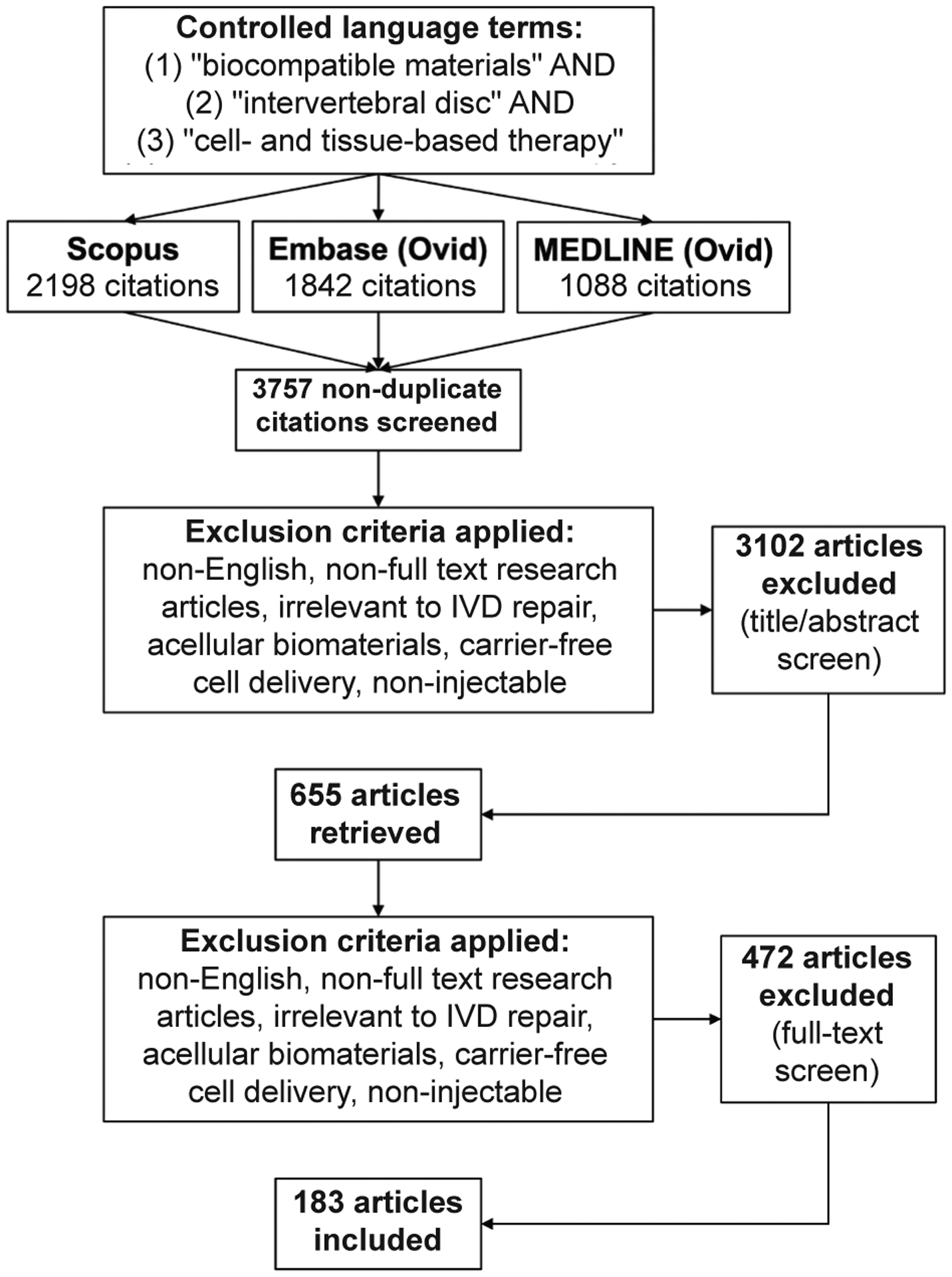
Preferred Reporting Items for Systematic Reviews and Meta-Analyses (PRISMA) Diagram depicting literature search, screening process and exclusion criteria. Search criteria included controlled language terms and keywords related to “biocompatible materials” AND “intervertebral disc” AND “cell- and tissue-based therapy”. The full search query for MEDLINE Ovid and Embase Ovid is available in Appendix A. 183 articles were included in this systematic review from 2000 to 2020.

This resulted in 183 articles that met the inclusion criteria, which were analysed by 1 of 4 reviewers (Fig. 1).

### Target tissue

**Fig. 2.**
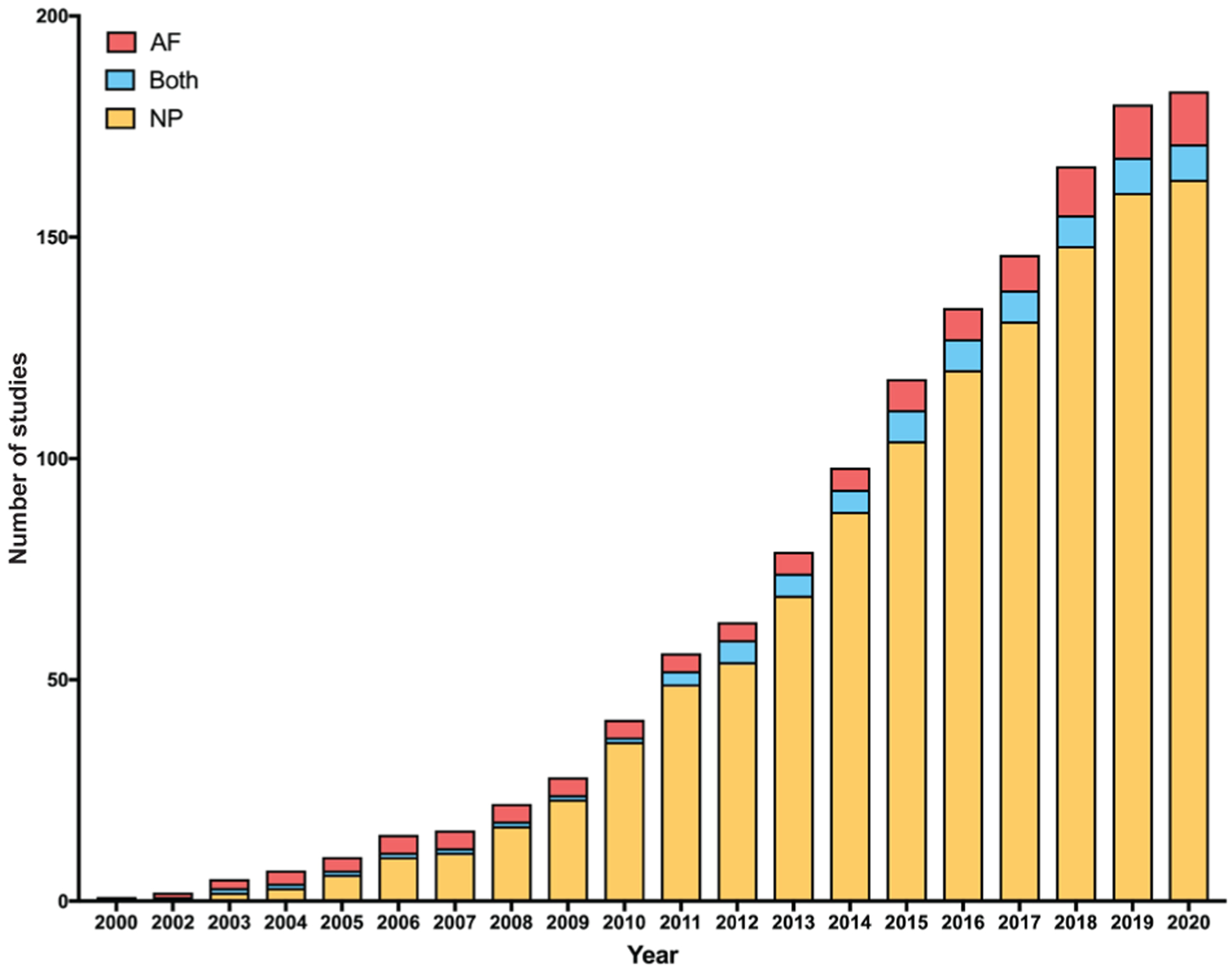
Histogram showing the cumulative number of intervertebral disc (IVD) cell delivery-focused articles as a function of time. For each bar, the red portion represents studies targeting the annulus fibrosus (AF), the yellow portion represents studies targeting the nucleus pulposus (NP) and the blue portion represents studies targeting Both IVD regions.

A sharp increase, over the last two decades, in studies investigating cell delivery biomaterials for IVD repair was observed (Fig. 2). This increase was largely driven by studies that targeted the NP. Of 183 studies, 163 were focused on the NP, while 12 targeted the AF and 8 targeted both IVD regions (Both). The frequency of published articles studying NP cell delivery biomaterials began to increase by 2010. Between 2000 and 2009, 23 articles studying NP cell delivery biomaterials were published and this number approximately doubled to 54 between 2009 and 2012. This rate of article publication persisted through 2019, with an average of 13 NP cell delivery biomaterial studies published per year. Studies targeting the AF or Both IVD regions were published less frequently. The maximum number of AF or Both IVD region cell delivery biomaterial studies published in a given year was 2. Furthermore, between 2000 and 2020, there were 12 years where no published articles targeted the AF, and 16 years where no published studies targeted Both IVD regions.

### Trends in cellular inputs

**Fig. 3.**
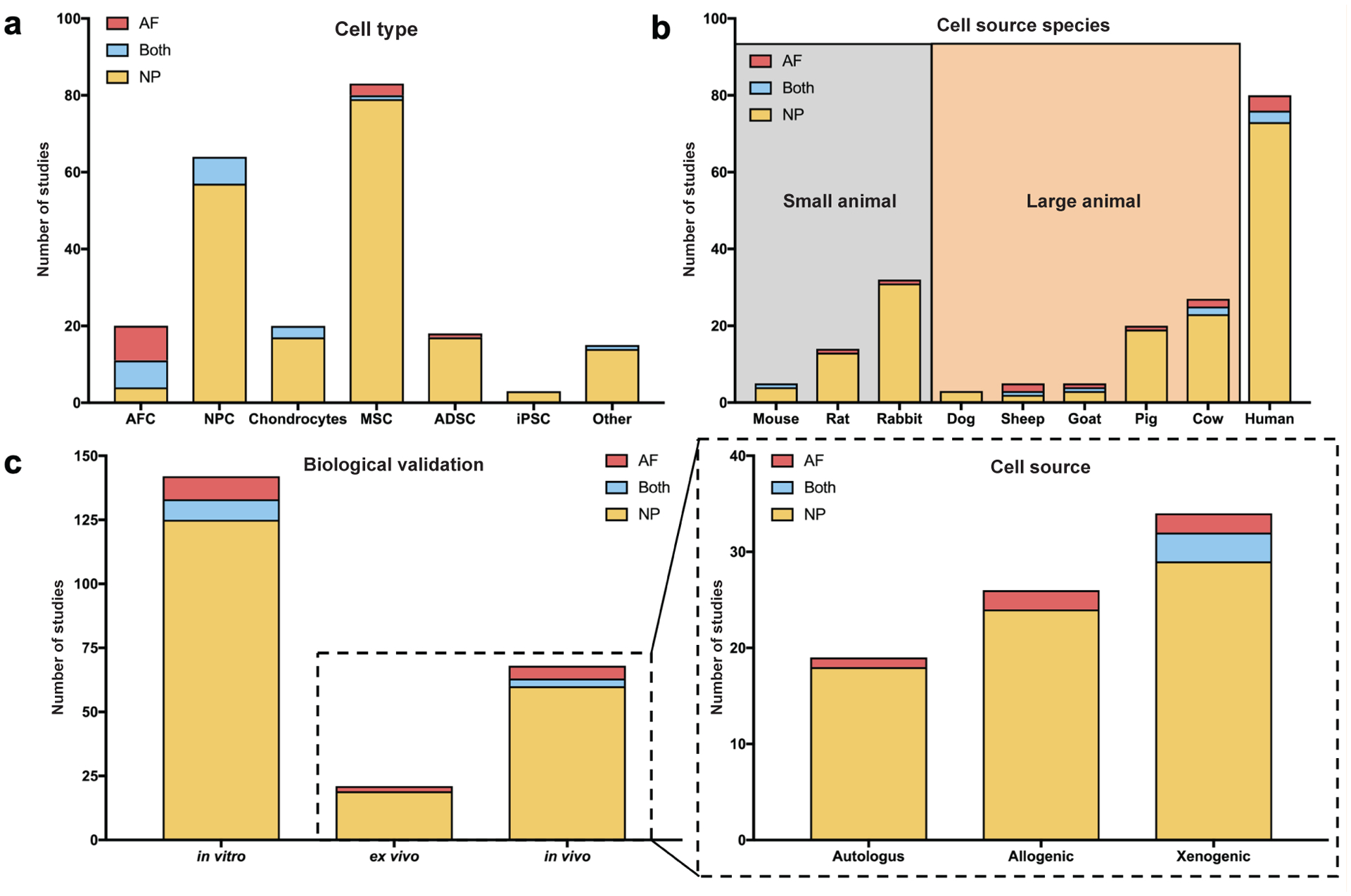
Summary of cellular inputs for IVD cell delivery biomaterial studies. (**a**) Histogram showing the frequency of studies using different cell types for IVD cell delivery. Annulus fibrosus cell (AFC), nucleus pulposus cell (NPC), mesenchymal stem cell (MSC), adipose-derived stem cell (ADSC), included pluripotent stem cell (iPSC). (**b**) Histogram showing the frequency of studies using cells from different animal sources. (**c**) Histogram showing the frequency of studies using different methods of biological validation. For studies that delivered cells in *ex vivo* or *in vivo* model systems, the frequency of particular delivery modes was quantified. For each bar, the red portion represents studies targeting the AF, the yellow portion represents studies targeting the NP and the blue portion represents studies targeting Both IVD regions.

Several different cell types have been proposed to target regeneration of the different areas of the IVD (Fig. 3a). For articles targeting the NP for cell delivery, mesenchymal stem cells (MSCs) (80/163 = 49.1 %) and NP cells (NPCs) (57/163 = 35.0 %) were most commonly used, followed by chondrocytes (18/163 = 11.0 %) and adipose-derived stem cells (ADSC) (17/163 = 10.4 %). Of the 12 studies that targeted the AF, the most common cell types used were AF cells (AFCs) (9/12 = 75.0 %) and MSCs (3/12 = 25.0 %). When attempting to repair Both IVD regions, NPCs (7/8 = 87.5 %) and AFCs (7/8 = 87.5 %) were used most often. Cell types listed as ‘Other’ mostly included non-translatable cell sources for biomaterial cytotoxicity tests (*e.g.* L929 fibroblasts, HeLa cells and HEK-293 cells); one study investigated the delivery of an NP progenitor cell source.

A wide range of species have been used for cell isolation in IVD cell delivery varying from small animals (such as mice, rats and rabbits), larger animals (like goats, pigs and cows), and human tissues (Fig. 3b). For NP repair, human cells were the most commonly tested, (73/163 = 44.8 %), followed by rabbit (31/163 = 19.0 %) and larger animals such as cow (23/163 = 14.1 %) and pig (19/163 = 11.7 %). Cells for AF cell delivery have been obtained from across many species with a slight preference towards human (4/12 = 33.3 %) and cells from large animals, such as sheep (2/12 = 16.7 %) and cow (2/12 = 16.7 %). This pattern was similar for studies that targeted Both IVD regions. Of the studies which used human cells, 25 used IVD cells and 84.0 % (21/25) of those studies used IVD cells harvested from degenerated IVDs.

The biological performance of the IVD cell delivery biomaterials tested in these articles was validated *in vitro*, *ex vivo* and *in vivo* (Fig. 3c) and many studies used multiple models of biological validation to evaluate their IVD cell delivery biomaterial strategies. For NP-targeted studies, most used *in vitro* biological outcomes (126/163 = 77.3 %) and many used *in vivo* validation (60/163 = 36.8 %). A similar trend was observed for AF cell delivery biomaterial strategies (9/12 = 75.0 % and 5/12 = 41.7 %, for *in vitro* and *in vivo* validation, respectively) and strategies targeting Both IVD regions (8/11 = 72.3 % and 3/11 = 27.3 % for *in vitro* and *in vivo* validation, respectively). It was least common for IVD cell delivery biomaterials to be validated biologically using an *ex vivo* model for all target regions; there were no *ex vivo* evaluations in articles characterising cell delivery biomaterials for Both IVD regions. Looking more deeply at the studies that implanted cells using *ex vivo* and *in vivo* model systems, articles investigating NP or AF cell delivery biomaterials used autologous, allogenic and xenogenic study designs. On the contrary, articles targeting Both IVD regions exclusively used a xenogenic approach.

### Trends in biomaterial inputs

**Fig. 4.**
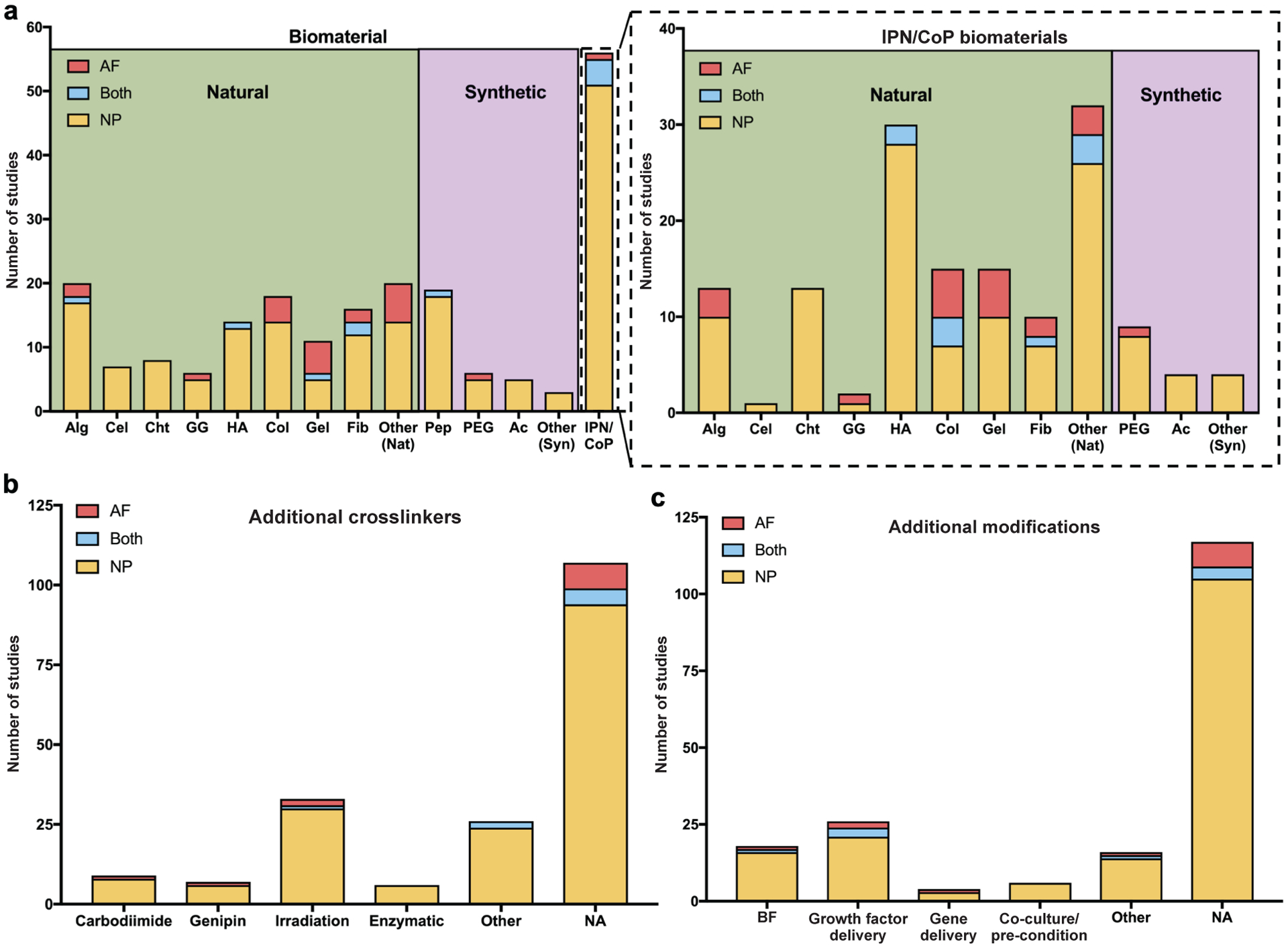
Summary of biomaterial inputs for IVD cell delivery biomaterial studies. (**a**) Histogram showing the frequency of studies using different biomaterial carriers. For studies that used interpenetrating network/co-polymeric (IPN/CoP) biomaterial strategies, the frequency of individual biomaterial components was quantified. Alginate (Alg), cellulose (Cel), chitosan (Cht), gellan gum (GG), hyaluronic acid (HA), collagen (Col), gelatine (Gel), fibrin (Fib), natural (Nat), peptide (Pep), polyethylene glycol (PEG), acrylates (Ac), synthetic (Syn) and interpenetrating network/co-polymeric (IPN/CoP). (**b**) Histogram showing the frequency of studies using various additional crosslinking agents in biomaterial carriers. (**c**) Histogram showing the frequency of studies using various additional modifications to cell-biomaterial delivery systems. Biological functionalisation (BF). For each bar, the red portion represents studies targeting the AF, the yellow portion represents studies targeting the NP and the blue portion represents studies targeting Both IVD regions.

A summary of the macromer components of the biomaterials being evaluated in the 183 reviewed studies identified that there is no consensus on biomaterials used to deliver cells to the AF, NP or Both (Fig. 4a). The general trend shows that a majority of the studies used single network, naturally derived biomaterials. The most commonly used single network biomaterials investigated for NP cell delivery were alginate (17/163 = 10.4 %), collagen (14/163 = 8.6 %), hyaluronic acid (13/163 = 8.0 %) and fibrin (12/163 = 7.4 %). Gelatine (5/12 = 41.7 %) and collagen (4/12 = 33.3 %) were the most commonly used single network biomaterials for AF cell delivery. Studies investigating biomaterials targeting Both IVD regions were the most variable, utilising fibrin (2/8 = 25.0 %), alginate (1/8 = 12.5 %), hyaluronic acid (1/8 = 12.5 %), gelatine (1/8 = 12.5 %) and peptide hydrogels (1/8 = 12.5 %).

The greatest number of articles focused on NP cell delivery utilised interpenetrating network (IPN) or co-polymeric (CoP) biomaterials (51/163 = 31.3 %), defined for this study as biomaterials containing 2 or more types of interlaced polymers. Similar to the single network cell delivery biomaterials, IPN/CoP biomaterials were mostly composed of naturally derived biomaterials. Of the studies investigating IPN/CoP biomaterials, hyaluronic acid (28/51 = 54.9 %), chitosan (13/51 = 25.5 %), collagen (7/51 = 13.7 %) and gelatine (10/51 = 19.6 %) were the most frequently used components. ‘Other’ biomaterials were also very common components of IPN/CoP biomaterials (26/51 = 51.0 %), which included decellularised matrix, chondroitin sulphate and polysaccharides. IPN/CoP biomaterials were much less frequently used in studies targeting the AF for cell delivery, with 1 study investigating a collagen/alginate biomaterial. Studies investigating biomaterials targeting Both IVD regions had the greatest percentage of IPN/CoP biomaterial carriers (4/8 = 50.0 %); hyaluronic acid (2/4 = 50.0 %) and collagen (3/4 = 75.0 %) were the most common components of these biomaterials.

Additional crosslinking mechanisms used to enhance the biomechanical properties of the investigated biomaterials were also summarised. This was considered a crucial piece of the analysis because IVD cell delivery biomaterials will experience high mechanical demands due to the load-bearing function of the IVD, and crosslinking is a simple method of increasing biomechanical modulus and strength of a biomaterial. Interestingly, most of the reviewed studies did not use any additional crosslinking strategies, beyond those crosslinking steps essential for hydrogel formation (107/183 = 58.5 %) (Fig. 4b). Of the studies that utilised additional crosslinking mechanisms, irradiation-based crosslinking was the most common for NP-targeted (30/69 = 43.4 %) and AF-targeted (2/4 = 50.0 %) cell delivery strategies. Studies investigating biomaterials targeting Both IVD regions used irradiation (1/3 = 33.3 %) or ‘Other’ (2/3 = 66.7 %) crosslinking mechanisms.

Lastly, additional modifications to the biomaterial carrier in order to enhance delivered cell function were summarised, and it was found that most studies did not investigate any additional means of enhancing the biological repair capacity of their delivery strategy (119/183 = 65.0 %) (Fig. 4c). Of the studies that made additional modifications to their IVD cell delivery biomaterials, the most popular modification was the incorporation of growth factors into biomaterials targeting the NP (24/57 = 42.1 %), AF (2/4 = 50.0 %) and Both (3/4 = 75.0 %). For NP-targeted biomaterials, biological functionalisation was also common (18/57 = 31.6 %).

### Trends in biological and biomechanical evaluations

**Fig. 5.**
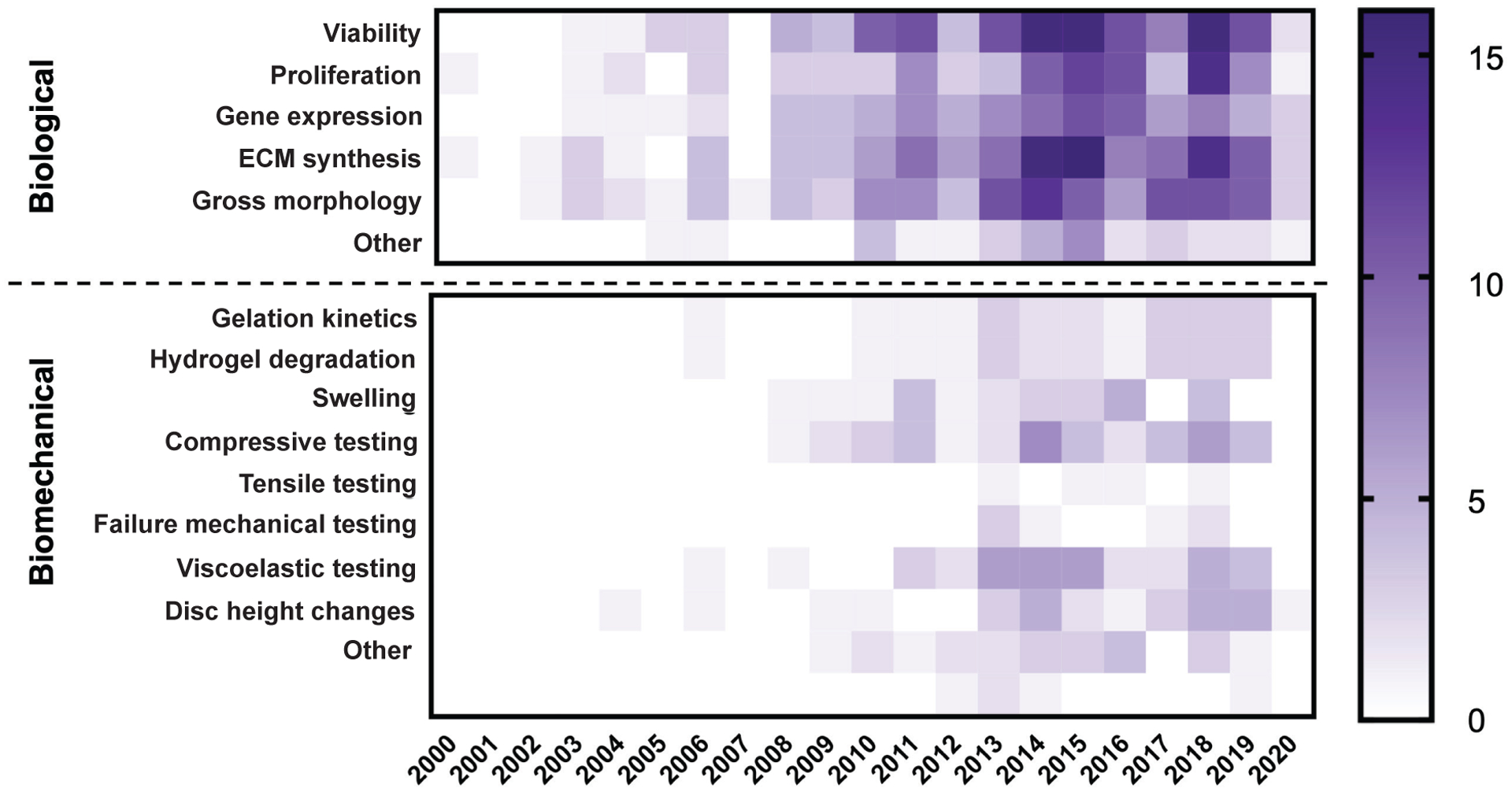
Summary of biological and biomechanical outcome measurements for IVD cell delivery biomaterial studies. Heatmap showing the frequency of various biological and biomechanical outcome measurements as a function of time. Colour intensity of a cell relates to the number of studies published in that year which measure a particular outcome.

The biological and biomechanical outcome measurements used to characterise IVD cell delivery strategies were summarised and evaluated in the 183 reviewed studies. As a whole, it was found that all articles measured at least one biological outcome (*i.e.* viability, proliferation, gene expression, ECM synthesis, gross morphology and ‘Other’) (Fig. 5). For NP-targeted strategies, cell viability was most commonly measured (127/163 = 77.9 %) and most articles also measured ECM synthesis (108/163 = 66.3 %), gross morphology (99/163 = 60.7 %), gene expression (83/163 = 50.9 %) and proliferation (80/163 = 49.0 %). Fewer AF-targeted studies measured viability (3/12 = 25.0 %) and most assessed the success of the repair strategy using gross morphology (10/12 = 83.3 %) and ECM synthesis (9/12 = 75.0 %). A similar trend was observed in studies investigating biomaterials targeting Both IVD regions; whereby, few studies measured viability (1/8 = 12.5 %) and most measured ECM synthesis (5/8 = 62.5 %), gross morphology (4/8 = 50 %) and proliferation (4/8 = 50 %).

Biomechanical outcomes were much less frequently measured (*i.e.* gelation kinetics, hydrogel degradation, swelling, compressive testing, viscoelastic testing, tensile testing, failure mechanical testing, disc height changes and ‘Other’). Out of all the NP-targeted studies, compression testing (35/163 = 21.5 %) and viscoelastic testing (34/163 = 20.9 %) were most frequently evaluated. Compression testing (3/12 = 25 %, 1/8 = 12.5 %) and viscoelastic testing (2/12 = 16.7 %, 1/8 = 12.5 %) were also most common for studies targeting the AF and studies targeting Both IVD regions. For AF-targeted studies, hydrogel degradation (2/12 = 16.7 %), swelling (2/12 = 16.7 %) and disc height changes (2/12 = 16.7 %) were measured at a similar frequency. In studies investigating biomaterials targeting Both IVD regions, gelation kinetics (1/8 = 12.5 %), hydrogel degradation (1/8 = 12.5 %) and swelling (1/8 = 12.5 %) were measured at a similar frequency to compressive and viscoelastic testing. Given the high mechanical demands experienced by the IVD, biomechanical assessments of cell delivery biomaterial strategies were considered to be essential to ensure the biomaterial does not herniate. Furthermore, there was an interest in how the biomechanical properties of tested IVD cell delivery biomaterials compare to their target tissue and what biomaterial inputs led to these properties. For this reason, the next set of analyses focused on studies that reported biomaterial moduli from compressive and viscoelastic testing and summarised their findings to elucidate themes of effective IVD cell delivery biomaterials.

### General themes in biomechanically effective IVD cell delivery biomaterial strategies

**Fig. 6.**
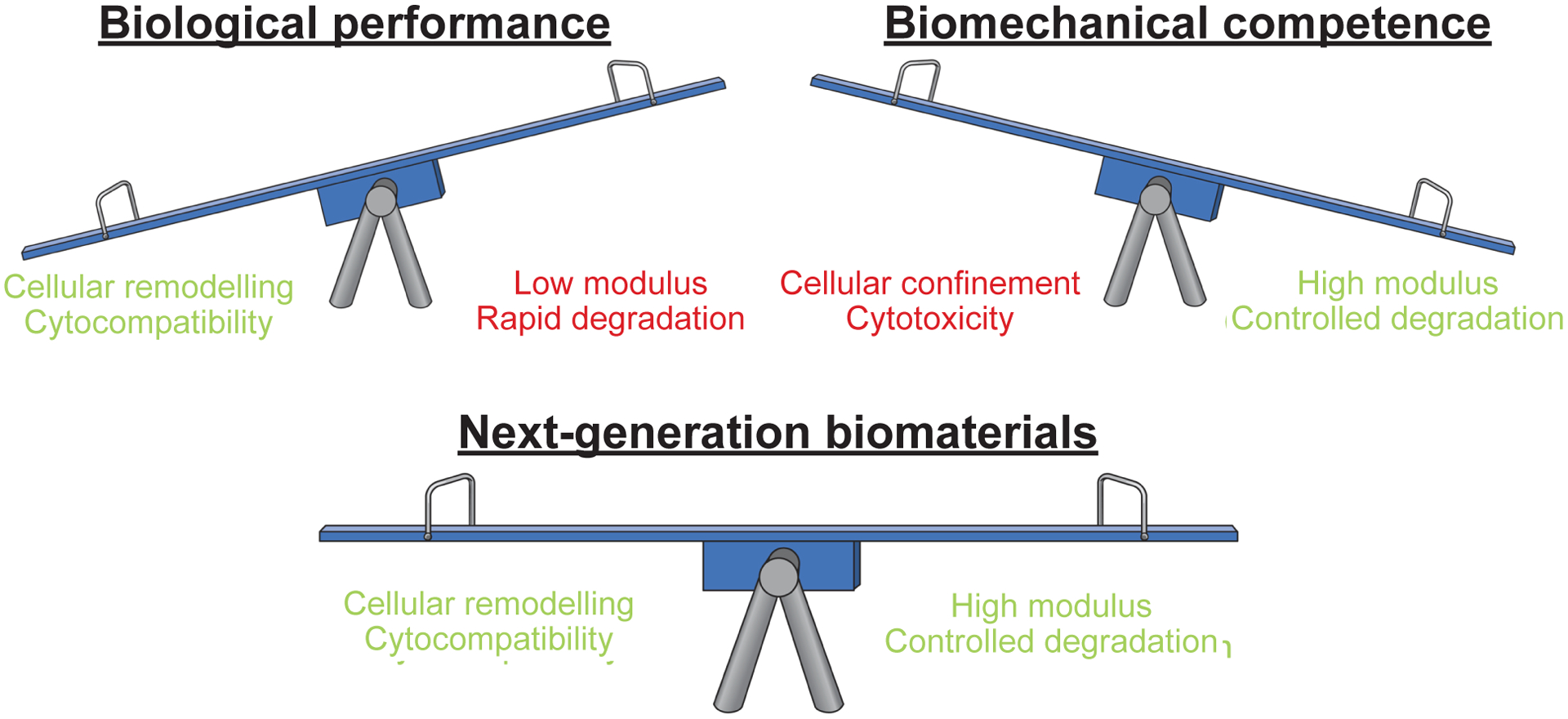
Illustration of the ‘seesaw’ effect, found in numerous studies that reported biomaterial modulus; whereby, formulations with higher moduli had inferior cellular performance, and vice versa. By this effect, biomaterial carriers could be designed for biomechanical competence or biological performance. Next-generation biomaterials designed to achieve both biomechanical competence and biological performance are an area warranting further development.

Approximately one third (58/183 = 31.7 %) of the studies included in this systematic review reported compressive or shear moduli of the IVD cell delivery biomaterial *in vitro*. Through investigation of these 58 studies, 2 common themes were found. The first theme to emerge was a competition between biological performance and biomechanical competence, or ‘seesaw’ effect, found in 43.1 % (25/58) of studies that reported *in vitro* compressive and shear moduli (Fig. 6). In this subset of studies (Table 1), groups evaluated how biological and biomechanical outcomes varied when modifying the concentration of macromer, additional crosslinker or an additional modification used to enhance biomaterial modulus. In 72.0 % (18/25) of these papers, marked in green, it was found that to achieve the greatest moduli, the concentration of macromer, additional crosslinker or additional modification was increased to the point of promoting less survival or ECM synthesis of encapsulated cells. Therefore, there is a balance, or ‘seesaw’ between biological performance and biomechanical competence. Studies which avoided this phenomenon, marked in yellow, (7/25 = 28%) generally reported lower moduli that did not match or just approached the biomechanical properties of the intended target tissue. No currently studied biomaterials are able to match AF material properties with high cell viability and ECM elaboration.

A second theme to emerge was *in vitro* construct maturation, or an increase in moduli values of the construct throughout the culture duration period due to ECM elaboration. Constructs matured in 22.4 % (13/58) of the studies that reported compressive or shear moduli for their material (Table 2). In this subset of studies, groups evaluated how the biomechanical properties of cell-laden biomaterials changed over time. Most of these studies (10/13 = 76.9 %), marked in green, demonstrated that the moduli of cell-laden biomaterial constructs increased throughout culture; the median increase in modulus was approximately two-fold. 23.1 % (3/13) of these studies, marked in yellow, reported that the moduli of cell-laden biomaterial constructs were constant over time, while the moduli of control acellular constructs decreased. Only one of these studies (1/13 = 7.7 %), marked in red, reported a decline in the moduli of cell-laden biomaterial constructs.

A small subset of studies which reported *in vitro* compressive and shear moduli demonstrated both a ‘seesaw’ effect and construct maturation (7/58 = 12.1 %) (Table 3). In these studies, the competition between biomechanical competence and biological performance was recognised, then constructs with high biological performance were matured. In 71.4 % (5/7) of these studies, marked in green, groups experienced a ‘seesaw’ effect, then showed that formulations most conducive to biological function experienced significant increases in moduli throughout culture. These increases were not seen in formulations that had greater initial moduli, closer to the target tissue. The remaining 28.6 % (2/7) of studies, marked in yellow, experienced a ‘seesaw’ phenomenon and showed that the constructs with low initial moduli were biologically favourable since ECM was produced and moduli remained constant over time, while the moduli of acellular constructs with initially high moduli produced little ECM and moduli significantly decreased with culture time.

## Discussion

Research investigating injectable cell delivery biomaterials for IVD repair started as a field approximately 20 years ago and has since grown rapidly to include more than 180 published studies. In the first study identified from 2000, Stern *et al*. investigated a fibrin/hyaluronic acid matrix to enhance proliferation and ECM elaboration of porcine NPCs; overall, few biological assessments were performed and no biomechanical outcomes were reported (Stern *et al*., 2000). 20 years later, sophisticated cellular and biomaterial strategies are being applied to enhance the biological and biomechanical performance of IVD cell delivery biomaterials intended for the NP, AF or Both IVD regions. For example, Hu *et al*. used a thermosensitive hydrogel to deliver growth differentiation factor 5 (GDF5)-transfected human induced pluripotent stem cells (iPSCs) to restore disc height index in a rat caudal IVD injury model (Hu *et al*., 2020). RGD peptide sites were incorporated into polysaccharide cell delivery hydrogels by Wang *et al*. to enhance encapsulated cell function (Wang *et al*., 2019). Alinejad *et al*. and Piluso *et al.* used additional crosslinking agents and starch nanocrystals, respectively, to enhance the mechanical properties of their cell delivery biomaterials, while maintaining encapsulated cell function (Alinejad *et al*., 2019; Piluso *et al*., 2019). All listed studies evaluated their proposed strategies using a variety of biological and biomechanical outcome measurements. These rapid scientific advancements over the past 2 decades are likely to accelerate as the field continues to grow, and it is expected that more intricate IVD cell delivery biomaterial strategies will be developed with greater chances of clinical success.

Most IVD cell delivery studies target the NP for biomaterial-aided cell delivery. This is expected, considering that injectable hydrogels have a highly hydrated amorphous structure more similar to the NP than the AF (Goins *et al*., 2005). There were notably fewer studies investigating the AF as a target for cell delivery using an injectable biomaterial carrier, likely because the fibre-reinforced nature of AF tissue and need to resist tensile stresses is difficult to achieve with injectable hydrogels. AF repair strategies have more often involved devices like the X-Close (Bailey *et al*., 2013) and Barricaid (Parker *et al*., 2016) systems that are designed to close AF defects and prevent reherniation. Additionally, fibre-reinforced tissue engineered constructs to plug AF defects (Sato *et al*., 2003) or recapitulate the organised collagen structure of the AF (Bhattacharjee *et al*., 2012; Nerurkar *et al*., 2007; Park *et al*., 2012) have also been investigated for AF repair, yet they are not injectable. Injectable cell delivery strategies for AF repair and regeneration are compelling because reherniation and recurrent pain remains an unmet clinical need, and because injectable formulations may be applied rapidly during discectomy, or other minimally invasive procedures.

This systematic review shows little consensus on the cell type and cell species used in current IVD cell delivery biomaterial strategies. NPCs and AFCs were very common for studies targeting the NP and AF, respectively. These cell sources are useful for phenotypic characterisation of how IVD cells will behave in experimental biomaterials; however, the low cellularity of aged human IVD tissues (Maroudas *et al*., 1975) and difficulty in surgically harvesting IVD tissue makes use of autologous IVD cells impractical for clinical translation with current technologies. Stem cell sources have greater availability and possess the ability to proliferate and differentiate into a wide range of lineages (Armstrong *et al*., 2012). Of the variety of stem cell sources, MSCs were most popular for both NP- and AF-targeted studies, although ASCs and iPSCs were also used. MSCs are easily accessible, patient-specific and have the capacity to differentiate into various mesodermal cell types, such as NPCs and AFCs (Richardson *et al*., 2010; Richardson *et al*., 2016). Wang *et al*. was the only study to investigate the use of NP-specific progenitor cells for IVD cell delivery (Wang *et al*., 2019). IVD-derived stem/progenitor cells were first identified as a promising cell source in 2007 (Risbud *et al*., 2007); however, more biological characterisation is required before these cells can be more widely adopted (Hu *et al*., 2018). The high frequency by which various stem cell sources are being used in IVD cell delivery biomaterial studies enhances our knowledge and increases their likelihood for clinical translation.

Cells used in reviewed studies were predominantly derived from humans, which highlights a great focus on clinical translatability. Rabbit, cow, pig and rat were the next most commonly used animal cell types. Cells used from different species are likely selected because they are derived from accepted preclinical models of IVD degeneration and repair; thus, enabling more rapid progression from *in vitro* to *in vivo* testing (Daly *et al*., 2016). The rat and rabbit are in the small animal range with benefits of being somewhat more cost-effective, and retaining anatomical features including facet joints, paravertebral muscles and ligaments similar to the human spine (Kroeber *et al*., 2002; Silberberg *et al*., 1979). Cow and pig are commonly used large animal models that can be relatively easily acquired from abattoirs, enabling high force application and nutrient transport distances that more closely simulate loading and nutrient transport challenges of the human IVD (Alini *et al*., 2008; Beckstein *et al*., 2008; Daly *et al*., 2016). This review clarified the lack of consensus on animal models for IVD research; different species and model systems may be used to evaluate specific scientific design goals in each study.

No clear consensus was found on optimal IVD cell delivery biomaterial carriers. The most commonly tested biomaterials were IPN/CoP biomaterials, which combine multiple polymer networks to form materials that are biologically and biomechanically tuneable (Ullah *et al*., 2015; Vedadghavami *et al*., 2017). Natural biomaterials were a common component of these IPN/CoP biomaterials, likely because they are composed of repeating subunits commonly metabolised by humans and they contain binding motifs which mimic natural ECM to support the function of encapsulated cells (Parisi *et al*., 2018). Additional crosslinking, beyond that required to solidify the hydrogel, was employed to increase the biomechanical properties of low modulus materials (Oryan *et al*., 2018). Additional modifications, such as the incorporation of nanoparticles into the biomaterial matrix were also used to increase the moduli of biomaterials with initially low moduli that had favourable biological performance (Merino *et al*., 2015). Conversely, biological functionalisation, incorporation of growth factors/gene vectors, and pre-conditioning/co-culturing delivered cells were strategies to enhance the biological repair capacity of high modulus biomaterials. Overall, a minority of studies used additional crosslinking agents and additional modifications for IVD cell delivery biomaterials, suggesting these strategies require further research before clinical implementation.

All studies reported biological outcomes at either the molecular, cellular or macroscopic hierarchical scale; however, biomechanical outcomes were less commonly reported and mostly evaluated *in vitro*. Molecular and cellular assessments (*i.e.* viability, proliferation and gene expression) are important preliminary benchmarks for an IVD repair strategy, but macroscopic assessments (*i.e.* ECM synthesis and gross morphology) more rigorously demonstrate that the biomaterial provides a supportive niche to promote cell-mediated healing. Molecular, cellular and macroscopic biological outcomes were reported, at a similar high frequency over time, indicating that biological efficacy of IVD cell delivery biomaterials is being appropriately challenged on multiple scales and model systems. Conversely, biomechanical outcome measurements were only reported in approximately 60 % of the reviewed studies and the biomechanical outcome measurements most commonly reported were *in vitro* compressive and viscoelastic testing. Tensile testing was rarely performed, which is perhaps not surprising given the injectable biomaterials reviewed have low tensile moduli; however, it does demonstrate an unmet challenge given the tensile demands of the IVD under loading (Jacobs *et al*., 2013; Nerurkar *et al*., 2010; Tsantrizos *et al*., 2005). These *in vitro* biomechanical tests determine if the biomaterial properties mimic the target tissue; however, *in situ* biomechanical testing (*e.g. ex vivo* or *in vivo* failure testing) is required to assess the herniation risk and functional biomechanical restoration of a potential IVD cell delivery biomaterial. Such *in situ* biomechanical outcome measurements were reported much less frequently, highlighting the need to evaluate the herniation risk and functional performance of IVD cell delivery biomaterial strategies at multiple hierarchical scales. *Ex vivo* evaluations, using bioreactors that recapitulate the IVD physiological environment, are a useful benchmark in this hierarchy because they can rigorously assess the biological and biomechanical performance of a particular strategy in a highly controlled manner (Gantenbein *et al*., 2015; Pfannkuche *et al*., 2020). Testing paradigms to screen IVD repair biomaterials at progressive hierarchical scales exist (Long *et al*., 2016; Virk *et al*., 2020), which can be adapted to screen IVD cell delivery biomaterial strategies by including molecular, cellular and macroscopic biological assessments of cells within candidate biomaterials *in vitro, ex vivo* and *in vivo*. Assessing biological and biomechanical outcome measurements at these various hierarchical scales and model systems will best challenge the IVD cell delivery biomaterial strategy, allow for targeted modifications and more rapidly advance to clinical translation.

Balancing biomechanical and biological performance remains an ubiquitous challenge when developing repair strategies for the IVD and other musculoskeletal soft-tissues that experience high mechanical demands (D’Este *et al*., 2018). Studies that reported compressive and shear moduli to elucidate themes of effective IVD cell delivery biomaterials were, therefore, investigated. One important theme that stood out was the ‘seesaw’ effect where, in most studies that reported biological and biomechanical outcomes for a range of biomaterial formulations, the biomaterial formulations with the greatest moduli had the poorest biological performance of encapsulated cells (Table 1). For example, Francisco *et al*. demonstrated that increasing polyethylene glycol macromer concentrations from 5 % to 6 % reduced NPC survival but increased complex shear modulus (Francisco *et al*., 2014). Zhou *et al*. reported that increasing genipin additional crosslinker concentration from 0.01 % to 1 % w/v decreased viability and NPC differentiation capacity, but increased Young’s modulus (Zhou *et al*., 2018a). Park *et al*. found that incorporating higher concentrations of silk into their biomaterial reduced chondrogenic gene expression and ECM synthesis while increasing Young’s modulus (Park *et al*., 2012). Several mechanisms can explain this ‘seesaw’ phenomenon. First, crosslinking reagents that are somewhat benign at low concentrations, can become cytotoxic to cells at high concentrations (Cao *et al*., 2012; Panebianco *et al*., 2020; Saito *et al*., 2008); thus, the amount of crosslinker required for a biomaterial to achieve the modulus of a load-bearing tissue like the IVD could be too high to simultaneously achieve cytocompatibility, motivating need for innovation. Second, increasing the concentration of macromers and additional crosslinkers reduces biomaterial porosity (Brown and Barker, 2014; Cruise *et al*., 1998), which can limit cell functionality by inhibiting nutrient transport, preventing cell-biomaterial interactions and physically constraining cells (Fan and Wang, 2017). Lastly, substrate stiffness, which is implicitly modified through the concentration of macromers, additional crosslinkers and additional modifications, can negatively influence cell behaviours (Fearing *et al*., 2018; Gilchrist *et al*., 2011; Jiang *et al*., 2016). The few studies that avoided the ‘seesaw’ effect reported lower moduli and these biomaterials would not likely provide initial biomechanical stabilisation upon injection. Moreover, the increase in macromer and additional crosslinker concentrations required to achieve appropriate moduli was reported to be detrimental to the function of encapsulated cells.

A second theme that emerged was construct maturation where constructs exhibited enhanced biomechanical function with culture duration (Tables 2 & 3). The subset of studies showing substantial construct maturation used natural biomaterials which are biodegradable; thus, the mechanical properties of these materials are expected to decrease over time. The modulus of most cultured biomaterials increased over time, suggesting that the rate of ECM synthesis was more rapid than the biomaterial degradation rate. This was demonstrated for alginate (Chou and Nicoll, 2009), cellulose (Varma *et al*., 2018), gellan gum (Silva-Correia *et al*., 2013), hyaluronic acid (Kim *et al*., 2015), peptide (Barreto-Henriksson *et al*., 2019) and decellularised NP matrix (Mercuri *et al*., 2013) hydrogels. The biomaterials presented in these studies are highly promising for translation because if they have sufficient initial adhesive and biomechanical properties to remain within the IVD space upon implantation, then their *in vitro* performance suggests they can promote cells to repair and regenerate the IVD ECM *ex vivo* and *in vivo*. However, this requires further investigation in appropriate preclinical models before clinical translation. The few studies that did not report an increase in modulus with culture generally demonstrated that their cell-laden constructs had no decline in modulus, while the modulus of acellular control constructs decreased over time (Kumar *et al*., 2014; Reza and Nicoll, 2010a; Reza and Nicoll, 2010b). In general, the rate of change of modulus values are attributed to the rate of ECM synthesis relative to the rate of biomaterial degradation. The hydrogels in green with excellent biomechanical maturation increased less than an order of magnitude, so it is likely that biomechanical properties must start in the range of native tissue or mature over an extensive period of time to approach native tissue material properties. The median increase in biomaterial moduli was greater for studies that had relatively low macromer, additional crosslinker or additional modification concentrations in order to prioritise biological performance; therefore, the initial moduli of the injectable biomaterial started relatively low offering little initial biomechanical competence. Overall, this subset of studies highlights the importance of biomaterial degradation as a parameter for cell delivery biomaterial strategies (Kong *et al*., 2004). At this time, it remains a challenge to develop high modulus biomaterial carriers that can encapsulate delivered cells and promote substantial ECM synthesis.

This systematic review of IVD cell delivery biomaterials had the following limitations. First, studies that only evaluated the performance of cells seeded on top of experimental biomaterials were excluded because two-dimensional cell-biomaterial interactions fail to sufficiently evaluate how cells will behave after injection within a three-dimensional biomaterial carrier (Cukierman *et al*., 2001; Pampaloni *et al*., 2007). Similarly, biomechanical data from previously published acellular studies on biomaterials were not included in this review because they were not characterised as cell carriers in those studies, and it was difficult to determine whether formulations changed subtly between acellular and cell-seeded studies. This systematic review was able to determine which cell types and biomaterials were most commonly used for IVD cell delivery strategies, but this method could not identify which strategies are best because frequent use does not imply superiority. Applying this logic would inaccurately select for more established strategies and bias against novel strategies. The current analysis also could not determine a single best strategy, because not all studies reported the same outcome measurements, and those which did often used different assays that were not directly comparable. However, this systematic review was able to highlight important themes for IVD cell delivery biomaterial strategies that were biologically and biomechanically effective. This knowledge is expected to help guide future injectable IVD cell delivery biomaterial strategies and accelerate their clinical translation to reduce the burden of chronic discogenic back pain.

## Conclusions

This systematic review highlights the rapidly increasing numbers of studies investigating injectable IVD cell delivery biomaterials. Most studies focused on delivering cells to the NP region, with fewer studies on injectable AF cell delivery biomaterials. There was no consensus on ideal cell type or biomaterial carrier choice; however, MSCs and natural biomaterials (*e.g.* alginate, collagen, hyaluronic acid and fibrin) were most commonly investigated in the reviewed studies. Since the IVD is a load-bearing tissue, the biological and biomechanical function of experimental cell delivery biomaterials were summarised, yet only a subset of studies reported biomechanical outcomes. Most of these studies only reported *in vitro* compressive and viscoelastic testing of the hydrogel without reporting *in situ* biomechanical performance (*e.g. ex vivo* or *in vivo* failure testing), which is necessary to fully assess a strategy’s herniation risk and reparative capacity. When analysing the subset of IVD cell delivery biomaterial studies that reported compressive and shear moduli, 2 themes were identified. First was a ‘seesaw’ effect, whereby biomechanical competence competed with the biological performance of a given biomaterial strategy. Second was the maturation of low-modulus biomaterial constructs by ECM synthesis that outpaces biomaterial degradation. A clear opportunity still exists for next-generation injectable IVD cell delivery biomaterial strategies that can provide initial biomechanical competence while promoting sufficient biological performance to enable construct maturation for long-term tissue healing.

## Supplementary Material

Appendix B

## Figures and Tables

**Table 1.** Studies reporting biomaterial moduli for range of formulations. Studies marked in green experienced a ‘seesaw’ effect; whereby, to achieve the greatest moduli, the concentration of macromer, additional crosslinker or additional modification was increased to the point of promoting less survival or ECM synthesis of encapsulated cells. Studies marked in yellow varied the concentration of a component in the IVD cell delivery biomaterial system, but did not experience this phenomenon. Black and coloured arrows indicate changes in the inputs and outputs for the IVD cell delivery biomaterial system, respectively. ↑ indicates an increase in the concentration of the specified component in the IVD cell delivery biomaterial system. + indicates the inclusion of the specified component in the IVD cell delivery biomaterial system. ↑, ↔ or ↓ indicate that the resultant biological or biomechanical output measured increased, remained the same, or decreased, respectively, as a result of the change in the IVD cell delivery biomaterial system (*e.g.* “↑ Genipin conc., ↓, NP gene expression” means that as genipin concentration increased, the NP gene expression decreased). G′, G″ and G* values are reported at 1 Hz. (Table 1 on next page.)

study	Macromer	Crosslinker	Additional modifications	Culture duration	Primary biological findings	Primary biomechanical findings
**np studies**						
Chou *et al*., 2009	Alg [2 – 3 %]	Irradiation (Irgacure 2959) [0.05 %]	NA	**2 weeks**	↑ Methacrylation ↓ Viability	E_y_: ~0.6 – 9 kPa; ↑ Methacrylation and Alg conc. ↑ E_y_
Growney Kalaf *et al*., 2016	Alg [1 % w/v]	Other (CaCO_3_ & GDL) [15 – 75 & 30 – 150 mmol/L]	NA	**<2 h**	↑ CaCO_3_:GDL ratio ↓ Viability	G′ = ~295 – 2100 Pa, G″ = ~19 – 1300 Pa; ↑ CaCO_3_:GDL ratio ↑ G′ and G″
Zhou *et al*., 2018a	Col [3 mg/mL]	Genipin [0 – 1 % w/v]	NA	**2 weeks**	↑ Genipin conc. ↓ NP gene expression ↓ NP ECM synthesis	E_y_: ~1 – 20 MPa (AFM); ↑ Genipin conc. ↑ E_y_
Khang *et al*., 2015	GG [0.75 % – 2 % w/v]	NA	NA	**2 weeks**	↑ HA-GG conc. ↑ Viability	G′: ~20 – 60 kPa; ↑ HA-GG conc. ↓ G′
Pereira *et al*., 2011	GG [0.75 – 2 % w/v]	NA	Other (GG MPs)	**2 weeks**	↑ LAGG MP conc. ↔ Cell viability	G′: ~20 – 80 kPa; ↑ LAGG MP conc. ↑ G′
Moss *et al*., 2011	HA [1.5 % w/v]	Other (PEGDA) [0.8 %]	Other (Elastin-like peptide)	**3 weeks**	+ Elastin-like peptide ↔ Cell viability	H_A_: ~17 – 31 kPa; + Elastin-like peptide ↑ HA
Toh *et al*., 2012	HA [2 % w/v]	Enzymatic (HRP & H_2_O_2_) [0.125 U/mL & 500 – 1000 μmol/L]	NA	**3 weeks**	↑ H_2_O_2_ conc. ↓ Chondrogenic ECM synthesis	E_y_: ~5 – 12 kPa ↑ H_2_O_2_ conc. ↑ E_y_
Francisco *et al*., 2014	PEG [2 – 10 % w/v]	Irradiation (Irgacure 2959) [0.1 % w/v]	BF (Laminin-111) [0 – 1000 μg/mL]	**1 week**	↑ PEG conc. ↓ Cell viability	G*: ~0.4 – 20 kPa; ↑ PEG conc. ↑ G*
Ligorio *et al*., 2019	Pep [10 – 20 mg/mL]	NA	Other (Graphene oxide) [0 – 0.5 mg/mL]	**1 week**	+ Graphene oxide ↔ Cell viability	G′: ~4 – 16 kPa; + Graphene oxide ↑ G′
Tao *et al*., 2015	Pep [1 %]	NA	BF (BMP-7 functional peptides)	**4 weeks**	+ RAD-KPS ↑ Proliferation ↑ ECM synthesis	G′: ~100 – 200 Pa, G″: ~20 – 40 Pa; + RAD - KPS ↓ G″ and G′
Wang *et al*., 2014	Pep [1 % w/v]	NA	BF (Link-N functional peptides)	**2 weeks**	+ Link-N functional peptide ↑ Chondrogenic gene expression	G′: ~5 – 6 kPa, G″: ~0.8 – 0.9 kPa; + Link-N functional peptide ↔ G′ and G″
Alinejad *et al*., 2018	Cht & CS [2 % & 0 – 1 % w/v]	Other (BGP & SHC) [0.1 – 0.4 & 0.075 mol/L]	NA	**1 week**	↑ CS conc. ↑ Metabolic activity (1 week)	G′: ~5 – 20 kPa; ↑ CS conc. ↓ G′
Alinejad *et al*., 2019	Cht [2 % w/v]	Other (BGP & SHC) [0.1 – 0.4 & 0.075 mol/L]	NA	**2 weeks**	↑ SHC/BGP conc. ↑ Viability and metabolic activity	E_y_: ~2 – 8 kPa, G′: ~1 – 8 kPa, G″ ↑ SHC/BGP conc. ↑ E_y_ and G′
Calderon *et al*., 2010	Col & HA [5 & 1.57 mg/mL]	Carbodiimide (EDC/NHS) [8 – 48 mmol/L]	NA	**3 weeks**	↑ EDC/NHS conc. ↓ DNA content ↓ NP gene expression	E_y_: ~1 – 8 kPa; ↑ EDC/NHS conc. ↑ E_y_
Frith *et al*., 2013	HA & PEG [25 & 100 mg/mL]	Enzymatic (HRP & H_2_0_2_) [0.25 U/mL & 1 – 5 μmol/L]	BF (PPS) [5 μg/mL]	**3 weeks**	+ PPS ↑ Chondrogenic ECM synthesis	G′: ~1 – 5 kPa, G″: ~1 – 10 Pa; + PPS ↓ G′
Frith *et al*., 2014	HA & PEG [15 & 16.5 mg/mL]	Enzymatic (HRP & H_2_O_2_) [0.25 U/mL & 1 – 5 μmol/L]	BF (PPS) [5 – 20 μg/mL]	**3 weeks**	↑ PPS ↑ ECM synthesis	G′: ~1 – 5 kPa, G″: ~0.2 – 100 Pa; ↑ HA - PPS or PPS ↔ G′ or G″
Gan *et al*., 2017	Gel, Dex & PEG [10 wt% total, 1:1.1:8 of Dex/Gel:PEG]	Irradiation (Irgacure 2959) [0.5 % w/v]	NA	**4 weeks**	↑ PEG:Dex/Gel ratio ↑ Viability and proliferation ↑ ECM synthesis	E_y_: ~2 – 60 kPa, G*: ~12 – 46 kPa; ↑ PEG : Dex/Gel ratio ↓ E_y_ and G*
Li *et al*., 2014	Fib & HA [6 – 13 & 0.7 – 1.3 mg/mL]	NA	NA	**2 weeks**	↑ Fib:HA ratio ↑ NP gene expression	G′: ~80 – 350 Pa ↑ Fib : HA ratio ↓ G′
Park *et al*., 2011	Fib & HA [10 & 10 mg/mL]	NA	Other (Silk) [1–2 %]	**4 weeks**	↑ Silk conc. ↓ Chondrogenic gene expression ↓ ECM synthesis	E_y_: ~3 – 10 kPa; ↑ Silk conc. ↑ E_y_
Peroglio *et al*., 2012	HA & Pep [0.5 % w/v]	NA	NA	**1 week**	↑ Pep molecular weight ↓ Viability	G′: ~0.005 – 16 kPa; ↑ Pep molecular weight ↑ G′
Zhou *et al*., 2018b	Col & CS [3 & 8.1 mg/mL]	Genipin [0.01 – 1 % w/v]	NA	**2 weeks**	↑ Genipin conc. ↓ Viability	G′: ~2400 Pa, G″: 230 Pa; ↑ Genipin conc. ↑ G′ and G″
Zhu *et al*., 2017	Cht & HA [0.2–1.2 % & 0.1 – 0.6 %)	Other (BGP) [3 %]	Growth Factor Delivery (Kartogenin)	**26 d**	↑ Cht conc. No biological evaluation	E_y_: ~0.9 – 2.9 MPa ↑ Cht conc. ↑ E_y_
**Af studies**						
Cruz *et al*., 2018	Fib [35 – 140 mg/mL]	Genipin [1 – 6 mg/mL]	NA	**7 weeks**	↑ Genipin conc. ↓ Viability ↓ GAG synthesis	E_y_: ~30 – 200 kPa, G*: ~7 – 65 kPa; ↑ Fib and Genipin conc. ↑ E_y_ and G*
Pereira *et al*., 2018	GG [0.75 – 1 wt%]	NA	Other (Cel nanocrystals) [1.25 – 2.5 wt%]	**2 weeks**	↑ GGMA/Cel nanocrystal conc. ↓ Viability	E_y_: ~45 – 55 kPa, G*: ~0.009 – 0.6 kPa; ↑ GGMA/Cel nanocrystal conc. ↑ G*
**Both studies**						
Piluso *et al*., 2019	Gel [5 % w/v]	Irradiation (LAP) [0.3 % w/v]	Other (Starch nanocrystals) [0 – 0.5 wt%]	**1 week**	↑ Starch nanocrystal conc. ↔ Cell viability	E_y_: ~1.5 – 3 kPa; ↑ Starch nanocrystal conc. ↑ E_y_

**Table 2.** Studies reporting biomaterial moduli of constructs cultured over time. Studies marked in green, yellow and red demonstrated that the moduli of cell-laden biomaterial constructs increased, remained constant, or decreased, respectively, throughout the specified culture duration. Coloured arrows indicate changes in the outputs for the IVD cell delivery biomaterial system over culture time. ↑, ↔ or ↓ indicate that the resultant biological or biomechanical output measured increased, remained the same, or decreased, throughout culture. G′, G″ and G* values are reported at 1 Hz.

study	Macromer	Additional crosslinker	Additional modifications	Culture duration	Primary biological findings	Culture	Primary biomechanical findings
Chou *et al*., 2009	Alg [2 %]	Irradiation (Irgacure 2959) [0.05 %]	NA	**4 weeks**	↑ Proteoglycan synthesis	**8 weeks**	↑ E_y_: ~1.3 – 4.3 kPa (~200 %)
Foss *et al*., 2014	Alg [0.5 – 4 %]	NA	Other (GCSN & CS) [125 – 500 & 100 – 400 mg/mL]	**4 weeks**	↑ Cell viability and proliferation ↑ Col II synthesis ↓ GAG and water content	**4 weeks**	↑ H_A_: ~0.2 – 1.4 MPa (~100 %)
Reza *et al*., 2010a	Cel [1 – 5 %]	Irradiation (Irgacure 2959) [0.05 %]	NA	**1 week** **2 weeks**	↑ Cell viability ↑ ECM synthesis	**2 weeks**	→ E_y_: ~2 – 5 kPa (N.S. change)
Reza *et al*., 2010b	Cel [2.5 %]	Irradiation (Irgacure 2959) [0.05 %]	NA	**4 weeks**	↑ DNA content ↑ ECM synthesis	**4 weeks**	→ E_y_: ~5 – 20 kPa (D0 N.R.)
Gupta *et al*., 2011	Cel [2 %]	Irradiation (Irgacure 2959) [0.05 %]	NA	**3 weeks**	↑ Proliferation ↑ Col II, CS and GAG synthesis	**3 weeks**	↑ E_y_: ~1 – 2 kPa (~25 %)
Gupta *et al*., 2014	Cel [1.75 %]	Irradiation (Irgacure 2959) [0.05 %]	NA	**8 weeks**	↑ ECM synthesis	**8 weeks**	↑ E_y_: ~2 – 30 kPa (~900 %)
Varma *et al*., 2018	Cel [2 %]	APS/TEMED [10 mM & 10 mM]	NA	**5 weeks**	↑ Cell viability ↑ ECM synthesis	**5 weeks**	↑ E_y_: ~5 – 30 kPa (~500 %)
Silva-Correia *et al*., 2013	GG [2 % w/v]	Irradiation (Methyl benzoylformate) [0.1 % w/v]	NA	**3 weeks**	↑ Cell viability	**3 weeks**	↑ G′: ~60 – 120 kPa (~50 %)
Kim *et al*., 2015	HA [1 % w/v]	Irradiation (Irgacure 2959) [0.5 % w/v]	NA	**8 weeks**	↑ Cell viability ↑ NP-specific gene expression ↑ ECM synthesis	**8 weeks**	↑ E_y_: ~2 – 160 kPa (~8000 %) ↑ G*: ~1 – 2 MPa (D0 N.R.)
Kumar *et al*., 2014	Ac [13.5 % w/v]	Irradiation (Irgacure 2959) [0.5 % w/v]	NA	**2 weeks**	↑ Cell viability and proliferation ↑ ECM synthesis	**2 weeks**	↔ E_y_: ~2.5 – 4.5 kPa (N.S. change)
Barreto-Henriksson *et al*., 2019	Pep [1 %]	NA	NA	**3 weeks**	↑ GAG synthesis	**4 weeks**	↑ G′: ~82 – 138 kPa (~70 %) ↑ G″: ~16 – 25 kPa (~55 %)
Wan *et al*., 2016	Pep [25 – 35 mg/mL]	NA	NA	**2 weeks**	↑ Cell viability ↑ ECM synthesis	**2 weeks**	↓ G′: ~5 – 20 kPa (~75 %) *↔* G″: ~1 – 3 kPa (N.S change)
Mercuri *et al*., 2013	Decellularised porcine NP matrix	Carbodiimide (EDC/NHS & PGG) [30 mmol/L/6 mmol/L & 0.15 %]	NA	**2 weeks**	↑ Cell viability ↑ ECM synthesis	**2 weeks**	↑ G*: ~4 – 20 kPa (~100 %)

**Table 3.** Studies reporting biomaterial moduli for a range of formulations and constructs cultured over time. Studies marked in green and yellow demonstrated that the moduli of cell-laden biomaterial constructs increased or remained constant, respectively, throughout the specified culture duration. Black and coloured arrows indicate changes in the inputs and outputs for the IVD cell delivery biomaterial system, respectively. ↑ and ↓ indicates an increase or decrease, respectively, in the concentration of the specified component in the IVD cell delivery biomaterial system. ↑, ↔ or ↓ indicate that the resultant biological or biomechanical output measured increased, remained the same, or decreased, respectively, as a result of the change in the IVD cell delivery biomaterial system or throughout the specified culture time. G′, G″ and G* values are reported at 1 Hz.

study	Macromer	Crosslinker	Culture duration	Primary biological findings	Culture duration	Primary biomechanical findings
Gupta *et al*., 2015	Cel [1.5 – 3.5 %]	Irradiation (Irgacure 2959) [0.05 %]	**1 week** **6 weeks**	1.5 % Cel hydrogel ↑ Acan and Col II gene expression ↑ GAGs and Col II synthesis	**6 weeks**	↑ E_y_: ~2 – 11 kPa (~130 %) of 1.5 % Cel hydrogel ↓ E_y_ of 3.5 % Cel hydrogel
Lin *et al*., 2016	Cel [2 – 4 %]	Irradiation (Irgacure 2959) [0.05 %]	**4 weeks**	↓ CMC conc. ↑ ECM synthesis	**4 weeks**	↓ CMC conc. ↑ E_y_: ~2 – 11 kPa (~130 %)
Halloran *et al*., 2008	Col, HA & Acn [5, 0.55 & 1 mg/mL]	Enzymatic (mTGase) [0.05 mg/mL]	**1 week**	+ HA, Acn and mTGase *↔* Cell viability ↑ sGAG retention	**1 weeks**	+ mTGase ↑ G′: ~500 – 1250 Pa ↑ E_y_ (No value, reported as sig. increased)
Hayami *et al*., 2011	PCL & Cht [40 – 100 & 10 – 60 % vol%]	Irradiation (Irgacure 2959) [0.1 % w/v]	**2 weeks**	30 vol % xMGC-9.3KELAST scaffold ↑ Proliferation ↑ Metabolic activity ↑ ECM synthesis	**2 weeks**	↑ Elastomer concentration ↓ Equilibrium modulus 30 vol% xMGC - 9.3KELAST ↑ Equilibrium modulus: ~100 – 2100 kPa (~500 %)
Hayami *et al*., 2013	PCL & Cht [25 – 30 % w/v & 6 – 8 %]	Irradiation (Irgacure 2959) [0.1 % w/v]	**1 week** **8 weeks**	↑ Elastomer conc. *↔* Cell viability 7030TMCCL ↑ DNA, GAG and collagen	**8 weeks**	↑ Elastomer conc. ↓ Equilibrium modulus 7030TMCCL ↑ Equilibrium modulus: ~600 – 1500 kPa (~10 %)
Hayami *et al*., 2016	HA, Cht & CS [6 %, 6 % & 20 % w/v]	Irradiation (Irgacure 2959) [0.1 % w/v]	**5 weeks**	MGC - MCS & MHA - MCS blends ↑ Total DNA ↑ ECM synthesis	**5 weeks**	MGC-MCS & MHA-MCS blends ↑ Equilibrium Modulus: ~100 – 275 kPa (~30 %)
Navaro *et al*., 2015	Tetronic1307 & Fib [Tetronic 1307 : Fib, 1 : 4]	Irradiation (Irgacure 2959) [0.1 % w/v]	**2 weeks**	1 kPa Matrix ↑ Proliferation and chondrogenic differentiation 2 kPa Matrix ↑ Osteogenic differentiation	**2 weeks**	1 kPa matrix ↑ G′: ~0.2 – 0.6 kPa (~200 %) 2 kPa matrix ↔ G′

## Discussion with Reviewers

Reviewer 1: Which are the most promising cell delivery materials you suggest to further investigate and why?

Authors: The most promising IVD cell delivery biomaterials in this review are those which have demonstrated construct maturation in Tables 2 and 3. Assuming they have sufficient initial adhesive and biomechanical properties to remain within the IVD space, the fact that they have demonstrated sufficient ECM synthesis to enhance construct modulus is very promising for their ability to promote IVD repair and regeneration *ex vivo* and *in vivo*. However, this requires further investigation in appropriate preclinical models before clinical translation.

Reviewer 1: What are the main advantages and limitations of the models you reported?

Authors: This systematic review highlighted a need to measure multiple biological and biomechanical outputs at varying hierarchical scales. Most IVD cell delivery biomaterial strategies investigated only tested *in vitro*, which does not simulate the clinical repair environment. Assessing biological and biomechanical outcome measurements at various hierarchical scales will best challenge the IVD cell delivery biomaterial strategy, allow for targeted modifications and more rapidly advance to clinical translation.

Andrea Vernengo: Although crosslinking is clearly the most common way to improve mechanical properties, three articles cited in the paper (Merino *et al*., 2015; Pereira *et al*., 2018; Piluso *et al*., 2019) report increases in the mechanical properties of hydrogels with addition of micro or nanoparticles. In the latter two citations, cell response was not adversely affected by addition of the particles. Another study reported good cell viability with increased mechanical reinforcement by microparticles in ionically crosslinked gellan gum (Pereira *et al*., 2011). Could the authors offer some comments on two-phase composites, which appear to have potential in terms of balancing mechanical properties and cell performance?

Authors: Our analysis of articles which reported biomaterial moduli for a range of formulations (Table 1) showed that composite biomaterial strategies (*i.e.* strategies listed as “Other” under the “Additional Modifications” heading) generally avoided the ‘seesaw’ effect, which could make these types of strategies promising for balancing the biological and biomechanical demands of an IVD cell delivery biomaterial.

Andrea Vernengo: While softer hydrogels support higher cell viability and ECM expression, have they been shown to support AF phenotypes? Did the analysis reveal any relationships between hydrogel crosslinking and the expression of NP or AF related markers, at the gene or protein level?

Authors: There is evidence to show that increasing matrix stiffness downregulates NP marker gene expression (Additional reference: Fearing *et al*., 2018). Our analysis showed that there was a decline in the expression of IVD ECM genes when increasing the concentration of macromer, additional crosslinker or an additional modification. This could be a direct consequence of matrix stiffness or due to crosslinker cytotoxicity and reduced biomaterial porosity.

## Appendix A

### MEDLINE Ovid and Embase Ovid search queries

1. exp biocompatible materials/
2. exp hydrogels/
3. exp gels/
4. exp tissue scaffolds/
5. exp polymers/
6. exp microspheres/
7. biomaterial*.mp.
8. scaffold*.mp.
9. hydrogel*.mp.
10. gel*.mp.
11. polymer*.mp.
12. biocompatible.mp.
13. bead*.mp.
14. sphere*.mp.
15. 1 or 2 or 3 or 4 or 5 or 7 or 8 or 9 or 10 or 11 or 12 or 13 or 14
16. exp intervertebral disc/
17. exp intervertebral disc degeneration/
18. intervertebral*.mp.
19. nucleus pulposus.mp.
20. annulus fibrosus.mp.
21. 16 or 17 or 18 or 19 or 20
22. exp “cell- and tissue-based therapy”/
23. exp guided tissue regeneration/
24. exp tissue engineering/
25. exp regenerative medicine/
26. regen*.mp.
27. tissue engineering.mp.
28. repair.mp.
29. cell*.mp.
30. therap*.mp.
31. deliver*.mp.
32. seed*.mp.
33. implant*.mp.
34. carr*.mp.
35. vehicle*.mp.
36. laden.mp.
37. 30 or 31 or 32 or 33 or 34 or 35 or 36
38. 29 and 37
39. 22 or 23 or 24 or 25 or 38
40. 15 and 21 and 39

## Table abbreviations

7030TMCCL: poly(ε-caprolactone-co-trimethylene carbonate)
Ac: acrylates
Acn: aggrecan
AFM: atomic force microscopy
Alg: alginate
APS: ammonium persulphate
BGP: β-glycerophosphate
BMP-7: bone morphogenic protein-7
BF: biological functionalisation
Cel: cellulose
Cht: chitosan
Col: collagen
CS: chondroitin sulphate
Dex: dextran
EDC: 1-ethyl-3-(3-dimethylaminopropyl)-carbodiimide
Ey: Young’s compressive modulus
Fib: fibrin
G*: complex shear modulus at 1 Hz
G′: storage modulus at 1 Hz
G″: loss modulus at 1 Hz
GCSN: glucosamine
GDL: glucono-δ-lactone
Gel: gelatine
GG: gellan gum
HA: hyaluronic acid
HRP: horse radish peroxidase
LAP: lithium phenyl(2,4,6-trimethylbenzoyl)-phosphinate
MCS: methacrylated chondroitin sulphate
MGC: methacrylated glycol chitosan
MHA: methacrylated hyaluronic acid
MPs: microparticles
mTGase: microbial transglutamase
NHS: N-hydroxysuccinimide
PCL: polycaprolactone
PEG: polyethylene glycol
PEGDA: polyethylene glycol diacrylate
Pep: peptide
PGG: pentagalloyl glucose
PPS: pentosan polysulphate
PRP: platelet rich plasma
SHC: sodium hydrogen carbonate
TEMED: tetramethylethylenediamine
