# Supplementary material for: BALANCING BIOLOGICAL AND BIOMECHANICAL PERFORMANCE IN INTERVERTEBRAL DISC REPAIR: A SYSTEMATIC REVIEW OF INJECTABLE CELL DELIVERY BIOMATERIALS
Source: Eur Cell Mater. Author manuscript; Available in PMC 2020 Dec 1. (PMC7706585; doi:10.22203/eCM.v040a15)
Supplement: Appendix B [file NIHMS1648198-supplement-Appendix_B.pdf]

European Cells and Materials Vol. 40 2020 (pages 239-258) DOI: 10.22203/eCM.v040a15 ISSN 1473-2262

BALANCING BIOLOGICAL AND BIOMECHANICAL PERFORMANCE IN INTERVERTEBRAL DISC REPAIR: A SYSTEMATIC REVIEW OF INJECTABLE CELL DELIVERY BIOMATERIALS

C.J. Panebianco, J.H. Meyers, J. Gansau, W.W. Hom and J.C. Iatridis*

Leni and Peter W. May Department of Orthopaedics, Icahn School of Medicine at Mount Sinai, New York, NY, USA

Abstract

Discogenic back pain is a common condition without approved intervertebral disc (IVD) repair therapies. Cell delivery using injectable biomaterial carriers offers promise to restore disc height and biomechanical function, while providing a functional niche for delivered cells to repair degenerated tissues. This systematic review advances the injectable IVD cell delivery biomaterials field by characterising its current state and identifying themes of promising strategies. Preferred Reporting Items for Systematic Reviews and Meta-Analyses (PRISMA) guidelines were used to screen the literature and 183 manuscripts met the inclusion criteria. Cellular and biomaterial inputs, and biological and biomechanical outcomes were extracted from each study. Most identified studies targeted nucleus pulposus (NP) repair. No consensus exists on cell type or biomaterial carrier, yet most common strategies used mesenchymal stem cell (MSC) delivery with interpenetrating network/co-polymeric (IPN/CoP) biomaterials composed of natural biomaterials. All studies reported biological outcomes with about half the studies reporting biomechanical outcomes. Since the IVD is a load-bearing tissue, studies reporting compressive and shear moduli were analysed and two major themes were found. First, a competitive balance, or 'seesaw' effect, between biomechanical and biological performance was observed. Formulations with higher moduli had inferior cellular performance, and vice versa. Second, several low-modulus biomaterials had favourable biological performance and matured throughout culture duration with enhanced extracellular matrix synthesis and biomechanical moduli. Findings identify an opportunity to develop next-generation biomaterials that provide high initial biomechanical competence to stabilise and repair damaged IVDs with a capacity to promote cell function for long-term healing.

Keywords: Intervertebral disc, annulus fibrosus, nucleus pulposus, biocompatible materials, biomaterials, hydrogels, tissue engineering, regenerative medicine, cell- and tissue-based therapy, cell delivery.

*__Address for correspondence__: James C. Iatridis, Ph.D., One Gustave Levy Place, Box 1188, Department of Orthopaedics, Icahn School of Medicine at Mount Sinai, New York, NY 10029, USA.
Telephone number: +1 2122411517 Email: james.iatridis@mssm.edu

Copyright policy: This article is distributed in accordance with Creative Commons Attribution Licence (http://creativecommons.org/licenses/by-sa/4.0/).

Introduction

Low back and neck pain are pressing public health concerns, estimated to affect up to 80 % of the adult population during their lifetimes (Hoy *et al.*, 2014; Rubin, 2007). Between 1990 and 2015, the years lived with disability caused by low back pain increased by 54 % globally, making it the leading cause of disability (Vos *et al.*, 2016). In 2016, low back and neck pain ranked highest, amongst 154 high-cost conditions, for healthcare spending in the United States. Costing $ 134.5 billion, more money was spent treating low back and neck pain than other musculoskeletal disorders, diabetes, cardiovascular disease or cancer (Dieleman *et al.*, 2020). Chronic low back pain is a complex condition with multiple contributors, and treatment options have limited efficacy (Hartvigsen *et al.*, 2018). Pathologies of the intervertebral disc (IVD) are strongly associated with back pain, and IVD involvement is estimated in 39 - 42 % of back pain patients, making the IVD a prime therapeutic target (Adams and Dolan, 2012; DePalma *et al.*, 2011; Livshits *et al.*, 2011; Ohtori *et al.*, 2015).

The IVD is a fibrocartilaginous tissue that bears high-magnitude spine loads and permits three-dimensional motions of the spinal column (Shapiro and Risbud, 2014). These functions are made possible by the complex structural features of the IVD. The

outer ring of the IVD is a fibre-reinforced, angle-ply laminate called the annulus fibrosus (AF), which consists of highly organised collagen lamellae. The AF gradually transitions from its outer region – which is primarily composed of type I collagen with elongated, fibroblast-like cells – to the inner region consisting of rounded fibrocartilage cells surrounded by a matrix of type I and II collagen (Eyre and Muir, 1976; Torre *et al*., 2019). The centrally located core of the IVD is the nucleus pulposus (NP); a gelatinous, proteoglycan-rich structure with rounded, chondrocyte-like cells in a randomly-oriented type II collagen network (Pattappa *et al*., 2012; Shapiro and Risbud, 2014). In a healthy state, the highly pressurised NP is contained by the fibrous AF so the IVD may resist large axial loads with high pressurisation and smaller matrix strains (Iatridis *et al*., 2013; Miele *et al*., 2012). With aging and degeneration, IVD hydration, proteoglycan content, and extracellular matrix (ECM) synthesis decrease, while collagen crosslinking and the presence of tears and defects increase (Adams and Roughley, 2006; Antoniou *et al*., 1996; Duance *et al*., 1998; Nerlich *et al*., 1997). Overloading and aging of the IVD can accelerate cell-mediated degeneration of the IVD, resulting in impaired biomechanical function, structural failure, and painful conditions (Adams and Roughley, 2006; Iatridis *et al*., 2013).

Identifying the aetiology of back pain, and distinguishing aging and degeneration, remain unmet clinical challenges. Due to these complexities, current treatment options for patients experiencing discogenic back pain, (*i.e.* back pain where IVD degeneration is the most prominent diagnosis) have limited efficacy. Current guidelines recommend self-management, physical and psychosocial therapies, and some forms of complementary medicine as the first-line treatment option for acute and persistent low back pain. Clinical guidelines for interventional surgery vary (National Guideline Centre (UK), 2016; Qaseem *et al*., 2017; Stochkendahl *et al*., 2018), but if a patient continues to experience persistent pain with radiographic evidence of herniation, spinal stenosis or IVD degeneration, they may be candidates for surgical interventions (Foster *et al*., 2018). However, surgeries are primarily palliative and often fail because they do not target root causes of IVD degeneration. For example, the neuropathy, disability and pain associated with IVD herniation can be treated with a discectomy surgery. Discectomy procedures remove herniated NP tissue from the IVD, but do not seal AF defects caused by the herniation, which can lead to accelerated degeneration, reherniation and recurrent pain (Carragee *et al*., 2003; Gray *et al*., 2006; Lurie *et al*., 2014; Parker *et al*., 2015; Schroeder *et al*., 2012). Spinal fusion and total disc replacement can be effective treatments for late-stage degeneration (*i.e.* Thompson Grade IV/V) when well indicated, but altered biomechanical loading and can lead to degeneration of IVDs adjacent to the spinal fusion site, called adjacent segment disease (Geisler *et al*., 2009; Ghiselli *et al*., 2004; Phillips *et al*., 2013; Siepe *et al*., 2014; Xia *et al*., 2013). The significant drawbacks in current treatments for discogenic back pain and poor intrinsic healing capacity of the IVD highlight a critical need to develop next-generation IVD therapies that are capable of slowing down the progression of early IVD degeneration and promoting repair.

Cell therapy is a promising treatment for progressive IVD degeneration since it has shown efficacy in numerous preclinical and clinical studies (Sakai, 2011; Schol and Sakai, 2019). Exogenous cells can promote ECM synthesis, and secrete paracrine signals that may stimulate endogenous IVD cells to synthesise ECM and release immunomodulatory signals to combat excessive inflammation (Clouet *et al*., 2019). One concern of IVD cell delivery is cell-leakage and off-targeting effects. Vadalà *et al*. demonstrated that injection of mesenchymal stem cells (MSCs) with saline into an *in vivo* rabbit model of IVD degeneration resulted in ectopic osteophyte formation (Vadalà *et al*., 2012). Aligning with the classical tissue engineering paradigm (Langer and Vacanti, 1993), injectable cell delivery biomaterials may enhance cell delivery therapies by retaining injected cells at the injury site and providing an instructive microenvironment that supports cell viability and cell-mediated healing (Burdick *et al*., 2016). Injectable biomaterials have potential for clinical translation because they are minimally invasive, gel *in situ*, and fill irregularly-shaped defects. Many of these biomaterials can also be tuned to mimic the biomechanical properties of their target tissue, which is considered particularly important for repair of the IVD and other musculoskeletal tissues that provide a predominantly mechanical function (Bowles and Setton, 2017; Guterl *et al*., 2013).

Barriers to the clinical adoption of injectable IVD cell delivery repair strategies include lack of a consensus on which cell source and delivery vehicle are optimal for this therapeutic strategy, and a need to prevent reherniation of any injected biomaterial (Benneker *et al*., 2014; Iatridis *et al*., 2013; Smith *et al*., 2018). To help overcome these obstacles, a systematic review of the literature on injectable biomaterials used for cell delivery in IVD repair was conducted. The first goal of this systematic review was to define the state of the field for cell delivery biomaterials designed for IVD repair. Second, this review identified the most commonly reported biological and biomechanical outcome measurements used to assess published strategies. Third, the effect of cell source and biomaterial carrier choice on the biological and biomechanical performance of published IVD repair strategies was determined to identify common themes for successful repair strategies. It is hoped that this summary and synthesis of IVD cell delivery biomaterial literature will help accelerate their clinical translation to reduce the burden of chronic discogenic back pain.

Materials and Methods

Identification of peer-reviewed manuscripts for analysis

Identification and retrieval of literature was conducted using the Preferred Reporting Items for Systematic Reviews and Meta-Analyses (PRISMA) guidelines (Moher *et al.*, 2009). A comprehensive search, using controlled language terms and keywords, was conducted in MEDLINE® Ovid, Embase Ovid and Scopus from the date of database inception through April 9th, 2020. Search criteria, agreed upon before executing the search, were established to include controlled language terms and keywords related to "biocompatible materials" AND "intervertebral disc" AND "cell- and tissue-based therapy". The full search query for MEDLINE Ovid and Embase Ovid is available in Appendix A. Initial results yielded a total of 5,128 articles across all databases. Search results were exported into Covidence software (Melbourne, Australia) for deduplication and screening. Deduplication in Covidence removed 1,371 duplicate articles. Titles and abstracts of the remaining 3,757 articles were screened by 2 independent reviewers using Covidence. If conflicts arose, the full-text manuscript was reviewed to arrive at a consensus. Screening excluded:

- duplicate studies not identified by Covidence;
- studies in languages other than English;
- non-full text original research articles;
- studies that were irrelevant to the topic of intervertebral disc (IVD) repair;
- studies that used acellular biomaterials not intended for cell delivery, carrier-free cell delivery systems and non-injectable tissue engineered constructs.

Based on these inclusion/exclusion criteria, a total of 183 full-length, peer-reviewed manuscripts were identified.

Data extraction and analysis

Before reviewing the identified manuscripts, specific categories for analysis were defined related to the cellular and biomaterial inputs of the IVD repair strategy and the biological and biomechanical outcome measures. Cellular inputs included: target tissue (AF, NP or Both), cell type, cell species and mode of delivery. Biomaterial inputs included: biomaterial composition, method of additional crosslinking and additional modifications to the cell-biomaterial delivery strategy (*i.e.* biological functionalisation, growth factor delivery, gene delivery, co-culture/pre-conditioning, and 'Other'). Biomaterial formulations were referred to using the same language as the original article, for consistency. Concentrations were reported as they were reported in the original article, because not all manuscripts provided sufficient information for unit conversions. Biological functionalisation was defined as the modification of a biomaterial carrier with molecules that promote cell adhesion, proliferation, ECM synthesis, *etc*. Biological outcomes extracted included: validation method (*i.e. in vitro, ex vivo* and/or *in vivo*), viability, proliferation, gene expression, ECM synthesis, and gross morphology (*i.e.* assessment of cell morphology *in vitro* or tissue morphology *ex vivo* or *in vivo*). Biomechanical outcomes extracted included: validation method, gelation kinetics, hydrogel degradation, swelling, compressive testing, viscoelastic testing, tensile testing, failure mechanical testing, and disc height changes.

Aside from the validation method, each category was assigned a "yes/no" binary. If a study was assigned a "yes," details on the specific method of analysis performed and results related to that outcome were noted (*e.g.* assay used to assess viability and the outcome of the viability assessment). If an outcome was noted that did not fall into the categories mentioned, it was assigned to an "Other" category for biological or biomechanical outcomes, and its details were noted. Manuscripts were randomly assigned to a total of four independent reviewers, who read each paper in detail and noted responses for each category. When questions arose (*e.g.* a measure was not clearly defined), the manuscript was reviewed by an additional reader to arrive at a consensus. Data were tabulated in Microsoft Excel, and graphical representations were generated using GraphPad Prism 8 (San Diego, CA, USA).

Results

Articles identified

Search queries of MEDLINE® Ovid, Embase Ovid and Scopus generated 3,757 non-duplicate articles for consideration. 3,102 articles were excluded during the title and abstract screening, and an additional 472 articles were excluded during the full-text screening. Screening excluded:

- duplicate studies not identified by Covidence;
- studies in languages other than English;
- non-full text original research articles; studies that were irrelevant to the topic of IVD repair;
- studies that used acellular biomaterials not intended for cell delivery, carrier-free cell delivery systems and non-injectable tissue engineered constructs.

This resulted in 183 articles that met the inclusion criteria, which were analysed by 1 of 4 reviewers (Fig. 1).

Target tissue

A sharp increase, over the last two decades, in studies investigating cell delivery biomaterials for IVD repair was observed (Fig. 2). This increase was largely driven by studies that targeted the NP. Of 183 studies, 163 were focused on the NP, while 12 targeted the AF and 8 targeted both IVD regions (Both). The frequency of published articles studying NP cell delivery biomaterials began to increase by 2010. Between 2000 and 2009, 23 articles studying

NP cell delivery biomaterials were published and this number approximately doubled to 54 between 2009 and 2012. This rate of article publication persisted through 2019, with an average of 13 NP cell delivery biomaterial studies published per year. Studies targeting the AF or Both IVD regions were published less frequently. The maximum number of AF or Both IVD region cell delivery biomaterial studies published in a given year was 2. Furthermore, between 2000 and 2020, there were 12 years where no published articles targeted the AF, and 16 years where no published studies targeted Both IVD regions.

Trends in cellular inputs

Several different cell types have been proposed to target regeneration of the different areas of the IVD (Fig. 3**a**). For articles targeting the NP for cell delivery, mesenchymal stem cells (MSCs) (80/163 = 49.1 %) and NP cells (NPCs) (57/163 = 35.0 %) were most commonly used, followed by chondrocytes (18/163 = 11.0 %) and adipose-derived stem cells (ADSC) (17/163 = 10.4 %). Of the 12 studies that targeted the AF, the most common cell types used were AF cells (AFCs) (9/12 = 75.0 %) and MSCs (3/12 = 25.0 %). When attempting to repair Both IVD regions, NPCs (7/8 = 87.5 %) and AFCs (7/8 = 87.5 %) were used most often. Cell types listed as 'Other' mostly included non-translatable cell sources for biomaterial cytotoxicity tests (*e.g.* L929 fibroblasts, HeLa cells and HEK-293 cells); one study investigated the delivery of an NP progenitor cell source.

A wide range of species have been used for cell isolation in IVD cell delivery varying from small animals (such as mice, rats and rabbits), larger animals (like goats, pigs and cows), and human tissues (Fig. 3**b**). For NP repair, human cells were the most commonly tested, (73/163 = 44.8 %), followed by rabbit (31/163 = 19.0 %) and larger animals such as cow (23/163 = 14.1 %) and pig (19/163 = 11.7 %). Cells for AF cell delivery have been obtained from across many species with a slight preference towards human (4/12 = 33.3 %) and cells from large animals, such as sheep (2/12 = 16.7 %) and cow (2/12 = 16.7 %). This pattern was similar for studies that targeted Both IVD regions. Of the studies which used human cells, 25 used IVD cells and 84.0 % (21/25) of those studies used IVD cells harvested from degenerated IVDs.

The biological performance of the IVD cell delivery biomaterials tested in these articles was validated *in vitro*, *ex vivo* and *in vivo* (Fig. 3**c**) and many studies used multiple models of biological validation to evaluate their IVD cell delivery biomaterial strategies. For NP-targeted studies, most used *in vitro* biological outcomes (126/163 = 77.3 %) and many used *in vivo* validation (60/163 = 36.8 %). A similar trend was observed for AF cell delivery biomaterial strategies (9/12 = 75.0 % and 5/12 = 41.7 %, for *in vitro* and *in vivo* validation, respectively) and strategies targeting Both IVD regions (8/11 = 72.3 % and 3/11 = 27.3 % for *in vitro* and *in vivo* validation, respectively). It was least common for IVD cell delivery biomaterials to

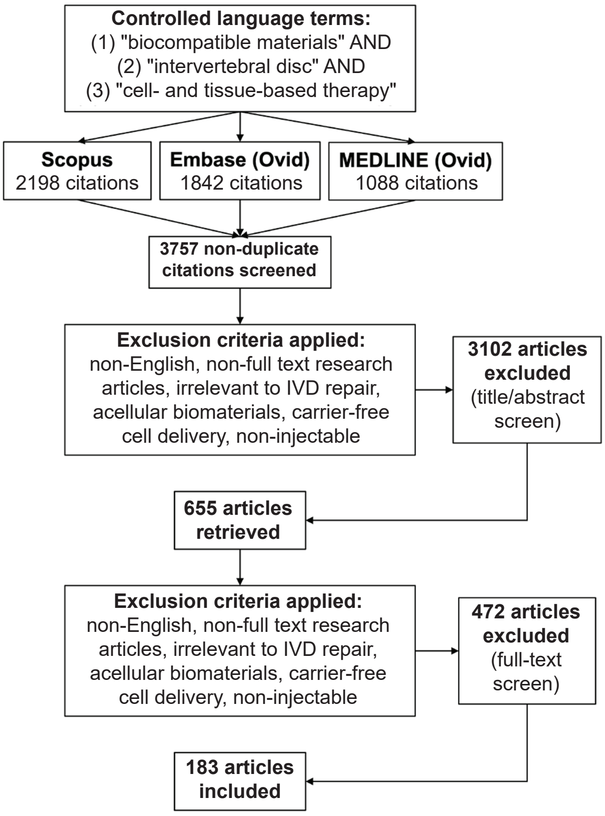

Fig. 1. Preferred Reporting Items for Systematic Reviews and Meta-Analyses (PRISMA) Diagram depicting literature search, screening process and exclusion criteria. Search criteria included controlled language terms and keywords related to "biocompatible materials" AND "intervertebral disc" AND "cell- and tissue-based therapy". The full search query for MEDLINE Ovid and Embase Ovid is available in Appendix A. 183 articles were included in this systematic review from 2000 to 2020.

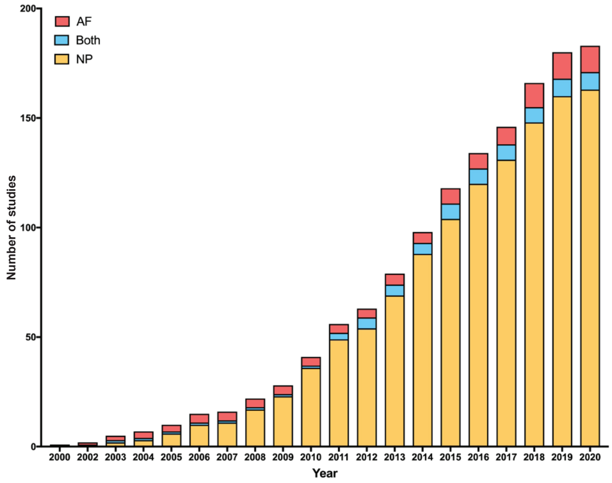

Fig. 2. Histogram showing the cumulative number of intervertebral disc (IVD) cell delivery-focused articles as a function of time. For each bar, the red portion represents studies targeting the annulus fibrosus (AF), the yellow portion represents studies targeting the nucleus pulposus (NP) and the blue portion represents studies targeting Both IVD regions.

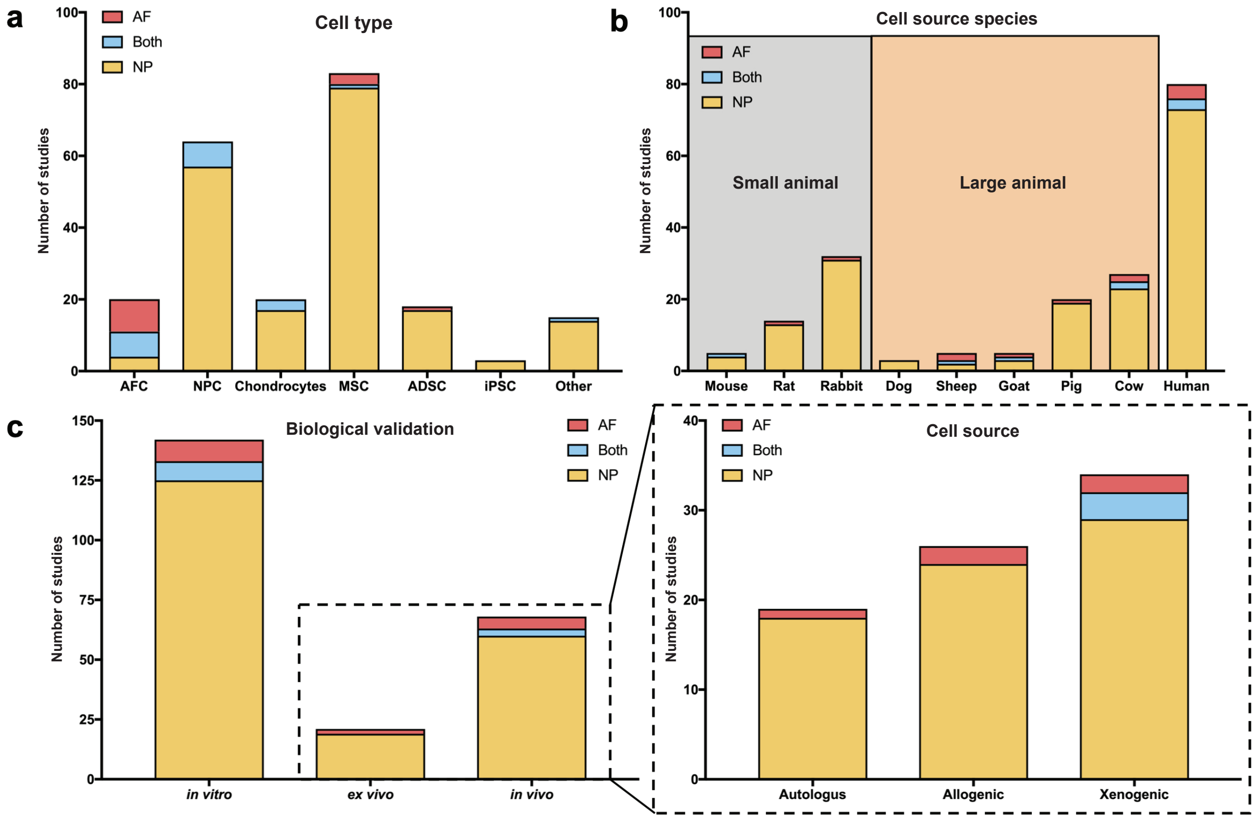

Fig. 3. Summary of cellular inputs for IVD cell delivery biomaterial studies. (**a**) Histogram showing the frequency of studies using different cell types for IVD cell delivery. Annulus fibrosus cell (AFC), nucleus pulposus cell (NPC), mesenchymal stem cell (MSC), adipose-derived stem cell (ADSC), included pluripotent stem cell (iPSC). (**b**) Histogram showing the frequency of studies using cells from different animal sources. (**c**) Histogram showing the frequency of studies using different methods of biological validation. For studies that delivered cells in *ex vivo* or *in vivo* model systems, the frequency of particular delivery modes was quantified. For each bar, the red portion represents studies targeting the AF, the yellow portion represents studies targeting the NP and the blue portion represents studies targeting Both IVD regions.

be validated biologically using an *ex vivo* model for all target regions; there were no *ex vivo* evaluations in articles characterising cell delivery biomaterials for Both IVD regions. Looking more deeply at the studies that implanted cells using *ex vivo* and *in vivo* model systems, articles investigating NP or AF cell delivery biomaterials used autologous, allogenic and xenogenic study designs. On the contrary, articles targeting Both IVD regions exclusively used a xenogenic approach.

Trends in biomaterial inputs

A summary of the macromer components of the biomaterials being evaluated in the 183 reviewed studies identified that there is no consensus on biomaterials used to deliver cells to the AF, NP or Both (Fig. 4**a**). The general trend shows that a majority of the studies used single network, naturally derived biomaterials. The most commonly used single network biomaterials investigated for NP cell delivery were alginate (17/163 = 10.4 %), collagen (14/163 = 8.6 %), hyaluronic acid (13/163 = 8.0 %) and fibrin (12/163 = 7.4 %). Gelatine (5/12 = 41.7 %) and collagen (4/12 = 33.3 %) were the most commonly used single network biomaterials for AF cell delivery. Studies investigating biomaterials targeting Both IVD regions were the most variable, utilising fibrin (2/8 = 25.0 %), alginate (1/8 = 12.5 %), hyaluronic acid (1/8 = 12.5 %), gelatine (1/8 = 12.5 %) and peptide hydrogels (1/8 = 12.5 %).

The greatest number of articles focused on NP cell delivery utilised interpenetrating network (IPN) or co-polymeric (CoP) biomaterials (51/163 = 31.3 %), defined for this study as biomaterials containing 2 or more types of interlaced polymers. Similar to the single network cell delivery biomaterials, IPN/CoP biomaterials were mostly composed of naturally derived biomaterials. Of the studies investigating IPN/CoP biomaterials, hyaluronic acid (28/51 = 54.9 %), chitosan (13/51 = 25.5 %), collagen (7/51 = 13.7 %) and gelatine (10/51 = 19.6 %) were the most frequently used components. 'Other' biomaterials were also very common components of IPN/CoP biomaterials (26/51 = 51.0 %), which included decellularised matrix, chondroitin sulphate and polysaccharides. IPN/CoP biomaterials were much less frequently used in studies targeting the AF for cell delivery, with 1 study investigating a collagen/alginate biomaterial. Studies investigating biomaterials targeting Both IVD regions had the greatest percentage of IPN/CoP

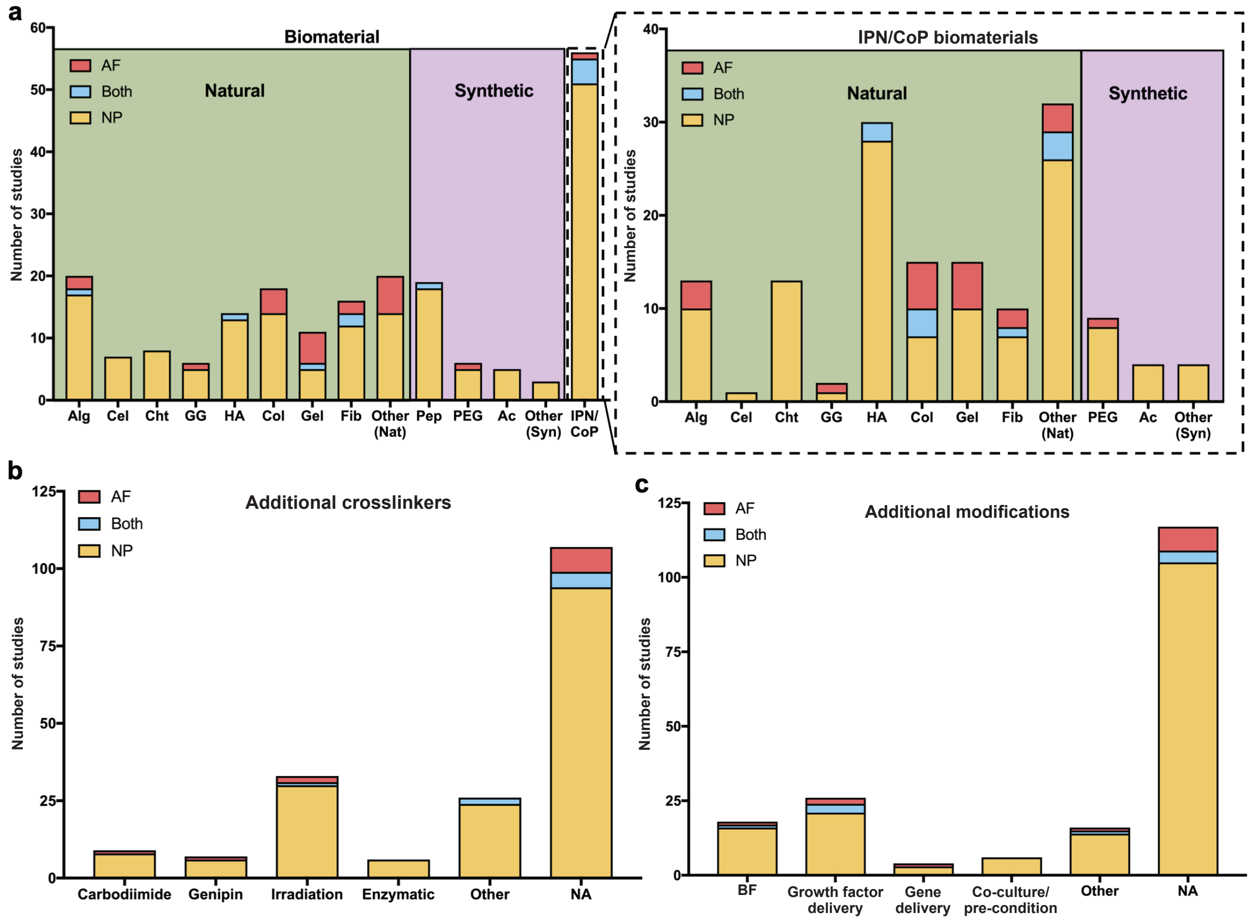

Fig. 4. Summary of biomaterial inputs for IVD cell delivery biomaterial studies. (**a**) Histogram showing the frequency of studies using different biomaterial carriers. For studies that used interpenetrating network/co-polymeric (IPN/CoP) biomaterial strategies, the frequency of individual biomaterial components was quantified. Alginate (Alg), cellulose (Cel), chitosan (Cht), gellan gum (GG), hyaluronic acid (HA), collagen (Col), gelatine (Gel), fibrin (Fib), natural (Nat), peptide (Pep), polyethylene glycol (PEG), acrylates (Ac), synthetic (Syn) and interpenetrating network/co-polymeric (IPN/CoP). (**b**) Histogram showing the frequency of studies using various additional crosslinking agents in biomaterial carriers. (**c**) Histogram showing the frequency of studies using various additional modifications to cell-biomaterial delivery systems. Biological functionalisation (BF). For each bar, the red portion represents studies targeting the AF, the yellow portion represents studies targeting the NP and the blue portion represents studies targeting Both IVD regions.

biomaterial carriers (4/8 = 50.0 %); hyaluronic acid (2/4 = 50.0 %) and collagen (3/4 = 75.0 %) were the most common components of these biomaterials.

Additional crosslinking mechanisms used to enhance the biomechanical properties of the investigated biomaterials were also summarised. This was considered a crucial piece of the analysis because IVD cell delivery biomaterials will experience high mechanical demands due to the load-bearing function of the IVD, and crosslinking is a simple method of increasing biomechanical modulus and strength of a biomaterial. Interestingly, most of the reviewed studies did not use any additional crosslinking strategies, beyond those crosslinking steps essential for hydrogel formation (107/183 = 58.5 %) (Fig. 4**b**). Of the studies that utilised additional crosslinking mechanisms, irradiation-based crosslinking was the most common for NP-targeted (30/69 = 43.4 %) and AF-targeted (2/4 = 50.0 %) cell delivery strategies. Studies investigating biomaterials targeting Both IVD regions used irradiation (1/3 = 33.3 %) or 'Other' (2/3 = 66.7 %) crosslinking mechanisms.

Lastly, additional modifications to the biomaterial carrier in order to enhance delivered cell function were summarised, and it was found that most studies did not investigate any additional means of enhancing the biological repair capacity of their delivery strategy (119/183 = 65.0 %) (Fig. 4**c**). Of the studies that made additional modifications to their IVD cell delivery biomaterials, the most popular modification was the incorporation of growth factors into biomaterials targeting the NP (24/57 = 42.1 %), AF (2/4 = 50.0 %) and Both (3/4 = 75.0 %). For NP-targeted biomaterials, biological functionalisation was also common (18/57 = 31.6 %).

Trends in biological and biomechanical evaluations

The biological and biomechanical outcome measurements used to characterise IVD cell delivery

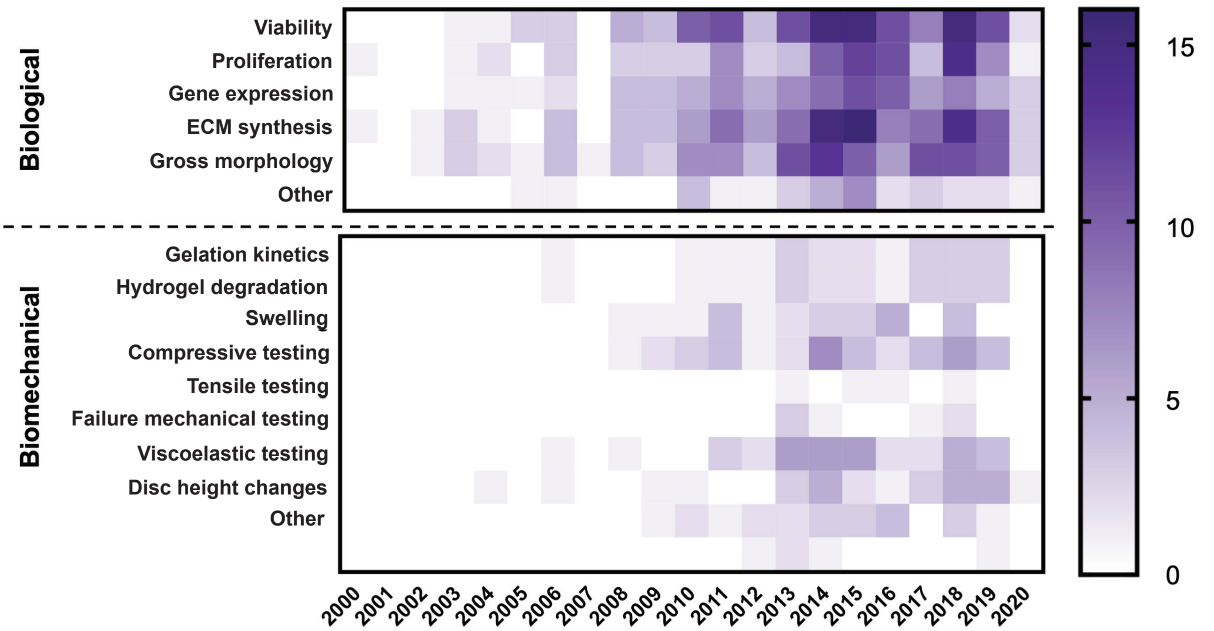

Fig. 5. Summary of biological and biomechanical outcome measurements for IVD cell delivery biomaterial studies. Heatmap showing the frequency of various biological and biomechanical outcome measurements as a function of time. Colour intensity of a cell relates to the number of studies published in that year which measure a particular outcome.

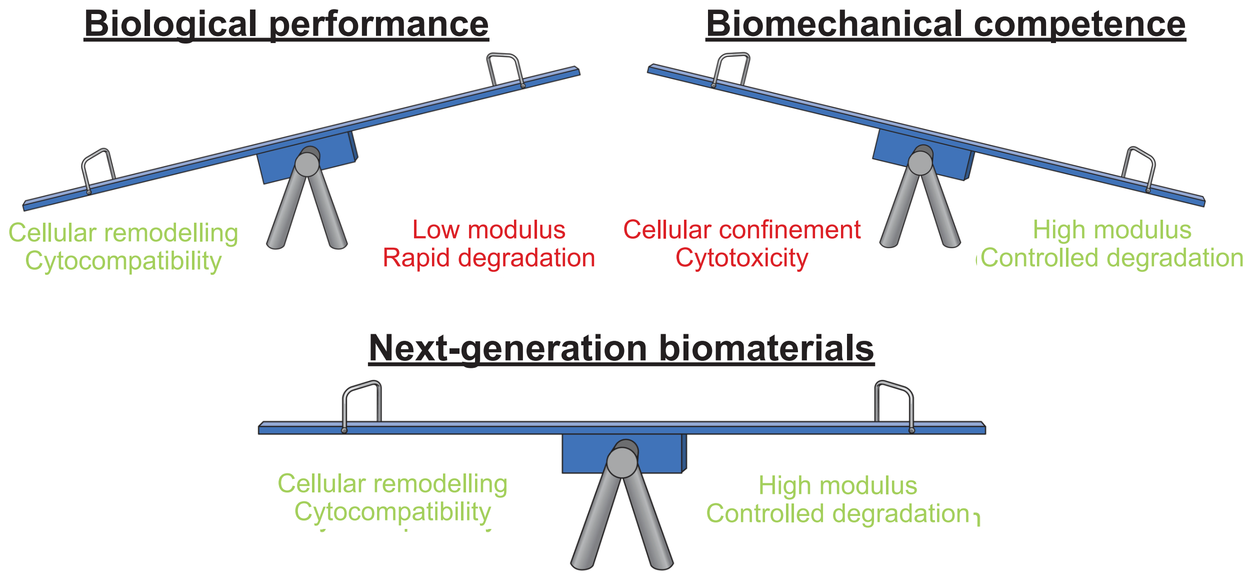

Fig. 6. Illustration of the 'seesaw' effect, found in numerous studies that reported biomaterial modulus; whereby, formulations with higher moduli had inferior cellular performance, and vice versa. By this effect, biomaterial carriers could be designed for biomechanical competence or biological performance. Next-generation biomaterials designed to achieve both biomechanical competence and biological performance are an area warranting further development.

strategies were summarised and evaluated in the 183 reviewed studies. As a whole, it was found that all articles measured at least one biological outcome (*i.e.* viability, proliferation, gene expression, ECM synthesis, gross morphology and 'Other') (Fig. 5). For NP-targeted strategies, cell viability was most commonly measured (127/163 = 77.9 %) and most articles also measured ECM synthesis (108/163 = 66.3 %), gross morphology (99/163 = 60.7 %), gene expression (83/163 = 50.9 %) and proliferation (80/163 = 49.0 %). Fewer AF-targeted studies measured viability (3/12 = 25.0 %) and most assessed the success of the repair strategy using gross morphology (10/12 = 83.3 %) and ECM synthesis (9/12 = 75.0 %). A similar trend was observed in studies investigating biomaterials targeting Both IVD regions; whereby, few studies measured viability (1/8 = 12.5 %) and most measured ECM synthesis (5/8 = 62.5 %), gross morphology (4/8 = 50 %) and proliferation (4/8 = 50 %).

Biomechanical outcomes were much less frequently measured (*i.e.* gelation kinetics, hydrogel degradation, swelling, compressive testing, viscoelastic testing, tensile testing, failure

mechanical testing, disc height changes and 'Other'). Out of all the NP-targeted studies, compression testing (35/163 = 21.5 %) and viscoelastic testing (34/163 = 20.9 %) were most frequently evaluated. Compression testing (3/12 = 25 %, 1/8 = 12.5 %) and viscoelastic testing (2/12 = 16.7 %, 1/8 = 12.5 %) were also most common for studies targeting the AF and studies targeting Both IVD regions. For AF-targeted studies, hydrogel degradation (2/12 = 16.7 %), swelling (2/12 = 16.7 %) and disc height changes (2/12 = 16.7 %) were measured at a similar frequency. In studies investigating biomaterials targeting Both IVD regions, gelation kinetics (1/8 = 12.5 %), hydrogel degradation (1/8 = 12.5 %) and swelling (1/8 = 12.5 %) were measured at a similar frequency to compressive and viscoelastic testing. Given the high mechanical demands experienced by the IVD, biomechanical assessments of cell delivery biomaterial strategies were considered to be essential to ensure the biomaterial does not herniate. Furthermore, there was an interest in how the biomechanical properties of tested IVD cell delivery biomaterials compare to their target tissue and what biomaterial inputs led to these properties. For this reason, the next set of analyses focused on studies that reported biomaterial moduli from compressive and viscoelastic testing and summarised their findings to elucidate themes of effective IVD cell delivery biomaterials.

General themes in biomechanically effective IVD cell delivery biomaterial strategies

Approximately one third (58/183 = 31.7 %) of the studies included in this systematic review reported compressive or shear moduli of the IVD cell delivery biomaterial *in vitro*. Through investigation of these 58 studies, 2 common themes were found. The first theme to emerge was a competition between biological performance and biomechanical competence, or 'seesaw' effect, found in 43.1 % (25/58) of studies that reported *in vitro* compressive and shear moduli (Fig. 6). In this subset of studies (Table 1), groups evaluated how biological and biomechanical outcomes varied when modifying the concentration of macromer, additional crosslinker or an additional modification used to enhance biomaterial modulus. In 72.0 % (18/25) of these papers, marked in green, it was found that to achieve the greatest moduli, the concentration of macromer, additional crosslinker or additional modification was increased to the point of promoting less survival or ECM synthesis of encapsulated cells. Therefore, there is a balance, or 'seesaw' between biological performance and biomechanical competence. Studies which avoided this phenomenon, marked in yellow, (7/25 = 28%) generally reported lower moduli that did not match or just approached the biomechanical properties of the intended target tissue. No currently studied biomaterials are able to match AF material properties with high cell viability and ECM elaboration.

A second theme to emerge was *in vitro* construct maturation, or an increase in moduli values of the construct throughout the culture duration period due to ECM elaboration. Constructs matured in 22.4 % (13/58) of the studies that reported compressive or shear moduli for their material (Table 2). In this subset of studies, groups evaluated how the biomechanical properties of cell-laden biomaterials changed over time. Most of these studies (10/13 = 76.9 %), marked in green, demonstrated that the moduli of cell-laden biomaterial constructs increased throughout culture; the median increase in modulus was approximately two-fold. 23.1 % (3/13) of these studies, marked in yellow, reported that the moduli of cell-laden biomaterial constructs were constant over time, while the moduli of control acellular constructs decreased. Only one of these studies (1/13 = 7.7 %), marked in red, reported a decline in the moduli of cell-laden biomaterial constructs.

A small subset of studies which reported *in vitro* compressive and shear moduli demonstrated both a 'seesaw' effect and construct maturation (7/58 = 12.1 %) (Table 3). In these studies, the competition between biomechanical competence and biological performance was recognised, then constructs with high biological performance were matured. In 71.4 % (5/7) of these studies, marked in green, groups experienced a 'seesaw' effect, then showed that formulations most conducive to biological function experienced significant increases in moduli throughout culture. These increases were not seen in formulations that had greater initial moduli, closer to the target tissue. The remaining 28.6 % (2/7) of studies, marked in yellow, experienced a 'seesaw' phenomenon and showed

Table 1. Studies reporting biomaterial moduli for range of formulations. Studies marked in green experienced a 'seesaw' effect; whereby, to achieve the greatest moduli, the concentration of macromer, additional crosslinker or additional modification was increased to the point of promoting less survival or ECM synthesis of encapsulated cells. Studies marked in yellow varied the concentration of a component in the IVD cell delivery biomaterial system, but did not experience this phenomenon. Black and coloured arrows indicate changes in the inputs and outputs for the IVD cell delivery biomaterial system, respectively. ↑ indicates an increase in the concentration of the specified component in the IVD cell delivery biomaterial system. + indicates the inclusion of the specified component in the IVD cell delivery biomaterial system. ↑, ↔ or ↓ indicate that the resultant biological or biomechanical output measured increased, remained the same, or decreased, respectively, as a result of the change in the IVD cell delivery biomaterial system (*e.g.* "↑ Genipin conc., ↓, NP gene expression" means that as genipin concentration increased, the NP gene expression decreased). G′, G″ and G* values are reported at 1 Hz. (**Table 1** on next page.)

Table 1. (Legend on previous page)

Study	Macromer	Crosslinker	Additional modifications	Culture duration	Primary biological findings	Primary biomechanical findings
**NP studies**						
Chou *et al.*, 2009	Alg<br>[2 – 3 %]	Irradiation<br>(Irgacure 2959)<br>[0.05 %]	NA	**2 weeks**	↑ Methacrylation<br>↓ Viability	E_y: ~0.6 - 9 kPa;<br>↑ Methacrylation and Alg conc.<br>↑ E_y
Growney Kalaf *et al.*, 2016	Alg<br>[1 % w/v]	Other<br>($CaCO_3$ & GDL)<br>[15 - 75 &<br>30 - 150 mmol/L]	NA	**<2 h**	↑ $CaCO_3$:GDL ratio<br>↓ Viability	G′ = ~295 - 2100 Pa, G″ = ~19 - 1300 Pa;<br>↑ $CaCO_3$:GDL ratio<br>↑ G′ and G″
Zhou *et al.*, 2018a	Col<br>[3 mg/mL]	Genipin<br>[0 - 1 % w/v]	NA	**2 weeks**	↑ Genipin conc.<br>↓ NP gene expression<br>↓ NP ECM synthesis	E_y: ~1 - 20 MPa (AFM);<br>↑ Genipin conc.<br>↑ E_y
Khang *et al.*, 2015	GG<br>[0.75 % - 2 % w/v]	NA	NA	**2 weeks**	↑ HA-GG conc.<br>↑ Viability	G′: ~20 - 60 kPa;<br>↑ HA-GG conc.<br>↓ G′
Pereira *et al.*, 2011	GG<br>[0.75 - 2 % w/v]	NA	Other<br>(GG MPs)	**2 weeks**	↑ LAGG MP conc.<br>↔ Cell viability	G′: ~20 - 80 kPa;<br>↑ LAGG MP conc.<br>↑ G′
Moss *et al.*, 2011	HA<br>[1.5 % w/v]	Other<br>(PEGDA)<br>[0.8 %]	Other<br>(Elastin-like peptide)	**3 weeks**	+ Elastin-like peptide<br>↔ Cell viability	H_A: ~17 - 31 kPa;<br>+ Elastin-like peptide<br>↑ H_A
Toh *et al.*, 2012	HA<br>[2 % w/v]	Enzymatic<br>(HRP & H_2O_2)<br>[0.125 U/mL &<br>500 - 1000 µmol/L]	NA	**3 weeks**	↑ H_2O_2 conc.<br>↓ Chondrogenic ECM synthesis	E_y: ~5 - 12 kPa<br>↑ H_2O_2 conc.<br>↑ E_y
Francisco *et al.*, 2014	PEG<br>[2 - 10 % w/v]	Irradiation<br>(Irgacure 2959)<br>[0.1 % w/v]	BF<br>(Laminin-111)<br>[0 - 1000 µg/mL]	**1 week**	↑ PEG conc.<br>↓ Cell viability	G*: ~0.4 - 20 kPa;<br>↑ PEG conc.<br>↑ G*
Ligorio *et al.*, 2019	Pep<br>[10 - 20 mg/mL]	NA	Other<br>(Graphene oxide)<br>[0 - 0.5 mg/mL]	**1 week**	+ Graphene oxide<br>↔ Cell viability	G′: ~4 - 16 kPa;<br>+ Graphene oxide<br>↑ G′
Tao *et al.*, 2015	Pep<br>[1 %]	NA	BF<br>(BMP-7 functional peptides)	**4 weeks**	+ RAD-KPS<br>↑ Proliferation<br>↑ ECM synthesis	G′: ~100 - 200 Pa, G″: ~20 - 40 Pa;<br>+ RAD - KPS<br>↓ G″ and G′
Wang *et al.*, 2014	Pep<br>[1 % w/v]	NA	BF<br>(Link-N functional peptides)	**2 weeks**	+ Link-N functional peptide<br>↑ Chondrogenic gene expression	G′: ~5 - 6 kPa, G″: ~0.8 - 0.9 kPa;<br>+ Link-N functional peptide<br>↔ G′ and G″
Alinejad *et al.*, 2018	Cht & CS<br>[2 % & 0 - 1 % w/v]	Other<br>(BGP & SHC)<br>[0.1 - 0.4 & 0.075 mol/L]	NA	**1 week**	↑ CS conc.<br>↑ Metabolic activity (1 week)	G′: ~5 - 20 kPa;<br>↑ CS conc.<br>↓ G′
Alinejad *et al.*, 2019	Cht<br>[2 % w/v]	Other<br>(BGP & SHC)<br>[0.1 - 0.4 & 0.075 mol/L]	NA	**2 weeks**	↑ SHC/BGP conc.<br>↑ Viability and metabolic activity	E_y: ~2 - 8 kPa, G′: ~1 - 8 kPa, G″<br>↑ SHC/BGP conc.<br>↑ E_y and G′
Calderon *et al.*, 2010	Col & HA<br>[5 & 1.57 mg/mL]	Carbodiimide<br>(EDC/NHS)<br>[8 - 48 mmol/L]	NA	**3 weeks**	↑ EDC/NHS conc.<br>↓ DNA content<br>↓ NP gene expression	E_y: ~1 - 8 kPa;<br>↑ EDC/NHS conc.<br>↑ E_y
Frith *et al.*, 2013	HA & PEG<br>[25 & 100 mg/mL]	Enzymatic<br>(HRP & H_2O_2)<br>[0.25 U/mL &<br>1 - 5 µmol/L]	BF<br>(PPS)<br>[5 µg/mL]	**3 weeks**	+ PPS<br>↑ Chondrogenic ECM synthesis	G′: ~1 - 5 kPa, G″: ~1 - 10 Pa;<br>+ PPS<br>↓ G′
Frith *et al.*, 2014	HA & PEG<br>[15 & 16.5 mg/mL]	Enzymatic<br>(HRP & H_2O_2)<br>[0.25 U/mL &<br>1 - 5 µmol/L]	BF<br>(PPS)<br>[5 – 20 µg/mL]	**3 weeks**	↑ PPS<br>↑ ECM synthesis	G′: ~1 - 5 kPa, G″: ~0.2 - 100 Pa;<br>↑ HA - PPS or PPS<br>↔ G′ or G″
Gan *et al.*, 2017	Gel, Dex & PEG<br>[10 wt% total,<br>1:1.1:8 of Dex/Gel:PEG]	Irradiation<br>(Irgacure 2959)<br>[0.5 % w/v]	NA	**4 weeks**	↑ PEG:Dex/Gel ratio<br>↑ Viability and proliferation<br>↑ ECM synthesis	E_y: ~2 - 60 kPa, G*: ~12 - 46 kPa;<br>↑ PEG : Dex/Gel ratio<br>↓ E_y and G*
Li *et al.*, 2014	Fib & HA<br>[6 - 13 &<br>0.7 - 1.3 mg/mL]	NA	NA	**2 weeks**	↑ Fib:HA ratio<br>↑ NP gene expression	G′: ~80 - 350 Pa<br>↑ Fib : HA ratio<br>↓ G′
Park *et al.*, 2011	Fib & HA<br>[10 & 10 mg/mL]	NA	Other<br>(Silk)<br>[1-2 %]	**4 weeks**	↑ Silk conc.<br>↓ Chondrogenic gene expression<br>↓ ECM synthesis	E_y: ~3 - 10 kPa;<br>↑ Silk conc.<br>↑ E_y
Peroglio *et al.*, 2012	HA & Pep<br>[0.5 % w/v]	NA	NA	**1 week**	↑ Pep molecular weight<br>↓ Viability	G′: ~0.005 - 16 kPa;<br>↑ Pep molecular weight<br>↑ G′
Zhou *et al.*, 2018b	Col & CS<br>[3 & 8.1 mg/mL]	Genipin<br>[0.01 - 1 % w/v]	NA	**2 weeks**	↑ Genipin conc.<br>↓ Viability	G′: ~2400 Pa, G″: 230 Pa;<br>↑ Genipin conc.<br>↑ G′ and G″
Zhu *et al.*, 2017	Cht & HA<br>[0.2-1.2 % &<br>0.1 - 0.6 %)	Other<br>(BGP)<br>[3 %]	Growth Factor Delivery<br>(Kartogenin)	**26 d**	↑ Cht conc.<br>No biological evaluation	E_y: ~0.9 - 2.9 MPa<br>↑ Cht conc.<br>↑ E_y
**AF studies**						
Cruz *et al.*, 2018	Fib<br>[35 - 140 mg/mL]	Genipin<br>[1 - 6 mg/mL]	NA	**7 weeks**	↑ Genipin conc.<br>↓ Viability<br>↓ GAG synthesis	E_y: ~30 - 200 kPa, G*: ~7 - 65 kPa;<br>↑ Fib and Genipin conc.<br>↑ E_y and G*
Pereira *et al.*, 2018	GG<br>[0.75 - 1 wt%]	NA	Other<br>(Cel nanocrystals)<br>[1.25 - 2.5 wt%]	**2 weeks**	↑ GGMA/Cel nanocrystal conc.<br>↓ Viability	E_y: ~45 - 55 kPa, G*: ~0.009 - 0.6 kPa;<br>↑ GGMA/Cel nanocrystal conc.<br>↑ G*
**Both studies**						
Piluso *et al.*, 2019	Gel<br>[5 % w/v]	Irradiation<br>(LAP)<br>[0.3 % w/v]	Other<br>(Starch nanocrystals)<br>[0 - 0.5 wt%]	**1 week**	↑ Starch nanocrystal conc.<br>↔ Cell viability	E_y: ~1.5 - 3 kPa;<br>↑ Starch nanocrystal conc.<br>↑ E_y

Table 2. Studies reporting biomaterial moduli of constructs cultured over time. Studies marked in green, yellow and red demonstrated that the moduli of cell-laden biomaterial constructs increased, remained constant, or decreased, respectively, throughout the specified culture duration. Coloured arrows indicate changes in the outputs for the IVD cell delivery biomaterial system over culture time. ↑, ↔ or ↓ indicate that the resultant biological or biomechanical output measured increased, remained the same, or decreased, throughout culture. G′, G″ and G* values are reported at 1 Hz.

Study	Macromer	Additional crosslinker	Additional modifications	Culture duration	Primary biological findings	Culture	Primary biomechanical findings
Chou *et al.*, 2009	Alg<br>[2 %]	Irradiation<br>(Irgacure 2959) [0.05 %]	NA	**4 weeks**	↑ Proteoglycan synthesis	**8 weeks**	↑ E_y: ~1.3 - 4.3 kPa (~200 %)
Foss *et al.*, 2014	Alg<br>[0.5 - 4 %]	NA	Other<br>(GCSN & CS)<br>[125 - 500 & 100 - 400 mg/mL]	**4 weeks**	↑ Cell viability and proliferation<br>↑ Col II synthesis<br>↓ GAG and water content	**4 weeks**	↑ H_A: ~0.2 - 1.4 MPa (~100 %)
Reza *et al.*, 2010a	Cel<br>[1 - 5 %]	Irradiation<br>(Irgacure 2959) [0.05 %]	NA	**1 week**<br>**2 weeks**	↑ Cell viability<br>↑ ECM synthesis	**2 weeks**	→ E_y: ~2 - 5 kPa<br>(N.S. change)
Reza *et al.*, 2010b	Cel<br>[2.5 %]	Irradiation<br>(Irgacure 2959) [0.05 %]	NA	**4 weeks**	↑ DNA content<br>↑ ECM synthesis	**4 weeks**	→ E_y: ~5 - 20 kPa<br>(D0 N.R.)
Gupta *et al.*, 2011	Cel<br>[2 %]	Irradiation<br>(Irgacure 2959) [0.05 %]	NA	**3 weeks**	↑ Proliferation<br>↑ Col II, CS and GAG synthesis	**3 weeks**	↑ E_y: ~1 - 2 kPa (~25 %)
Gupta *et al.*, 2014	Cel<br>[1.75 %]	Irradiation<br>(Irgacure 2959) [0.05 %]	NA	**8 weeks**	↑ ECM synthesis	**8 weeks**	↑ E_y: ~2 - 30 kPa (~900 %)
Varma *et al.*, 2018	Cel<br>[2 %]	APS/TEMED<br>[10 mM & 10 mM]	NA	**5 weeks**	↑ Cell viability<br>↑ ECM synthesis	**5 weeks**	↑ E_y: ~5 - 30 kPa (~500 %)
Silva-Correia *et al.*, 2013	GG<br>[2 % w/v]	Irradiation<br>(Methyl benzoylformate)<br>[0.1 % w/v]	NA	**3 weeks**	↑ Cell viability	**3 weeks**	↑ G′: ~60 - 120 kPa (~50 %)
Kim *et al.*, 2015	HA<br>[1 % w/v]	Irradiation<br>(Irgacure 2959) [0.5 % w/v]	NA	**8 weeks**	↑ Cell viability<br>↑ NP-specific gene expression<br>↑ ECM synthesis	**8 weeks**	↑ E_y: ~2 - 160 kPa (~8000 %)<br>↑ G*: ~1 - 2 MPa (D0 N.R.)
Kumar *et al.*, 2014	Ac<br>[13.5 % w/v]	Irradiation<br>(Irgacure 2959) [0.5 % w/v]	NA	**2 weeks**	↑ Cell viability and proliferation<br>↑ ECM synthesis	**2 weeks**	↔ E_y: ~2.5 - 4.5 kPa<br>(N.S. change)
Barreto-Henriksson *et al.*, 2019	Pep<br>[1 %]	NA	NA	**3 weeks**	↑ GAG synthesis	**4 weeks**	↑ G′: ~82 - 138 kPa (~70 %)<br>↑ G″: ~16 - 25 kPa (~55 %)
Wan *et al.*, 2016	Pep<br>[25 - 35 mg/mL]	NA	NA	**2 weeks**	↑ Cell viability<br>↑ ECM synthesis	**2 weeks**	↓ G′: ~5 - 20 kPa (~75 %)<br>↔ G″: ~1 - 3 kPa (N.S change)
Mercuri *et al.*, 2013	Decellularised porcine NP matrix	Carbodiimide<br>(EDC/NHS & PGG)<br>[30 mmol/L/6 mmol/L & 0.15 %]	NA	**2 weeks**	↑ Cell viability<br>↑ ECM synthesis	**2 weeks**	↑ G*: ~4 - 20 kPa (~100 %)

Table 3. Studies reporting biomaterial moduli for a range of formulations and constructs cultured over time. Studies marked in green and yellow demonstrated that the moduli of cell-laden biomaterial constructs increased or remained constant, respectively, throughout the specified culture duration. Black and coloured arrows indicate changes in the inputs and outputs for the IVD cell delivery biomaterial system, respectively. ↑ and ↓ indicates an increase or decrease, respectively, in the concentration of the specified component in the IVD cell delivery biomaterial system. ↑, ↔ or ↓ indicate that the resultant biological or biomechanical output measured increased, remained the same, or decreased, respectively, as a result of the change in the IVD cell delivery biomaterial system or throughout the specified culture time. G′, G″ and G* values are reported at 1 Hz.

Study	Macromer	Crosslinker	Culture duration	Primary biological findings	Culture duration	Primary biomechanical findings
Gupta *et al.*, 2015	Cel<br>[1.5 - 3.5 %]	Irradiation<br>(Irgacure 2959)<br>[0.05 %]	**1 week**<br>**6 weeks**	1.5 % Cel hydrogel<br>↑ Acan and Col II gene expression<br>↑ GAGs and Col II synthesis	**6 weeks**	↑ E_y: ~2 - 11 kPa (~130 %) of 1.5 % Cel hydrogel<br>↓ E_y of 3.5 % Cel hydrogel
Lin *et al.*, 2016	Cel<br>[2 - 4 %]	Irradiation<br>(Irgacure 2959)<br>[0.05 %]	**4 weeks**	↓ CMC conc.<br>↑ ECM synthesis	**4 weeks**	↓ CMC conc.<br>↑ E_y: ~2 - 11 kPa (~130 %)
Halloran *et al.*, 2008	Col, HA & Acn<br>[5, 0.55 & 1 mg/mL]	Enzymatic<br>(mTGase)<br>[0.05 mg/mL]	**1 week**	+ HA, Acn and mTGase<br>↔ Cell viability<br>↑ sGAG retention	**1 weeks**	+ mTGase<br>↑ G′: ~500 - 1250 Pa<br>↑ E_y (No value, reported as sig. increased)
Hayami *et al.*, 2011	PCL & Cht<br>[40 - 100 & 10 - 60 % vol%]	Irradiation<br>(Irgacure 2959)<br>[0.1 % w/v]	**2 weeks**	30 vol % xMGC-9.3KELAST scaffold<br>↑ Proliferation<br>↑ Metabolic activity<br>↑ ECM synthesis	**2 weeks**	↑ Elastomer concentration<br>↓ Equilibrium modulus<br><br>30 vol% xMGC - 9.3KELAST<br>↑ Equilibrium modulus: ~100 - 2100 kPa (~500 %)
Hayami *et al.*, 2013	PCL & Cht<br>[25 - 30 % w/v & 6 - 8 %]	Irradiation<br>(Irgacure 2959)<br>[0.1 % w/v]	**1 week**<br><br>**8 weeks**	↑ Elastomer conc.<br>↔ Cell viability<br><br>7030TMCCL<br>↑ DNA, GAG and collagen	**8 weeks**	↑ Elastomer conc.<br>↓ Equilibrium modulus<br><br>7030TMCCL<br>↑ Equilibrium modulus: ~600 - 1500 kPa (~10 %)
Hayami *et al.*, 2016	HA, Cht & CS<br>[6 %, 6 % & 20 % w/v]	Irradiation<br>(Irgacure 2959)<br>[0.1 % w/v]	**5 weeks**	MGC - MCS & MHA - MCS blends<br>↑ Total DNA<br>↑ ECM synthesis	**5 weeks**	MGC-MCS & MHA-MCS blends<br>↑ Equilibrium Modulus: ~100 - 275 kPa (~30 %)
Navaro *et al.*, 2015	Tetronic1307 & Fib<br>[Tetronic 1307 : Fib, 1 : 4]	Irradiation<br>(Irgacure 2959)<br>[0.1 % w/v]	**2 weeks**	1 kPa Matrix<br>↑ Proliferation and chondrogenic differentiation<br><br>2 kPa Matrix<br>↑ Osteogenic differentiation	**2 weeks**	1 kPa matrix<br>↑ G′: ~0.2 - 0.6 kPa (~200 %)<br><br>2 kPa matrix<br>↔ G′

Table abbreviations	
7030TMCCL	poly(ε-caprolactone-co-trimethylene carbonate)
Ac	acrylates
Acn	aggrecan
AFM	atomic force microscopy
Alg	alginate
APS	ammonium persulphate
BGP	β-glycerophosphate
BMP-7	bone morphogenic protein-7
BF	biological functionalisation
Cel	cellulose
Cht	chitosan
Col	collagen
CS	chondroitin sulphate
Dex	dextran
EDC	1-ethyl-3-(3-dimethylaminopropyl)-carbodiimide
Ey	Young's compressive modulus
Fib	fibrin
G*	complex shear modulus at 1 Hz
G'	storage modulus at 1 Hz
G"	loss modulus at 1 Hz
GCSN	glucosamine
GDL	glucono-δ-lactone
Gel	gelatine
GG	gellan gum
HA	hyaluronic acid
HRP	horse radish peroxidase
LAP	lithium phenyl(2,4,6-trimethylbenzoyl)-phosphinate
MCS	methacrylated chondroitin sulphate
MGC	methacrylated glycol chitosan
MHA	methacrylated hyaluronic acid
MPs	microparticles
mTGase	microbial transglutamase
NHS	N-hydroxysuccinimide
PCL	polycaprolactone
PEG	polyethylene glycol
PEGDA	polyethylene glycol diacrylate
Pep	peptide
PGG	pentagalloyl glucose
PPS	pentosan polysulphate
PRP	platelet rich plasma
SHC	sodium hydrogen carbonate
TEMED	tetramethylethylenediamine

that the constructs with low initial moduli were biologically favourable since ECM was produced and moduli remained constant over time, while the moduli of acellular constructs with initially high moduli produced little ECM and moduli significantly decreased with culture time.

Discussion

Research investigating injectable cell delivery biomaterials for IVD repair started as a field approximately 20 years ago and has since grown rapidly to include more than 180 published studies. In the first study identified from 2000, Stern *et al*. investigated a fibrin/hyaluronic acid matrix to enhance proliferation and ECM elaboration of porcine NPCs; overall, few biological assessments were performed and no biomechanical outcomes were reported (Stern *et al*., 2000). 20 years later, sophisticated cellular and biomaterial strategies are being applied to enhance the biological and biomechanical performance of IVD cell delivery biomaterials intended for the NP, AF or Both IVD regions. For example, Hu *et al*. used a thermosensitive hydrogel to deliver growth differentiation factor 5 (GDF5)-transfected human induced pluripotent stem cells (iPSCs) to restore disc height index in a rat caudal IVD injury model (Hu *et al*., 2020). RGD peptide sites were incorporated into polysaccharide cell delivery hydrogels by Wang *et al*. to enhance encapsulated cell function (Wang *et al*., 2019). Alinejad *et al*. and Piluso *et al*. used additional crosslinking agents and starch nanocrystals, respectively, to enhance the mechanical properties of their cell delivery biomaterials, while maintaining encapsulated cell function (Alinejad *et al*., 2019; Piluso *et al*., 2019). All listed studies evaluated their proposed strategies using a variety of biological and biomechanical outcome measurements. These rapid scientific advancements over the past 2 decades are likely to accelerate as the field continues to grow, and it is expected that more intricate IVD cell delivery biomaterial strategies will be developed with greater chances of clinical success.

Most IVD cell delivery studies target the NP for biomaterial-aided cell delivery. This is expected, considering that injectable hydrogels have a highly hydrated amorphous structure more similar to the NP than the AF (Goins *et al*., 2005). There were notably fewer studies investigating the AF as a target for cell delivery using an injectable biomaterial carrier, likely because the fibre-reinforced nature of AF tissue and need to resist tensile stresses is difficult to achieve with injectable hydrogels. AF repair strategies have more often involved devices like the X-Close (Bailey *et al*., 2013) and Barricaid (Parker *et al*., 2016) systems that are designed to close AF defects and prevent reherniation. Additionally, fibre-reinforced tissue engineered constructs to plug AF defects (Sato *et al*., 2003) or recapitulate the organised collagen structure of the AF (Bhattacharjee *et al*., 2012; Nerurkar *et al*., 2007; Park *et al*., 2012) have also been investigated for AF repair, yet they are not injectable. Injectable cell delivery strategies for AF repair and regeneration are compelling because reherniation and recurrent pain remains an unmet clinical need, and because injectable formulations may be applied rapidly during discectomy, or other minimally invasive procedures.

This systematic review shows little consensus on the cell type and cell species used in current IVD cell delivery biomaterial strategies. NPCs and AFCs were very common for studies targeting the NP and AF, respectively. These cell sources are useful for phenotypic characterisation of how IVD cells will behave in experimental biomaterials; however, the low cellularity of aged human IVD tissues (Maroudas *et al*., 1975) and difficulty in surgically harvesting IVD tissue makes use of autologous IVD cells impractical for clinical translation with current technologies. Stem cell sources have greater

availability and possess the ability to proliferate and differentiate into a wide range of lineages (Armstrong *et al.*, 2012). Of the variety of stem cell sources, MSCs were most popular for both NP- and AF-targeted studies, although ASCs and iPSCs were also used. MSCs are easily accessible, patient-specific and have the capacity to differentiate into various mesodermal cell types, such as NPCs and AFCs (Richardson *et al.*, 2010; Richardson *et al.*, 2016). Wang *et al.* was the only study to investigate the use of NP-specific progenitor cells for IVD cell delivery (Wang *et al.*, 2019). IVD-derived stem/progenitor cells were first identified as a promising cell source in 2007 (Risbud *et al.*, 2007); however, more biological characterisation is required before these cells can be more widely adopted (Hu *et al.*, 2018). The high frequency by which various stem cell sources are being used in IVD cell delivery biomaterial studies enhances our knowledge and increases their likelihood for clinical translation.

Cells used in reviewed studies were predominantly derived from humans, which highlights a great focus on clinical translatability. Rabbit, cow, pig and rat were the next most commonly used animal cell types. Cells used from different species are likely selected because they are derived from accepted preclinical models of IVD degeneration and repair; thus, enabling more rapid progression from *in vitro* to *in vivo* testing (Daly *et al.*, 2016). The rat and rabbit are in the small animal range with benefits of being somewhat more cost-effective, and retaining anatomical features including facet joints, paravertebral muscles and ligaments similar to the human spine (Kroeber *et al.*, 2002; Silberberg *et al.*, 1979). Cow and pig are commonly used large animal models that can be relatively easily acquired from abattoirs, enabling high force application and nutrient transport distances that more closely simulate loading and nutrient transport challenges of the human IVD (Alini *et al.*, 2008; Beckstein *et al.*, 2008; Daly *et al.*, 2016). This review clarified the lack of consensus on animal models for IVD research; different species and model systems may be used to evaluate specific scientific design goals in each study.

No clear consensus was found on optimal IVD cell delivery biomaterial carriers. The most commonly tested biomaterials were IPN/CoP biomaterials, which combine multiple polymer networks to form materials that are biologically and biomechanically tuneable (Ullah *et al.*, 2015; Vedadghavami *et al.*, 2017). Natural biomaterials were a common component of these IPN/CoP biomaterials, likely because they are composed of repeating subunits commonly metabolised by humans and they contain binding motifs which mimic natural ECM to support the function of encapsulated cells (Parisi *et al.*, 2018). Additional crosslinking, beyond that required to solidify the hydrogel, was employed to increase the biomechanical properties of low modulus materials (Oryan *et al.*, 2018). Additional modifications, such as the incorporation of nanoparticles into the biomaterial matrix were also used to increase the moduli of biomaterials with initially low moduli that had favourable biological performance (Merino *et al.*, 2015). Conversely, biological functionalisation, incorporation of growth factors/gene vectors, and pre-conditioning/co-culturing delivered cells were strategies to enhance the biological repair capacity of high modulus biomaterials. Overall, a minority of studies used additional crosslinking agents and additional modifications for IVD cell delivery biomaterials, suggesting these strategies require further research before clinical implementation.

All studies reported biological outcomes at either the molecular, cellular or macroscopic hierarchical scale; however, biomechanical outcomes were less commonly reported and mostly evaluated *in vitro*. Molecular and cellular assessments (*i.e.* viability, proliferation and gene expression) are important preliminary benchmarks for an IVD repair strategy, but macroscopic assessments (*i.e.* ECM synthesis and gross morphology) more rigorously demonstrate that the biomaterial provides a supportive niche to promote cell-mediated healing. Molecular, cellular and macroscopic biological outcomes were reported, at a similar high frequency over time, indicating that biological efficacy of IVD cell delivery biomaterials is being appropriately challenged on multiple scales and model systems. Conversely, biomechanical outcome measurements were only reported in approximately 60 % of the reviewed studies and the biomechanical outcome measurements most commonly reported were *in vitro* compressive and viscoelastic testing. Tensile testing was rarely performed, which is perhaps not surprising given the injectable biomaterials reviewed have low tensile moduli; however, it does demonstrate an unmet challenge given the tensile demands of the IVD under loading (Jacobs *et al.*, 2013; Nerurkar *et al.*, 2010; Tsantrizos *et al.*, 2005). These *in vitro* biomechanical tests determine if the biomaterial properties mimic the target tissue; however, *in situ* biomechanical testing (*e.g. ex vivo* or *in vivo* failure testing) is required to assess the herniation risk and functional biomechanical restoration of a potential IVD cell delivery biomaterial. Such *in situ* biomechanical outcome measurements were reported much less frequently, highlighting the need to evaluate the herniation risk and functional performance of IVD cell delivery biomaterial strategies at multiple hierarchical scales. *Ex vivo* evaluations, using bioreactors that recapitulate the IVD physiological environment, are a useful benchmark in this hierarchy because they can rigorously assess the biological and biomechanical performance of a particular strategy in a highly controlled manner (Gantenbein *et al.*, 2015; Pfannkuche *et al.*, 2020). Testing paradigms to screen IVD repair biomaterials at progressive hierarchical scales exist (Long *et al.*, 2016; Virk *et al.*, 2020), which can be adapted to screen IVD cell delivery biomaterial strategies by including molecular, cellular and macroscopic biological assessments of cells within candidate biomaterials *in vitro*, *ex vivo* and *in vivo*.

Assessing biological and biomechanical outcome measurements at these various hierarchical scales and model systems will best challenge the IVD cell delivery biomaterial strategy, allow for targeted modifications and more rapidly advance to clinical translation.

Balancing biomechanical and biological performance remains an ubiquitous challenge when developing repair strategies for the IVD and other musculoskeletal soft-tissues that experience high mechanical demands (D'Este *et al.*, 2018). Studies that reported compressive and shear moduli to elucidate themes of effective IVD cell delivery biomaterials were, therefore, investigated. One important theme that stood out was the 'seesaw' effect where, in most studies that reported biological and biomechanical outcomes for a range of biomaterial formulations, the biomaterial formulations with the greatest moduli had the poorest biological performance of encapsulated cells (Table 1). For example, Francisco *et al*. demonstrated that increasing polyethylene glycol macromer concentrations from 5 % to 6 % reduced NPC survival but increased complex shear modulus (Francisco *et al*., 2014). Zhou *et al*. reported that increasing genipin additional crosslinker concentration from 0.01 % to 1 % w/v decreased viability and NPC differentiation capacity, but increased Young's modulus (Zhou *et al*., 2018a). Park *et al*. found that incorporating higher concentrations of silk into their biomaterial reduced chondrogenic gene expression and ECM synthesis while increasing Young's modulus (Park *et al*., 2012). Several mechanisms can explain this 'seesaw' phenomenon. First, crosslinking reagents that are somewhat benign at low concentrations, can become cytotoxic to cells at high concentrations (Cao *et al*., 2012; Panebianco *et al*., 2020; Saito *et al*., 2008); thus, the amount of crosslinker required for a biomaterial to achieve the modulus of a load-bearing tissue like the IVD could be too high to simultaneously achieve cytocompatibility, motivating need for innovation. Second, increasing the concentration of macromers and additional crosslinkers reduces biomaterial porosity (Brown and Barker, 2014; Cruise *et al*., 1998), which can limit cell functionality by inhibiting nutrient transport, preventing cell-biomaterial interactions and physically constraining cells (Fan and Wang, 2017). Lastly, substrate stiffness, which is implicitly modified through the concentration of macromers, additional crosslinkers and additional modifications, can negatively influence cell behaviours (Fearing *et al*., 2018; Gilchrist *et al*., 2011; Jiang *et al*., 2016). The few studies that avoided the 'seesaw' effect reported lower moduli and these biomaterials would not likely provide initial biomechanical stabilisation upon injection. Moreover, the increase in macromer and additional crosslinker concentrations required to achieve appropriate moduli was reported to be detrimental to the function of encapsulated cells.

A second theme that emerged was construct maturation where constructs exhibited enhanced biomechanical function with culture duration (Tables 2 & 3). The subset of studies showing substantial construct maturation used natural biomaterials which are biodegradable; thus, the mechanical properties of these materials are expected to decrease over time. The modulus of most cultured biomaterials increased over time, suggesting that the rate of ECM synthesis was more rapid than the biomaterial degradation rate. This was demonstrated for alginate (Chou and Nicoll, 2009), cellulose (Varma *et al*., 2018), gellan gum (Silva-Correia *et al*., 2013), hyaluronic acid (Kim *et al*., 2015), peptide (Barreto-Henriksson *et al*., 2019) and decellularised NP matrix (Mercuri *et al*., 2013) hydrogels. The biomaterials presented in these studies are highly promising for translation because if they have sufficient initial adhesive and biomechanical properties to remain within the IVD space upon implantation, then their *in vitro* performance suggests they can promote cells to repair and regenerate the IVD ECM *ex vivo* and *in vivo*. However, this requires further investigation in appropriate preclinical models before clinical translation. The few studies that did not report an increase in modulus with culture generally demonstrated that their cell-laden constructs had no decline in modulus, while the modulus of acellular control constructs decreased over time (Kumar *et al*., 2014; Reza and Nicoll, 2010a; Reza and Nicoll, 2010b). In general, the rate of change of modulus values are attributed to the rate of ECM synthesis relative to the rate of biomaterial degradation. The hydrogels in green with excellent biomechanical maturation increased less than an order of magnitude, so it is likely that biomechanical properties must start in the range of native tissue or mature over an extensive period of time to approach native tissue material properties. The median increase in biomaterial moduli was greater for studies that had relatively low macromer, additional crosslinker or additional modification concentrations in order to prioritise biological performance; therefore, the initial moduli of the injectable biomaterial started relatively low offering little initial biomechanical competence. Overall, this subset of studies highlights the importance of biomaterial degradation as a parameter for cell delivery biomaterial strategies (Kong *et al*., 2004). At this time, it remains a challenge to develop high modulus biomaterial carriers that can encapsulate delivered cells and promote substantial ECM synthesis.

This systematic review of IVD cell delivery biomaterials had the following limitations. First, studies that only evaluated the performance of cells seeded on top of experimental biomaterials were excluded because two-dimensional cell-biomaterial interactions fail to sufficiently evaluate how cells will behave after injection within a three-dimensional biomaterial carrier (Cukierman *et al*., 2001; Pampaloni *et al*., 2007). Similarly, biomechanical data from previously published acellular studies on biomaterials were not included in this review because they were not characterised as cell carriers in those

studies, and it was difficult to determine whether formulations changed subtly between acellular and cell-seeded studies. This systematic review was able to determine which cell types and biomaterials were most commonly used for IVD cell delivery strategies, but this method could not identify which strategies are best because frequent use does not imply superiority. Applying this logic would inaccurately select for more established strategies and bias against novel strategies. The current analysis also could not determine a single best strategy, because not all studies reported the same outcome measurements, and those which did often used different assays that were not directly comparable. However, this systematic review was able to highlight important themes for IVD cell delivery biomaterial strategies that were biologically and biomechanically effective. This knowledge is expected to help guide future injectable IVD cell delivery biomaterial strategies and accelerate their clinical translation to reduce the burden of chronic discogenic back pain.

Conclusions

This systematic review highlights the rapidly increasing numbers of studies investigating injectable IVD cell delivery biomaterials. Most studies focused on delivering cells to the NP region, with fewer studies on injectable AF cell delivery biomaterials. There was no consensus on ideal cell type or biomaterial carrier choice; however, MSCs and natural biomaterials (*e.g.* alginate, collagen, hyaluronic acid and fibrin) were most commonly investigated in the reviewed studies. Since the IVD is a load-bearing tissue, the biological and biomechanical function of experimental cell delivery biomaterials were summarised, yet only a subset of studies reported biomechanical outcomes. Most of these studies only reported *in vitro* compressive and viscoelastic testing of the hydrogel without reporting *in situ* biomechanical performance (*e.g. ex vivo* or *in vivo* failure testing), which is necessary to fully assess a strategy's herniation risk and reparative capacity. When analysing the subset of IVD cell delivery biomaterial studies that reported compressive and shear moduli, 2 themes were identified. First was a 'seesaw' effect, whereby biomechanical competence competed with the biological performance of a given biomaterial strategy. Second was the maturation of low-modulus biomaterial constructs by ECM synthesis that outpaces biomaterial degradation. A clear opportunity still exists for next-generation injectable IVD cell delivery biomaterial strategies that can provide initial biomechanical competence while promoting sufficient biological performance to enable construct maturation for long-term tissue healing.

Acknowledgements

This work was supported by the National Institute of Arthritis and Musculoskeletal and Skin Diseases [NIH R01 AR057397]. Authors thank Samantha Walsh, Manager of Information and Education Services for the Levy Library – Icahn School of Medicine at Mount Sinai, for important consultations regarding properly conducting systematic reviews. Authors also thank Jill Gregory, Associate Director of the Levy Library Instructional Technology Group and Certified Medical Illustrator, for medical illustrations.

Appendix A

MEDLINE Ovid and Embase Ovid search queries

1. exp biocompatible materials/
2. exp hydrogels/
3. exp gels/
4. exp tissue scaffolds/
5. exp polymers/
6. exp microspheres/
7. biomaterial*.mp.
8. scaffold*.mp.
9. hydrogel*.mp.
10. gel*.mp.
11. polymer*.mp.
12. biocompatible.mp.
13. bead*.mp.
14. sphere*.mp.
15. 1 or 2 or 3 or 4 or 5 or 7 or 8 or 9 or 10 or 11 or 12 or 13 or 14
16. exp intervertebral disc/
17. exp intervertebral disc degeneration/
18. intervertebral*.mp.
19. nucleus pulposus.mp.
20. annulus fibrosus.mp.
21. 16 or 17 or 18 or 19 or 20
22. exp "cell- and tissue-based therapy"/
23. exp guided tissue regeneration/
24. exp tissue engineering/
25. exp regenerative medicine/
26. regen*.mp.
27. tissue engineering.mp.
28. repair.mp.
29. cell*.mp.
30. therap*.mp.
31. deliver*.mp.
32. seed*.mp.
33. implant*.mp.
34. carr*.mp.
35. vehicle*.mp.
36. laden.mp.
37. 30 or 31 or 32 or 33 or 34 or 35 or 36
38. 29 and 37
39. 22 or 23 or 24 or 25 or 38
40. 15 and 21 and 39

References

Adams MA, Dolan P (2012) Intervertebral disc degeneration: evidence for two distinct phenotypes. J Anat. **221**: 497-506.

Adams MA, Roughley PJ (2006) What is intervertebral disc degeneration, and what causes it? Spine (Phila Pa 1976) **31**: 2151-2161.

Alinejad Y, Adoungotchodo A, Grant MP, Epure LM, Antoniou J, Mwale F, Lerouge S (2019) Injectable chitosan hydrogels with enhanced mechanical properties for nucleus pulposus regeneration. Tissue Eng Part A **25**: 303-313.

Alinejad Y, Adoungotchodo A, Hui E, Zehtabi F, Lerouge S (2018) An injectable chitosan/chondroitin sulfate hydrogel with tunable mechanical properties for cell therapy/tissue engineering. Int J Biol Macromol **113**: 132-141.

Alini M, Eisenstein SM, Ito K, Little C, Kettler AA, Masuda K, Melrose J, Ralphs J, Stokes I, Wilke HJ (2008) Are animal models useful for studying human disc disorders/degeneration? Eur Spine J **17**: 2-19.

Antoniou J, Steffen T, Nelson F, Winterbottom N, Hollander AP, Poole RA, Aebi M, Alini M (1996) The human lumbar intervertebral disc: evidence for changes in the biosynthesis and denaturation of the extracellular matrix with growth, maturation, ageing, and degeneration. J Clin Invest **98**: 996-1003.

Armstrong L, Lako M, Buckley N, Lappin TRJ, Murphy MJ, Nolta JA, Pittenger M, Stojkovic M (2012) Editorial: Our top 10 developments in stem cell biology over the last 30 years. Stem Cells **30**: 2-9.

Bailey A, Araghi A, Blumenthal S, Huffmon GV, Anular Repair Clinical Study Group (2013) Prospective, multicenter, randomized, controlled study of anular repair in lumbar discectomy: two-year follow-up. Spine (Phila Pa 1976) **38**: 1161-1169.

Barreto-Henriksson H, Llorente M, Larsson A, Brisby H, Gold J, Schuster E, Ström A (2019) Determination of mechanical and rheological properties of a cell-loaded peptide gel during ECM production. Int J Pharm **563**: 437-444.

Beckstein JC, Sen S, Schaer TP, Vresilovic EJ, Elliott DM (2008) Comparison of animal discs used in disc research to human lumbar disc. Spine (Phila Pa 1976) **33**: E166-E173.

Benneker LM, Andersson G, Iatridis JC, Sakai D, Härtl R, Ito K, Grad S (2014) Cell therapy for intervertebral disc repair: advancing cell therapy from bench to clinics. Eur Cell Mater **27**: 5-11.

Bhattacharjee M, Miot S, Gorecka A, Singha K, Loparic M, Dickinson S, Das A, Bhavesh NS, Ray AR, Martin I, Ghosh S (2012) Oriented lamellar silk fibrous scaffolds to drive cartilage matrix orientation: towards annulus fibrosus tissue engineering. Acta Biomater **8**: 3313-3325.

Bowles RD, Setton LA (2017) Biomaterials for intervertebral disc regeneration and repair. Biomaterials **129**: 54-67.

Brown AC, Barker TH (2014) Fibrin-based biomaterials: modulation of macroscopic properties through rational design at the molecular level. Acta Biomater **10**: 1502-1514.

Burdick JA, Mauck RL, Gerecht S (2016) To serve and protect: hydrogels to improve stem cell-based therapies. Cell Stem Cell **18**: 13-15.

Calderon L, Collin E, Velasco-Bayon D, Murphy M, O'Halloran D, Pandit A (2010) Type II collagen-hyaluronan hydrogel – a step towards a scaffold for intervertebral disc tissue engineering. Eur Cell Mater **20**: 134-148.

Cao N, Chen XB, Schreyer DJ (2012) Influence of calcium ions on cell survival and proliferation in the context of an alginate hydrogel. Int Sch Res Notices Chem Eng **2012**: 1-9.

Carragee EJ, Han MY, Suen PW, Kim D (2003) Clinical outcomes after lumbar discectomy for sciatica: the effects of fragment type and anular competence. J Bone Joint Surg Am **85**: 102-108.

Chou AI, Akintoye SO, Nicoll SB (2009) Photo-crosslinked alginate hydrogels support enhanced matrix accumulation by nucleus pulposus cells *in vivo*. Osteoarthritis Cartilage **17**: 1377-1384.

Chou AI, Nicoll SB (2009) Characterization of photocrosslinked alginate hydrogels for nucleus pulposus cell encapsulation. J Biomed Mater Res A **91**: 187-194.

Clouet J, Fusellier M, Camus A, Le Visage C, Guicheux J (2019) Intervertebral disc regeneration: From cell therapy to the development of novel bioinspired endogenous repair strategies. Adv Drug Deliv Rev **146**: 306-324.

Cruise GM, Scharp DS, Hubbell JA (1998) Characterization of permeability and network structure of interfacially photopolymerized poly(ethylene glycol) diacrylate hydrogels. Biomaterials **19**: 1287-1294.

Cruz MA, Hom WW, DiStefano TJ, Merrill R, Torre OM, Lin HA, Hecht AC, Illien-Junger S, Iatridis JC (2018) Cell-seeded adhesive biomaterial for repair of annulus fibrosus defects in intervertebral discs. Tissue Eng Part A **24**: 187-198.

Cukierman E, Pankov R, Stevens DR, Yamada KM (2001) Taking cell-matrix adhesions to the third dimension. Science **294**: 1708-1712.

Daly C, Ghosh P, Jenkin G, Oehme D, Goldschlager T (2016) A review of animal models of intervertebral disc degeneration: pathophysiology, regeneration, and translation to the clinic. Biomed Res Int **2016**: 5952165. DOI: 10.1155/2016/5952165.

DePalma MJ, Ketchum JM, Saullo T (2011) What is the source of chronic low back pain and does age play a role? Pain Med **12**: 224-233.

D'Este M, Eglin D, Alini M (2018) Lessons to be learned and future directions for intervertebral disc biomaterials. Acta Biomater **78**: 13-22.

Dieleman JL, Cao J, Chapin A, Chen C, Li Z, Liu A, Horst C, Kaldjian A, Matyasz T, Scott KW, Bui AL, Campbell M, Duber HC, Dunn AC, Flaxman AD, Fitzmaurice C, Naghavi M, Sadat N, Shieh P, Squires E, Yeung K, Murray CJL (2020) US health care

spending by payer and health condition, 1996-2016. JAMA **323**: 863-884.

Duance VC, Crean JK, Sims TJ, Avery N, Smith S, Menage J, Eisenstein SM, Roberts S (1998) Changes in collagen cross-linking in degenerative disc disease and scoliosis. Spine (Phila Pa 1976) **23**: 2545-2551.

Eyre DR, Muir H (1976) Types I and II collagens in intervertebral disc. Interchanging radial distributions in annulus fibrosus. Biochem J **157**: 267-270.

Fan C, Wang D-A (2017) Macroporous hydrogel scaffolds for three-dimensional cell culture and tissue engineering. Tissue Eng Part B Rev **23**: 451-461.

Fearing BV, Hernandez PA, Setton LA, Chahine NO (2018) Mechanotransduction and cell biomechanics of the intervertebral disc. JOR Spine **1**: e1026. DOI: 10.1002/jsp2.1026.

Foss BL, Maxwell TW, Deng Y (2014) Chondroprotective supplementation promotes the mechanical properties of injectable scaffold for human nucleus pulposus tissue engineering. J Mech Behav Biomed Mater **29**: 56-67.

Foster NE, Anema JR, Cherkin D, Chou R, Cohen SP, Gross DP, Ferreira PH, Fritz JM, Koes BW, Peul W, Turner JA, Maher CG, Lancet Low Back Pain Series Working Group (2018) Prevention and treatment of low back pain: evidence, challenges, and promising directions. Lancet **391**: 2368-2383.

Francisco AT, Hwang PY, Jeong CG, Jing L, Chen J, Setton LA (2014) Photocrosslinkable laminin-functionalized polyethylene glycol hydrogel for intervertebral disc regeneration. Acta Biomater **10**: 1102-1111.

Frith JE, Cameron AR, Menzies DJ, Ghosh P, Whitehead DL, Gronthos S, Zannettino ACW, Cooper-White JJ (2013) An injectable hydrogel incorporating mesenchymal precursor cells and pentosan polysulphate for intervertebral disc regeneration. Biomaterials **34**: 9430-9440.

Frith JE, Menzies DJ, Cameron AR, Ghosh P, Whitehead DL, Gronthos S, Zannettino ACW, Cooper-White JJ (2014) Effects of bound *versus* soluble pentosan polysulphate in PEG/HA-based hydrogels tailored for intervertebral disc regeneration. Biomaterials **35**: 1150-1162.

Gan Y, Li P, Wang L, Mo X, Song L, Xu Y, Zhao C, Ouyang B, Tu B, Luo L, Zhu L, Dong S, Li F, Zhou Q (2017) An interpenetrating network-strengthened and toughened hydrogel that supports cell-based nucleus pulposus regeneration. Biomaterials **136**: 12-28.

Gantenbein B, Illien-Jünger S, Chan SCW, Walser J, Haglund L, Ferguson SJ, Iatridis JC, Grad S (2015) Organ culture bioreactors – platforms to study human intervertebral disc degeneration and regenerative therapy. Curr Stem Cell Res Ther **10**: 339-352.

Geisler FH, McAfee PC, Banco RJ, Blumenthal SL, Guyer RD, Holt RT, Majd ME (2009) Prospective, randomized, multicenter FDA IDE study of CHARITÉ artificial disc *versus* lumbar fusion: effect at 5-year follow-up of prior surgery and prior discectomy on clinical outcomes following lumbar arthroplasty. SAS J **3**: 17-25.

Ghiselli G, Wang JC, Bhatia NN, Hsu WK, Dawson EG (2004) Adjacent segment degeneration in the lumbar spine. J Bone Joint Surg Am **86**: 1497-1503.

Gilchrist CL, Darling EM, Chen J, Setton LA (2011) Extracellular matrix ligand and stiffness modulate immature nucleus pulposus cell-cell interactions. PLoS One **6**: e27170. DOI :10.1371/journal.pone.0027170.

Goins ML, Wimberley DW, Yuan PS, Fitzhenry LN, Vaccaro AR (2005) Nucleus pulposus replacement: an emerging technology. Spine J **5**: 317S-324S.

Gray DT, Deyo RA, Kreuter W, Mirza SK, Heagerty PJ, Comstock BA, Chan L (2006) Population-based trends in volumes and rates of ambulatory lumbar spine surgery. Spine (Phila Pa 1976) **31**: 1957-1963.

Growney Kalaf EA, Flores R, Bledsoe JG, Sell SA (2016) Characterization of slow-gelling alginate hydrogels for intervertebral disc tissue-engineering applications. Mater Sci Eng C Mater Biol Appl **63**: 198-210.

Gupta MS, Cooper ES, Nicoll SB (2011) Transforming growth factor-beta 3 stimulates cartilage matrix elaboration by human marrow-derived stromal cells encapsulated in photocrosslinked carboxymethylcellulose hydrogels: potential for nucleus pulposus replacement. Tissue Eng Part A **17**: 2903-2910.

Gupta MS, Nicoll SB (2014) Functional nucleus pulposus-like matrix assembly by human mesenchymal stromal cells is directed by macromer concentration in photocrosslinked carboxymethylcellulose hydrogels. Cell Tissue Res **358**: 527-539.

Gupta MS, Nicoll SB (2015) Duration of TGF-β3 exposure impacts the chondrogenic maturation of human MSCs in photocrosslinked carboxymethylcellulose hydrogels. Ann Biomed Eng **43**: 1145-1157.

Guterl CC, See EY, Blanquer SBG, Pandit A, Ferguson SJ, Benneker LM, Grijpma DW, Sakai D, Eglin D, Alini M, Iatridis JC, Grad S (2013) Challenges and strategies in the repair of ruptured annulus fibrosus. Eur Cell Mater **25**: 1-21.

Halloran DO, Grad S, Stoddart M, Dockery P, Alini M, Pandit AS (2008) An injectable cross-linked scaffold for nucleus pulposus regeneration. Biomaterials **29**: 438-447.

Hartvigsen J, Hancock MJ, Kongsted A, Louw Q, Ferreira ML, Genevay S, Hoy D, Karppinen J, Pransky G, Sieper J, Smeets RJ, Underwood M, Lancet Low Back Pain Series Working Group (2018) What low back pain is and why we need to pay attention. Lancet **391**: 2356-2367.

Hayami JWS, Waldman SD, Amsden BG (2011) A photocurable hydrogel/elastomer composite scaffold with bi-continuous morphology for cell encapsulation. Macromol Biosci **11**: 1672-1683.

Hayami JWS, Waldman SD, Amsden BG (2013) Injectable, high modulus, and fatigue resistant composite scaffold for load-bearing soft tissue regeneration. Biomacromolecules **14**: 4236-4247.

Hayami JWS, Waldman SD, Amsden BG (2016) Chondrocyte generation of cartilage-like tissue following photoencapsulation in methacrylated polysaccharide solution blends. Macromol Biosci **16**: 1083-1095.

Hoy D, March L, Brooks P, Blyth F, Woolf A, Bain C, Williams G, Smith E, Vos T, Barendregt J, Murray C, Burstein R, Buchbinder R (2014) The global burden of low back pain: estimates from the Global Burden of Disease 2010 study. Ann Rheum Dis **73**: 968-974.

Hu A, Xing R, Jiang L, Li Z, Liu P, Wang H, Li X, Dong J (2020) Thermosensitive hydrogels loaded with human-induced pluripotent stem cells overexpressing growth differentiation factor-5 ameliorate intervertebral disc degeneration in rats. J Biomed Mater Res B Appl Biomater **108**: 2005-2016.

Hu B, He R, Ma K, Wang Z, Cui M, Hu H, Rai S, Wang B, Shao Z (2018) Intervertebral disc-derived stem/progenitor cells as a promising cell source for intervertebral disc regeneration. Stem Cells Int **2018**: 7412304. DOI: 10.1155/2018/7412304.

Iatridis JC, Nicoll SB, Michalek AJ, Walter BA, Gupta MS (2013) Role of biomechanics in intervertebral disc degeneration and regenerative therapies: what needs repairing in the disc and what are promising biomaterials for its repair? Spine J **13**: 243-262.

Jacobs NT, Cortes DH, Vresilovic EJ, Elliott DM (2013) Biaxial tension of fibrous tissue: using finite element methods to address experimental challenges arising from boundary conditions and anisotropy. J Biomech Eng **135**: 021004. DOI: 10.1115/1.4023503.

Jiang L, Sun Z, Chen X, Li J, Xu Y, Zu Y, Hu J, Han D, Yang C (2016) Cells sensing mechanical cues: stiffness influences the lifetime of cell-extracellular matrix interactions by affecting the loading rate. ACS Nano **10**: 207-217.

Khang G, Lee SK, Kim HN, Silva-Correia J, Gomes ME, Viegas CAA, Dias IR, Oliveira JM, Reis RL (2015) Biological evaluation of intervertebral disc cells in different formulations of gellan gum-based hydrogels. J Tissue Eng Regen Med **9**: 265-275.

Kim DH, Martin JT, Elliott DM, Smith LJ, Mauck RL (2015) Phenotypic stability, matrix elaboration and functional maturation of nucleus pulposus cells encapsulated in photocrosslinkable hyaluronic acid hydrogels. Acta Biomater **12**: 21-29.

Kong HJ, Alsberg E, Kaigler D, Lee KY, Mooney DJ (2004) Controlling degradation of hydrogels *via* the size of cross-linked junctions. Adv Mater **16**: 1917-1921.

Kroeber MW, Unglaub F, Wang H, Schmid C, Thomsen M, Nerlich A, Richter W (2002) New *in vivo* animal model to create intervertebral disc degeneration and to investigate the effects of therapeutic strategies to stimulate disc regeneration. Spine (Phila Pa 1976) **27**: 2684-2690.

Kumar D, Gerges I, Tamplenizza M, Lenardi C, Forsyth NR, Liu Y (2014) Three-dimensional hypoxic culture of human mesenchymal stem cells encapsulated in a photocurable, biodegradable polymer hydrogel: a potential injectable cellular product for nucleus pulposus regeneration. Acta Biomater **10**: 3463-3474.

Langer R, Vacanti JP (1993) Tissue engineering. Science **260**: 920-926.

Li Z, Kaplan KM, Wertzel A, Peroglio M, Amit B, Alini M, Grad S, Yayon A (2014) Biomimetic fibrin-hyaluronan hydrogels for nucleus pulposus regeneration. Regen Med **9**: 309-326.

Ligorio C, Zhou M, Wychowaniec JK, Zhu X, Bartlam C, Miller AF, Vijayaraghavan A, Hoyland JA, Saiani A (2019) Graphene oxide containing self-assembling peptide hybrid hydrogels as a potential 3D injectable cell delivery platform for intervertebral disc repair applications. Acta Biomater **92**: 92-103.

Lin HA, Gupta MS, Varma DM, Gilchrist ML, Nicoll SB (2016) Lower crosslinking density enhances functional nucleus pulposus-like matrix elaboration by human mesenchymal stem cells in carboxymethylcellulose hydrogels. J Biomed Mater Res A A **104**: 165-177.

Livshits G, Popham M, Malkin I, Sambrook PN, Macgregor AJ, Spector T, Williams FMK (2011) Lumbar disc degeneration and genetic factors are the main risk factors for low back pain in women: the UK Twin Spine Study. Ann Rheum Dis **70**: 1740-1745.

Long RG, Torre OM, Hom WW, Assael DJ, Iatridis JC (2016) Design requirements for annulus fibrosus repair: review of forces, displacements, and material properties of the intervertebral disk and a summary of candidate hydrogels for repair. J Biomech Eng **138**: 021007. DOI: 10.1115/1.4032353.

Lurie JD, Tosteson TD, Tosteson ANA, Zhao W, Morgan TS, Abdu WA, Herkowitz H, Weinstein JN (2014) Surgical *versus* nonoperative treatment for lumbar disc herniation: eight-year results for the spine patient outcomes research trial. Spine (Phila Pa 1976) **39**: 3-16.

Maroudas A, Stockwell RA, Nachemson A, Urban J (1975) Factors involved in the nutrition of the human lumbar intervertebral disc: cellularity and diffusion of glucose *in vitro*. J Anat **120**: 113-130.

Mercuri JJ, Patnaik S, Dion G, Gill SS, Liao J, Simionescu DT (2013) Regenerative potential of decellularized porcine nucleus pulposus hydrogel scaffolds: stem cell differentiation, matrix remodeling, and biocompatibility studies. Tissue Eng Part A **19**: 952-966.

Merino S, Martín C, Kostarelos K, Prato M, Vázquez E (2015) Nanocomposite hydrogels: 3D polymer-nanoparticle synergies for on-demand drug delivery. ACS Nano **9**: 4686-4697.

Miele VJ, Panjabi MM, Benzel EC (2012) Anatomy and biomechanics of the spinal column and cord. Handb Clin Neurol **109**: 31-43.

Moher D, Liberati A, Tetzlaff J, Altman DG, PRISMA Group (2009) Preferred reporting items for systematic reviews and meta-analyses: the PRISMA statement. PLoS Med **6**: e1000097. DOI: 10.1371/journal.pmed.1000097.

Moss IL, Gordon L, Woodhouse KA, Whyne CM, Yee AJM (2011) A novel thiol-modified hyaluronan and elastin-like polypetide composite material for tissue engineering of the nucleus pulposus of the intervertebral disc. Spine (Phila Pa 1976) **36**: 1022-1029.

National Guideline Centre (UK) (2016) Low back pain and sciatica in over 16s: assessment and management. NICE guideline (www.nice.org.uk/guidance/ng59) 20pp, London: National Institute for Health and Care Excellence (UK).

Navaro Y, Bleich-Kimelman N, Hazanov L, Mironi-Harpaz I, Shachaf Y, Garty S, Smith Y, Pelled G, Gazit D, Seliktar D, Gazit Z (2015) Matrix stiffness determines the fate of nucleus pulposus-derived stem cells. Biomaterials **49**: 68-76.

Nerlich AG, Schleicher ED, Boos N (1997) 1997 Volvo Award winner in basic science studies. Immunohistologic markers for age-related changes of human lumbar intervertebral discs. Spine (Phila Pa 1976) **22**: 2781-2795.

Nerurkar NL, Elliott DM, Mauck RL (2007) Mechanics of oriented electrospun nanofibrous scaffolds for annulus fibrosus tissue engineering. J Orthop Res **25**: 1018-1028.

Nerurkar NL, Elliott DM, Mauck RL (2010) Mechanical design criteria for intervertebral disc tissue engineering. J. Biomech **43**: 1017-1030.

Ohtori S, Inoue G, Miyagi M, Takahashi K (2015) Pathomechanisms of discogenic low back pain in humans and animal models. Spine J **15**: 1347-1355.

Oryan A, Kamali A, Moshiri A, Baharvand H, Daemi H (2018) Chemical crosslinking of biopolymeric scaffolds: current knowledge and future directions of crosslinked engineered bone scaffolds. Int J Biol Macromol **107**: 678-688.

Pampaloni F, Reynaud EG, Stelzer EHK (2007) The third dimension bridges the gap between cell culture and live tissue. Nat Rev Mol Cell Biol **8**: 839-845.

Panebianco CJ, DiStefano TJ, Mui B, Hom WW, Iatridis JC (2020) Crosslinker concentration controls TGFβ-3 release and annulus fibrosus cell apoptosis in genipin-crosslinked fibrin hydrogels. Eur Cell Mater **39**: 211-226.

Parisi L, Toffoli A, Ghiacci G, Macaluso GM (2018) Tailoring the interface of biomaterials to design effective scaffolds. J Funct Biomater **9**: 50. DOI: 10.3390/jfb9030050.

Park S-H, Cho H, Gil ES, Mandal BB, Min B-H, Kaplan DL (2011) Silk-fibrin/hyaluronic acid composite gels for nucleus pulposus tissue regeneration. Tissue Eng Part A **17**: 2999-3009.

Park S-H, Gil ES, Mandal BB, Cho H, Kluge JA, Min B-H, Kaplan DL (2012) Annulus fibrosus tissue engineering using lamellar silk scaffolds. J Tissue Eng Regen Med **6 Suppl 3**: s24-33.

Parker SL, Grahovac G, Vukas D, Vilendecic M, Ledic D, McGirt MJ, Carragee EJ (2016) Effect of an annular closure device (Barricaid) on same-level recurrent disk herniation and disk height loss after primary lumbar discectomy: two-year results of a multicenter prospective cohort study. Clin Spine Surg **29**: 454-460.

Parker SL, Mendenhall SK, Godil SS, Sivasubramanian P, Cahill K, Ziewacz J, McGirt MJ (2015) Incidence of low back pain after lumbar discectomy for herniated disc and its effect on patient-reported outcomes. Clin Orthop Relat Res **473**: 1988-1999.

Pattappa G, Li Z, Peroglio M, Wismer N, Alini M, Grad S (2012) Diversity of intervertebral disc cells: phenotype and function. J Anat **221**: 480-496.

Pereira DR, Silva-Correia J, Oliveira JM, Reis RL, Pandit A, Biggs MJ (2018) Nanocellulose reinforced gellan-gum hydrogels as potential biological substitutes for annulus fibrosus tissue regeneration. Nanomedicine **14**: 897-908.

Pereira DR, Silva-Correia J, Caridade SG, Oliveira JT, Sousa RA, Salgado AJ, Oliveira JM, Mano JF, Sousa N, Reis RL (2011) Development of gellan gum-based microparticles/hydrogel matrices for application in the intervertebral disc regeneration. Tissue Eng Part C Methods **17**: 961-972.

Peroglio M, Grad S, Mortisen D, Sprecher CM, Illien-Jünger S, Alini M, Eglin D (2012) Injectable thermoreversible hyaluronan-based hydrogels for nucleus pulposus cell encapsulation. Eur. Spine J. **21 Suppl 6**: S839-S849.

Pfannkuche J-J, Guo W, Cui S, Ma J, Lang G, Peroglio M, Richards RG, Alini M, Grad S, Li Z (2020) Intervertebral disc organ culture for the investigation of disc pathology and regeneration – benefits, limitations, and future directions of bioreactors. Connect Tissue Res **61**: 304-321.

Phillips FM, Slosar PJ, Youssef JA, Andersson G, Papatheofanis F (2013) Lumbar spine fusion for chronic low back pain due to degenerative disc disease: a systematic review. Spine (Phila Pa 1976) **38**: E409-E422.

Piluso S, Labet M, Zhou C, Seo JW, Thielemans W, Patterson J (2019) Engineered three-dimensional microenvironments with starch nanocrystals as cell-instructive materials. Biomacromolecules **20**: 3819-3830.

Qaseem A, Wilt TJ, McLean RM, Forciea MA, Clinical Guidelines Committee of the American College of Physicians (2017) Noninvasive treatments for acute, subacute, and chronic low back pain: a clinical practice guideline from the American College of Physicians. Ann Intern Med **166**: 514-530.

Reza AT, Nicoll SB (2010a) Characterization of novel photocrosslinked carboxymethylcellulose hydrogels for encapsulation of nucleus pulposus cells. Acta Biomater **6**: 179-186.

Reza AT, Nicoll SB (2010b) Serum-free, chemically defined medium with TGF-β(3) enhances functional properties of nucleus pulposus cell-laden carboxymethylcellulose hydrogel constructs. Biotechnol Bioeng **105**: 384-395.

Richardson SM, Hoyland JA, Mobasheri R, Csaki C, Shakibaei M, Mobasheri A (2010) Mesenchymal stem cells in regenerative medicine: opportunities and

challenges for articular cartilage and intervertebral disc tissue engineering. J Cell Physiol **222**: 23-32.

Richardson SM, Kalamegam G, Pushparaj PN, Matta C, Memic A, Khademhosseini A, Mobasheri R, Poletti FL, Hoyland JA, Mobasheri A (2016) Mesenchymal stem cells in regenerative medicine: focus on articular cartilage and intervertebral disc regeneration. Methods **99**: 69-80.

Risbud MV, Guttapalli A, Tsai T-T, Lee JY, Danielson KG, Vaccaro AR, Albert TJ, Gazit Z, Gazit D, Shapiro IM (2007) Evidence for skeletal progenitor cells in the degenerate human intervertebral disc. Spine (Phila Pa 1976) **32**: 2537-2544.

Rubin DI (2007) Epidemiology and risk factors for spine pain. Neurol Clin **25**: 353-371.

Saito H, Murabayashi S, Mitamura Y, Taguchi T (2008) Characterization of alkali-treated collagen gels prepared by different crosslinkers. J Mater Sci Mater Med **19**: 1297-1305.

Sakai D (2011) Stem cell regeneration of the intervertebral disk. Orthop Clin North Am **42**: 555-562.

Sato M, Asazuma T, Ishihara M, Kikuchi T, Masuoka K, Ichimura S, Kikuchi M, Kurita A, Fujikawa K (2003) An atelocollagen honeycomb-shaped scaffold with a membrane seal (ACHMS-scaffold) for the culture of annulus fibrosus cells from an intervertebral disc. J Biomed Mater Res A **64**: 248-256.

Schol J, Sakai D (2019) Cell therapy for intervertebral disc herniation and degenerative disc disease: clinical trials. Int Orthop **43**: 1011-1025.

Schroeder JE, Dettori JR, Brodt ED, Kaplan L (2012) Disc degeneration after disc herniation: are we accelerating the process? Evid Based Spine Care J **3**: 33-40.

Shapiro IM, Risbud MV (2014) The intervertebral disc: molecular and structural studies of the disc in health and disease. Springer-Verlag Wien, pp446.

Siepe CJ, Heider F, Wiechert K, Hitzl W, Ishak B, Mayer MH (2014) Mid- to long-term results of total lumbar disc replacement: a prospective analysis with 5- to 10-year follow-up. Spine J **14**: 1417-1431.

Silberberg R, Aufdermaur M, Adler JH (1979) Degeneration of the intervertebral disks and spondylosis in aging sand rats. Arch Pathol Lab Med **103**: 231-235.

Silva-Correia J, Gloria A, Oliveira MB, Mano JF, Oliveira JM, Ambrosio L, Reis RL (2013) Rheological and mechanical properties of acellular and cell-laden methacrylated gellan gum hydrogels. J Biomed Mater Res A **101**: 3438-3446.

Smith LJ, Silverman L, Sakai D, Le Maitre CL, Mauck RL, Malhotra NR, Lotz JC, Buckley CT (2018) Advancing cell therapies for intervertebral disc regeneration from the lab to the clinic: Recommendations of the ORS spine section. JOR Spine **1**: e1036. DOI: 10.1002/jsp2.1036.

Stern S, Lindenhayn K, Schultz O, Perka C (2000) Cultivation of porcine cells from the nucleus pulposus in a fibrin/hyaluronic acid matrix. Acta Orthop Scand **71**: 496-502.

Stochkendahl MJ, Kjaer P, Hartvigsen J, Kongsted A, Aaboe J, Andersen M, Andersen MØ, Fournier G, Højgaard B, Jensen MB, Jensen LD, Karbo T, Kirkeskov L, Melbye M, Morsel-Carlsen L, Nordsteen J, Palsson TS, Rasti Z, Silbye PF, Steiness MZ, Tarp S, Vaagholt M (2018) National clinical guidelines for non-surgical treatment of patients with recent onset low back pain or lumbar radiculopathy. Eur Spine J **27**: 60-75.

Tao H, Wu Y, Li H, Wang C, Zhang Y, Li C, Wen T, Wang X, He Q, Wang D, Ruan D (2015) BMP7-based functionalized self-assembling peptides for nucleus pulposus tissue engineering. ACS Appl Mater Interfaces **7**: 17076-17087.

Toh WS, Lim TC, Kurisawa M, Spector M (2012) Modulation of mesenchymal stem cell chondrogenesis in a tunable hyaluronic acid hydrogel microenvironment. Biomaterials **33**: 3835-3845.

Torre OM, Mroz V, Bartelstein MK, Huang AH, Iatridis JC (2019) Annulus fibrosus cell phenotypes in homeostasis and injury: implications for regenerative strategies. Ann N Y Acad Sci **1442**: 61-78.

Tsantrizos A, Ito K, Aebi M, Steffen T (2005) Internal strains in healthy and degenerated lumbar intervertebral discs. Spine (Phila Pa 1976) **30**: 2129-2137.

Ullah F, Othman MBH, Javed F, Ahmad Z, Md Akil H (2015) Classification, processing and application of hydrogels: a review. Mater Sci Eng C Mater Biol Appl **57**: 414-433.

Vadalà G, Sowa G, Hubert M, Gilbertson LG, Denaro V, Kang JD (2012) Mesenchymal stem cells injection in degenerated intervertebral disc: cell leakage may induce osteophyte formation. J Tissue Eng Regen Med **6**: 348-355.

Varma DM, DiNicolas MS, Nicoll SB (2018) Injectable, redox-polymerized carboxymethylcellulose hydrogels promote nucleus pulposus-like extracellular matrix elaboration by human MSCs in a cell density-dependent manner. J Biomater Appl **33**: 576-589.

Vedadghavami A, Minooei F, Mohammadi MH, Khetani S, Rezaei Kolahchi A, Mashayekhan S, Sanati-Nezhad A (2017) Manufacturing of hydrogel biomaterials with controlled mechanical properties for tissue engineering applications. Acta Biomater **62**: 42-63.

Virk S, Chen T, Meyers KN, Lafage V, Schwab F, Maher SA (2020) Comparison of biomechanical studies of disc repair devices based on a systematic review. Spine J **20**: 1344-1355.

Vos T, Allen C, Arora M (2016) Global, regional, and national incidence, prevalence, and years lived with disability for 310 diseases and injuries, 1990-2015: a systematic analysis for the Global Burden of Disease Study 2015. Lancet **388**: 1545-1602.

Wan S, Borland S, Richardson SM, Merry CLR, Saiani A, Gough JE (2016) Self-assembling peptide

hydrogel for intervertebral disc tissue engineering. Acta Biomater **46**: 29-40.

Wang B, Sun C, Shao Z, Yang S, Che B, Wu Q, Liu J (2014) Designer self-assembling peptide nanofiber scaffolds containing link protein N-terminal peptide induce chondrogenesis of rabbit bone marrow stem cells. Biomed Res Int **2014**: 421954. DOI: 10.1155/2014/421954.

Wang F, Nan L-P, Zhou S-F, Liu Y, Wang Z-Y, Wang J-C, Feng X-M, Zhang L (2019) Injectable hydrogel combined with nucleus pulposus-derived mesenchymal stem cells for the treatment of degenerative intervertebral disc in rats. Stem Cells Int **2019**: 8496025. DOI: 10.1155/2019/8496025.

Xia X-P, Chen H-L, Cheng H-B (2013) Prevalence of adjacent segment degeneration after spine surgery: a systematic review and meta-analysis. Spine (Phila Pa 1976) **38**: 597-608.

Zhou X, Tao Y, Chen E, Wang J, Fang W, Zhao T, Liang C, Li F, Chen Q (2018a) Genipin-cross-linked type II collagen scaffold promotes the differentiation of adipose-derived stem cells into nucleus pulposus-like cells. J Biomed Mater Res A **106**: 1258-1268.

Zhou X, Wang J, Fang W, Tao Y, Zhao T, Xia K, Liang C, Hua J, Li F, Chen Q (2018b) Genipin cross-linked type II collagen/chondroitin sulfate composite hydrogel-like cell delivery system induces differentiation of adipose-derived stem cells and regenerates degenerated nucleus pulposus. Acta Biomater **71**: 496-509.

Zhu Y, Tan J, Zhu H, Lin G, Yin F, Wang L, Song K, Wang Y, Zhou G, Yi W (2017) Development of kartogenin-conjugated chitosan-hyaluronic acid hydrogel for nucleus pulposus regeneration. Biomater Sci **5**: 784-791.

Discussion with Reviewers

Reviewer 1: Which are the most promising cell delivery materials you suggest to further investigate and why?

Authors: The most promising IVD cell delivery biomaterials in this review are those which have demonstrated construct maturation in Tables 2 and 3. Assuming they have sufficient initial adhesive and biomechanical properties to remain within the IVD space, the fact that they have demonstrated sufficient ECM synthesis to enhance construct modulus is very promising for their ability to promote IVD repair and regeneration *ex vivo* and *in vivo*. However, this requires further investigation in appropriate preclinical models before clinical translation.

Reviewer 1: What are the main advantages and limitations of the models you reported?

Authors: This systematic review highlighted a need to measure multiple biological and biomechanical outputs at varying hierarchical scales. Most IVD cell delivery biomaterial strategies investigated only tested *in vitro*, which does not simulate the clinical repair environment. Assessing biological and biomechanical outcome measurements at various hierarchical scales will best challenge the IVD cell delivery biomaterial strategy, allow for targeted modifications and more rapidly advance to clinical translation.

Andrea Vernengo: Although crosslinking is clearly the most common way to improve mechanical properties, three articles cited in the paper (Merino *et al*., 2015; Pereira *et al*., 2018; Piluso *et al*., 2019) report increases in the mechanical properties of hydrogels with addition of micro or nanoparticles. In the latter two citations, cell response was not adversely affected by addition of the particles. Another study reported good cell viability with increased mechanical reinforcement by microparticles in ionically crosslinked gellan gum (Pereira *et al*., 2011). Could the authors offer some comments on two-phase composites, which appear to have potential in terms of balancing mechanical properties and cell performance?

Authors: Our analysis of articles which reported biomaterial moduli for a range of formulations (Table 1) showed that composite biomaterial strategies (*i.e.* strategies listed as "Other" under the "Additional Modifications" heading) generally avoided the 'seesaw' effect, which could make these types of strategies promising for balancing the biological and biomechanical demands of an IVD cell delivery biomaterial.

Andrea Vernengo: While softer hydrogels support higher cell viability and ECM expression, have they been shown to support AF phenotypes? Did the analysis reveal any relationships between hydrogel crosslinking and the expression of NP or AF related markers, at the gene or protein level?

Authors: There is evidence to show that increasing matrix stiffness downregulates NP marker gene expression (Additional reference: Fearing *et al*., 2018). Our analysis showed that there was a decline in the expression of IVD ECM genes when increasing the concentration of macromer, additional crosslinker or an additional modification. This could be a direct consequence of matrix stiffness or due to crosslinker cytotoxicity and reduced biomaterial porosity.

Additional Reference

Fearing BV, Hernandez PA, Setton LA, Chahine NO (2018) Mechanotransduction and cell biomechanics of the intervertebral disc. JOR Spine **1**: e1026. DOI: 10.1002/jsp2.1026.

Editor's note: The Scientific Editor responsible for this paper was Sibylle Grad.
